# A comprehensive WGS-based pipeline for the identification of new candidate genes in inherited retinal dystrophies

**DOI:** 10.1038/s41525-022-00286-0

**Published:** 2022-03-04

**Authors:** María González-del Pozo, Elena Fernández-Suárez, Nereida Bravo-Gil, Cristina Méndez-Vidal, Marta Martín-Sánchez, Enrique Rodríguez-de la Rúa, Manuel Ramos-Jiménez, María José Morillo-Sánchez, Salud Borrego, Guillermo Antiñolo

**Affiliations:** 1 Department of Maternofetal Medicine, Genetics and Reproduction, Institute of Biomedicine of Seville, University Hospital Virgen del Rocio/CSIC/University of Seville, Seville, Spain; 2 Centro de Investigación Biomédica en Red de Enfermedades Raras (CIBERER), Seville, Spain; 3 Department of Ophthalmology, University Hospital Virgen Macarena, Seville, Spain; 4 Retics Patologia Ocular, OFTARED, Instituto de Salud Carlos III, Madrid, Spain; 5 Department of Clinical Neurophysiology, University Hospital Virgen Macarena, Seville, Spain

**Keywords:** Genetics research, Translational research, Retinal diseases, Hereditary eye disease

## Abstract

To enhance the use of Whole Genome Sequencing (WGS) in clinical practice, it is still necessary to standardize data analysis pipelines. Herein, we aimed to define a WGS-based algorithm for the accurate interpretation of variants in inherited retinal dystrophies (IRD). This study comprised 429 phenotyped individuals divided into three cohorts. A comparison of 14 pathogenicity predictors, and the re-definition of its cutoffs, were performed using panel-sequencing curated data from 209 genetically diagnosed individuals with IRD (training cohort). The optimal tool combinations, previously validated in 50 additional IRD individuals, were also tested in patients with hereditary cancer (*n* = 109), and with neurological diseases (*n* = 47) to evaluate the translational value of this approach (validation cohort). Then, our workflow was applied for the WGS-data analysis of 14 individuals from genetically undiagnosed IRD families (discovery cohort). The statistical analysis showed that the optimal filtering combination included CADDv1.6, MAPP, Grantham, and SIFT tools. Our pipeline allowed the identification of one homozygous variant in the candidate gene *CFAP20* (c.337 C > T; p.Arg113Trp), a conserved ciliary gene, which was abundantly expressed in human retina and was located in the photoreceptors layer. Although further studies are needed, we propose *CFAP20* as a candidate gene for autosomal recessive retinitis pigmentosa. Moreover, we offer a translational strategy for accurate WGS-data prioritization, which is essential for the advancement of personalized medicine.

## Introduction

Inherited retinal dystrophies (IRD) constitute a group of clinically and genetically heterogeneous, rare Mendelian disorders that lead to irreversible and progressive visual impairment due to dysfunction or loss of photoreceptors^1^. The most common form of IRD is retinitis pigmentosa (RP, ORPHA:791) defined by the primary death of rods, which results in night blindness and constriction of the visual field^2^. To date, pathogenic variants in 89 genes can cause RP (RetNet, the Retinal Information Network, https://sph.uth.edu/retnet/, accessed January 2021), however, an estimated 40% of cases remain without a genetic diagnosis after testing for the most prevalent retinal genes, suggesting that the RP in these patients could be attributed to mutations that were either undetectable by the current methods, or not routinely analyzed, such as deep-intronic variants, complex structural variants (mobile elements insertions, inversions, translocations, etc.), or variants in yet unidentified disease genes^3–6^.

In this scenario, identifying novel disease genes or variants is important to increase the diagnostic rate and to facilitate new approaches for clinical care of IRD patients. The advances in next-generation sequencing (NGS) technologies have ushered in a new era for genetic diagnosis and disease-gene discovery^7^. Recent studies have reported the clinical utility of Whole Genome Sequencing (WGS), especially for rare diseases^8,9^, and its large expectations on personalized medicine^10^, highlighting that the use of WGS as a first diagnostic strategy could constitute a unique and powerful analysis. This approach provides a bigger evenness of coverage and the proportion of transcripts covered in their entirety compared to targeting sequencing, allowing a superior detection of structural variants, variants in non-coding regions, and detection of variants in GC-rich regions^11^. However, the clinical translation of this approach is currently limited due to its still high cost, a large amount of generated raw data, and the lack of efficient protocols for the WGS-data analysis^12,13^. Nevertheless, in recent years, the cost of generating genome information has shown a rapid decline making it possible a greater application of WGS as in the clinical research as in some health care systems^9,10^. Concerning bioinformatic processing, it is still necessary the application of advanced filters to categorize variants efficiently^10^. In this regard, deleteriousness predictors provide the opportunity to facilitate variant prioritization in WGS studies. Multiple prediction algorithms have been developed but it is still unclear which ones and how they should be applied in human disease studies to minimize both false-positive and false-negative rates^14^.

The aim of this work was to design a WGS-based pipeline for the identification of potentially pathogenic variants in a group of previously analyzed RP patients without genetic diagnosis. In this regard, we conducted a comparative study of 14 variant pathogenicity prediction tools to choose the most reliable cutoff for variants associated with IRDs. These results enabled us to optimize the filtering and prioritization of WGS data in order to rapidly obtain a dataset enriched in likely pathogenic variants. The application of our workflow allowed us to discover a variant in the *CFAP20* gene in one family. Here, we propose *CFAP20* as a new likely candidate gene for arRP.

## Results

### Establishment of the optimal cutoffs

**Fig. 1:**
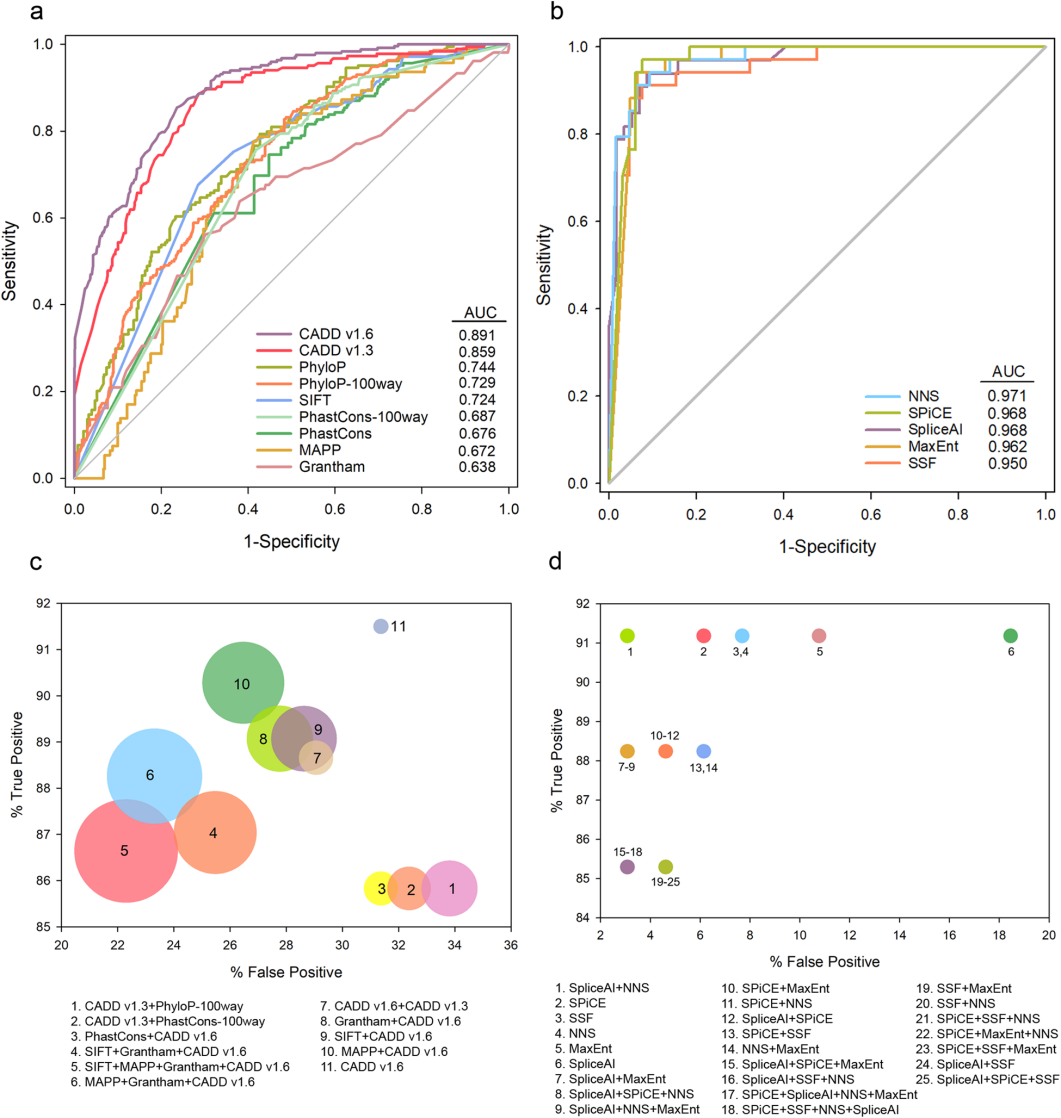
The ROC curves and combinatorial analysis results for different pathogenicity prediction tools. **a** ROC curve for the non-splicing predictors using the training dataset. **b** ROC curve for the splicing predictors using a subset of the training dataset containing only splicing variants. Higher AUC score indicates better performance. **c**, **d** Bubble plots represent the TP rate versus the FP rate for each of the different combinations of the prediction tools. Only the combinations of non-splicing predictors (**c**) and the splicing predictors (**d**) meeting the quality criteria (TP ≥ 85%, FP ≤ 35%, and Missing values ≤ 30%) were represented. In case of non-splicing predictors, the bubble size is proportional to the percentage of missing values. AUC area under the curve, FP false positive, ROC receiver operating characteristic curve, TP true positive.

The carefully curated training dataset comprised a total of 942 distinct rare SNVs located in any of the IRD associated genes, including 247 pathogenic or likely pathogenic variants and 695 benign or likely benign variants (Supplementary Table 1). ROC curves for each tool were computed using the prediction scores from the training dataset (Fig. 1A, B). Of note, a subgroup of 99 splicing variants (34 pathogenic/likely pathogenic variants and 65 benign/likely benign variants) was used for the ROC curves of the splicing predictors.

The specificities of each prediction method were evaluated according to AUC values. We found that all values were significantly >0.5 (*P*-value < 0.0001) indicating that all methods were suitable to discern between pathogenic and benign variants. For the training dataset, the predictor with a higher AUC was CADDv1.6 (AUC = 0.891) (Fig. 1A), whereas for the splicing subset the predictor with higher AUC was NNS (AUC = 0.971) (Fig. 1B).

Although three different approaches were conducted to establish the optimal cutoff for each prediction method, the optimal threshold was defined as the value in which the sensitivity is 90% for each predictor (Table 1). In order to visually compare the distribution of the filtered variants using both the cutoff most widely described in the literature and the cutoff calculated in this study, dot histograms were represented (Supplementary Fig. 1).

**Table 1:** Relevant statistical data and the optimal cutoffs for the 14 prediction tools tested in this study.

Prediction tools	AUC	Optimal cutoff	% FP	% Missing value
*Splicing tools*				
Alamut® Batch v1.11				
SPiCE	0.968	≥0.993	6.15	0.00
SSF	0.950	≥12.360	7.69	0.11
MaxEnt	0.962	≥53.580	10.77	0.74
NNS	0.971	≥62.730	7.69	0.32
Ensembl Variant Effect Predictor				
SpliceAI	0.968	≥0.405	7.02	9.09
*Non-splicing tools*				
Alamut® Batch v1.11				
PhastCons	0.676	≥0.097	69.96	8.28
PhyloP	0.744	≥0.449	58.07	8.81
SIFT	0.724	≤0.175	68.90	44.48
MAPP	0.672	≤0.098	68.37	54.78^a^
Grantham	0.638	≥28.000	88.04	44.48
Bystro genomics				
PhastCons-100way	0.687	≥0.125	62.39	8.49
PhyloP-100way	0.729	≥0.475	60.68	8.49
CADDv1.3	0.859	≥21.950	33.91	8.49
Ensembl Variant Effect Predictor				
CADDv1.6	0.891	≥22.250	31.37	0.00

The optimal cutoff and the %FP have been calculated for a Sensitivity of 90%.

AUC area under the curve, FP false positive.

^a^MAPP will not calculate scores if the gap weigh of a column is >50%.

### Optimization and validation of the discovery pipeline

As the estimated FP rates, with the exceptions of CADD and the splicing tools, were not acceptable in most cases (≥35%) (Table 1), a combinatorial analysis was carried out. For this purpose, we applied our cutoff values to filter the training dataset and calculated the TP and FP rates in each of the 109 combinatorial models (Supplementary Table 2). Thirty-six of the predictor combinations met the criteria (TP ≥ 85%, FP ≤ 35%, and Missing values ≤ 30%), including 11 non-splicing and 25 splicing tool combinations. Models passing quality filters were graphically assessed by bubble plots (Fig. 1C, D). Considering the balance between FP and TP rates, the optimal combination of splicing tools was “SpliceAI + NNS”, which presented the lowest FP rate (3.08%) with a still elevated TP rate (94.18%). On the other hand, four of non-splicing predictors: “CADDv1.6”, “CADDv1.6 + MAPP”, “CADDv1.6 + MAPP + Grantham”, and “CADDv1.6 + MAPP + Grantham + SIFT” were initially proposed as the most suitable options.

**Fig. 2:**
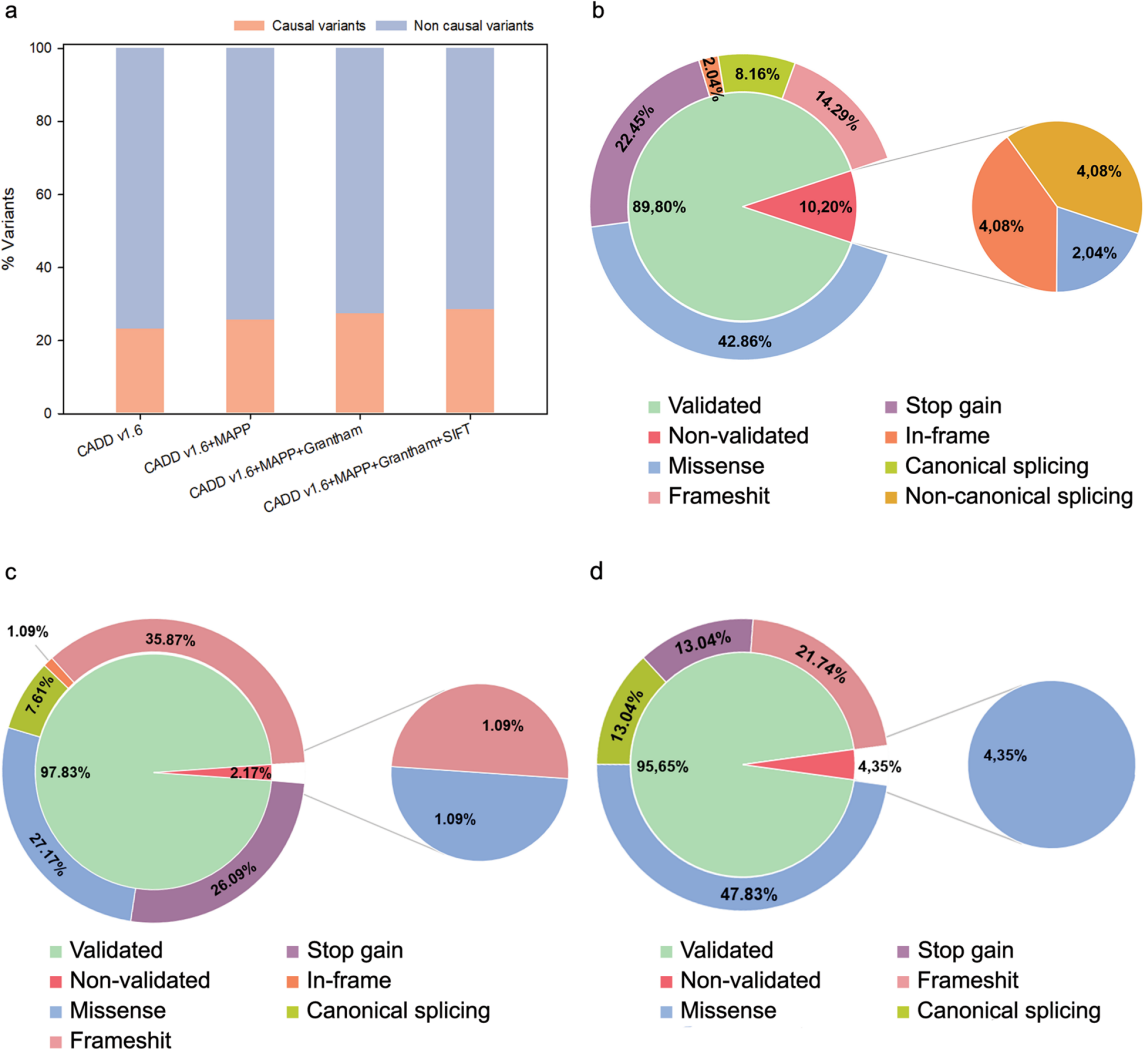
Validation of the discovery pipeline in three different inherited diseases. **a** Histograms shows the enrichment in causal variants that are recovered after applying the four best combinations of non-splicing computational tools. These data have been obtained using the IRD patient validation sub-cohort. **b–d**, Sector diagram represents the different percentages of variants validated and not validated from the IRD sub-cohort **b**, from the hereditary cancer sub-cohort **c**, and from the neurological diseases sub-cohort **d**.

To finally determine the most enriched approach in likely causal variants, the IRD validation dataset was submitted to the four combinations of the non-splicing tools. This dataset comprised a total of 5085 distinct variants in known IRD genes, including 49 pathogenic causal mutations. Taking into account the ratio of causal and non-causal variants prioritized in each model (Fig. 2A), the “CADDv1.6+MAPP + Grantham+SIFT” combination showed to be the most accurate option with enrichment of causal variants of 28.57%.

**Fig. 3:**
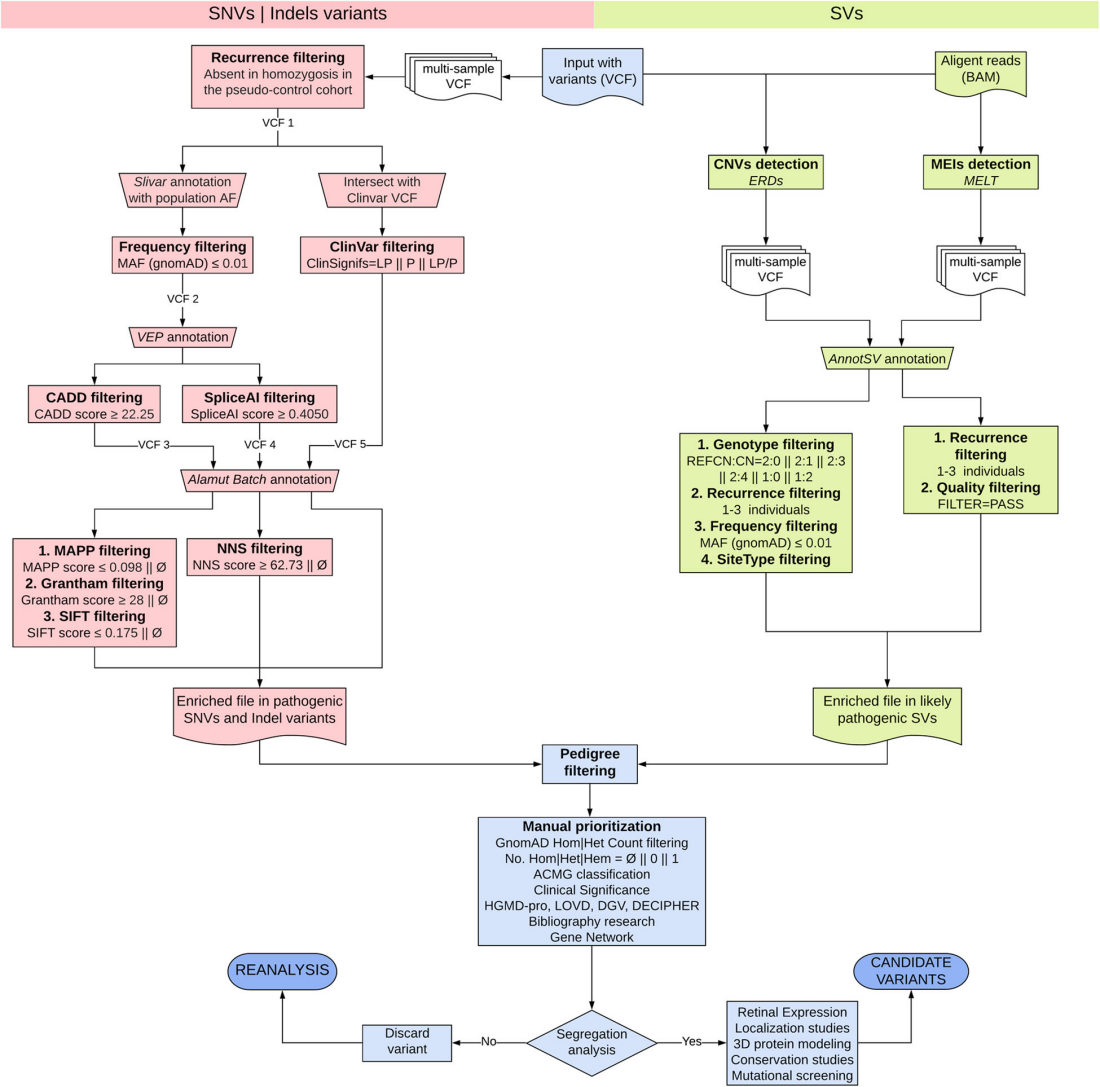
Discovery pipeline for WGS-data analysis. The discovery pipeline consisted of the use of different variant tools (in italic) for the application of several filters (in bold) aiming at the identification of potentially pathogenic variants, and the reduction of the number of neutral variants pending to be assessed. Two different branches, one for the prioritization of SNVs and indels, and another one for SVs, converged into a single file for manual curation. Variants passing filters were then segregated in the family and functional studies were performed when necessary. A reanalysis of the data should be conducted if no candidate variants were identified. The boxes in pink color relate to the analysis of the SNVs and indels variants, whereas the boxes in green color correspond to the analysis of SVs. The boxes in blue color are common steps for both analyses. The version used for each annotation tool were: Alamut**®** Batch v1.11, Slivar v0.2.7, VEP release 104, AnnnotSV 2.2 online, ERDS v1.1. The Ø symbol means without quantitative prediction outcomes. The REFCN: CN refers to the copy number of the variants present in the reference vs. the patient’s genomes, being 2:0 for homozygous deletions; 2:1 for heterozygous deletions, 2:3 for heterozygous duplications, 2:4 for homozygous duplications 1:2 for hemizygous duplications, and 1:0 for hemizygous deletions. CN copy number in the patient’s genome, Hem hemizygous, Het heterozygous, Hom homozygous, LP likely pathogenic, MEIs mobile elements insertions, P pathogenic, REFCN copy number in the reference genome.

The application of the discovery pipeline (Fig. 3) in the IRD validation dataset allowed us to validate the 89.80% (44 out of 49) of the causal variants. The remaining 10.20% (5 out of 49) were filtered out by CADDv1.6 cutoff and consisted of two in-frame variants, two splicing variants in non-canonical positions, and one missense variant (Fig. 2B). Additionally, the discovery pipeline was applied in the dataset from the hereditary cancer cohort and neurological diseases cohort to evaluate its efficacy in these diseases. Regarding the hereditary cancer cohort, the 97.83% (90 out of 92) of the causal variants were validated (Fig. 2C). In the neurological diseases cohort, our algorithm allowed us to recover the 95.65% (44 out of 46) of the causal variants (Fig. 2D). The nature of the variants that integrate each validation dataset can influence the validation ratios, being the highest for the hereditary cancer dataset, which is composed of 70, 66% of loss-of-function variants (stop gain, frameshift, and canonical splicing), in contrast to the 44.9% of loss-of-function variants of the IRD cohort. Furthermore, the highest ratio of causal and non-causal variants was obtained applying the same combination of tools (“CADDv1.6+MAPP + Grantham+SIFT”).

### Application of the discovery pipeline

**Fig. 4:**
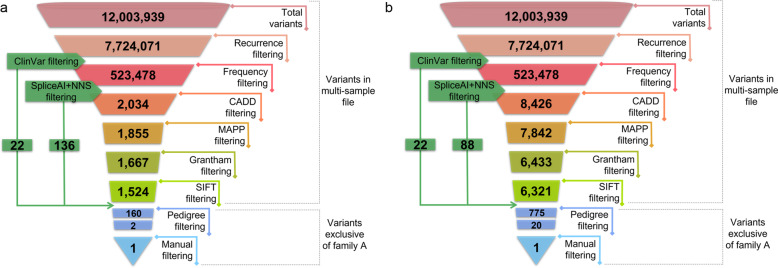
Variants filtering and prioritization scheme using the optimal cutoffs vs. the literature cutoffs. **a** Number of SNVs remaining after applying the optimized cutoffs. **b** Number of SNVs remaining after applying the general cutoffs described in the literature. As the starting point for the application of the first filters, a unique multi-sample file containing the WGS data from 14 individuals (discovery cohort) was used. To rescue those likely pathogenic SNVs that could have been filtered out by applying the general filtering, both ClinVar and “SpliceAI+NNS” filtering steps were applied independently after the recurrence filtering. In this case, the number of SNVs exclusive of family A has been broken down into two boxes. The upper box shows the total number of variants exclusive of family A after removing redundant variants. The lower box refers only to the number of homozygous variants.

The discovery dataset encompassed more than twelve million of SNVs, of which 7,724,071 variants passed the recurrence and multiallelic variants filters. The application of the frequency filtering revealed 523,478 variants, of which 1524 variants passed “CADDv1.6 + MAPP + Grantham+SIFT” filter (Fig. 4A).

The pedigree filtering applied below is exclusive of each family, so the number of variants pending to be manually evaluated varies according to the initially assumed mode of inheritance and the genotype/phenotype of the sequenced individuals as a first approach (Table 2). In simplex families, variants consistent with autosomal recessive, autosomal dominant, and X-linked traits have been considered. In consanguineous families, variants that were homozygous in affected patients but not in their unaffected relatives were first prioritized, followed by the compound heterozygous variants.

**Table 2:** Variants prioritized using the WGS pipeline in the RP families of the discovery cohort.

Family n, Seq. Indiv.	Inh.	Cons.	%Q30	Cov.	Gene	cDNA	Protein	Index GT	ACMG
Family A	AR	Yes	85.35	34.85x	*CFAP20*	NM_013242.3:c.337 C > T	p.Arg113Trp	Hom	VUS/LP
*n* = 2, Index & 1 unaff.					*FAHD2A*	NM_016044.3:c.328 T > C	p.Cys110Arg	Hom	VUS
					*IGHMBP2*^a^	NM_002180.3:c.1130 G > A	p.Cys377Tyr	Het	VUS/LP
					*IGHMBP2*^a^	NM_002180.3:c.1422 C > A	p.Asp474Glu	Het	VUS/LP
Family B	S	No	84.25	33x	*ANKS1B*	NM_001352186.1:c.2740 G > T	p.Asp914Tyr	Het	VUS/LP
*n* = 2, Index & 1 unaff					*ASB1*	NM_001040445.3:c.67 T > G	p.Trp23Gly	Het	VUS/LP
					*ATP2A1*^a^	NM_173201.4:c.1015 G > A	p.Val339Ile	Het	VUS/LP
					*CD163L1*	NM_001297650.1:c.1262 G > A	p.Gly421Glu	Het	VUS
					*COL24A1*	NM_152890.7:c.3673 G > A	p.Gly1225Arg	Het	VUS/LP
					*FAM86B2*	NM_001137610.2:c.347 C > G	p.Ser116Ter	Het	VUS/B
					*FCER2*	NM_001220500.2:c.316 + 70 G > T	p.?	Het	VUS
					*FOXC1*^a^	NM_001453.3:c.-429C > G	p.?	Het	VUS/LP
					*MAP2K7*	NM_001297555.1:c.808 C > T	p.Arg270Trp	Het	VUS/LP
					*MS4A4A*	NM_148975.3:c.331-1365 C > G	p.?	Het	VUS
					*NKX2-8*	NM_014360.4:c.716 G > A	p.Trp239Ter	Het	LP
					*NLRP6*	NM_138329.2:c.1874T > C	p.Leu625Pro	Het	VUS/LP
					*NPIPA1*	NM_006985.4:c.514 A > T	p.Lys172Ter	Het	VUS/P
					*POGLUT1*^a^	NM_152305.3:c.699 T > G	p.Asp233Glu	Het	P
					*SCIN*	NM_001112706.3:c.1286 A > G	p.Tyr429Cys	Het	VUS/LP
					*SLC34A2*^a^	NM_006424.3:c.1565 C > G	p.Ser522Cys	Het	VUS
					*TLCD5*	NM_001198670.2:c.740 C > G	p.Ala247Gly	Het	VUS
					*TRIB3*	NM_001301201.1:c.349 A > G	p.Thr117Ala	Het	VUS/LP
					*TSHZ1*^a^	NM_001308210.2:c.40 + 6343 T > A	p.?	Het	VUS
					*XPC*^a^	NM_004628.5:c.1599 G > C	p.Glu533Asp	Het	VUS/LP
Family C	S	No	84.8	34.5x	*ATN1*^a^	NM_001007026.2:c.3001 G > A	p.Glu1001Lys	Het	VUS/LP
*n* = 2, Index & 1 unaff.					*CHD6*	NM_032221.5:c.3497 A > G	p.Gln1166Arg	Het	VUS
					*GPATCH11*	NM_174931.4:c.100 C > T	p.Arg34Ter	Hom	P
					*NTNG1*	NM_001113226.3:c.712 C > T	p.Arg238Cys	Het	VUS/LP
					*ODF1*	NM_024410.4:c.643 T > A	p.Cys215Ser	Hom	VUS
					*OR5AC2*	NM_054106.1:c.128 G > A	p.Gly43Asp	Het	VUS
					*PAK5*	NM_020341.4:c.-12 + 22185 C > T	p.?	Hom	LB
					*PWP2*	NM_005049.3:c.1318 C > T	p.Arg440Ter	Het	P
					*SDHA*^a^	NM_004168.4:c.1552-2472 C > T	p.?	Het	VUS/B
					*SLCO2A1*^a^	NM_005630.3:c.582 T > A	p.Tyr194Ter	Het	P
					*TRIML1*	NM_178556.5:c.409 G > T	p.Glu137Ter	Het	VUS/LP
					*WNK1*^a^	NM_213655.4:c.3867 + 438 A > G	p.?	Het	VUS/B
Family D	AR	Yes	86.15	35.3x	*MAP4K3*	NM_003618.4:c.598 G > T	p.Val200Leu	Hom	LB
*n* = 2, Index & 1 unaff.					*PKD2L1*	NM_016112.3:c.649 C > T	p.Arg217Trp	Het	LB
					*PKD2L1*	NM_016112.3:c.235 + 1 G > A	r.spl	Het	VUS
Family E	AR	No	88.2	35.6x	*TAS1R1*	NM_138697.4:c.269 C > T	p.Thr90Met	Het	LB
*n* = 1, Index					*TAS1R1*	NM_138697.4:c.2070del	p.Gln690HisfsTer6	Het	LB
Family F *n* = 2, Index & 1 aff.	AR	Yes	86.8	35.2x	*PCDHA1*	NM_018900.4:c.1049 T > G	p.Leu350Arg	Hom	VUS/LP
Family G	AD	No	85.1	35.7x	*C9orf24*	NM_032596.4:c.703-174 G > T	p.?	Het	VUS
*n* = 3, Index, 1 aff & 1 unaff.					*CREB3*	NM_006368.5:c.825 C > A	p.Tyr275Ter	Het	P
					*IPPK*	NM_022755.6:c.1111 C > T	p.Gln371Ter	Het	P

The columns % Q30 and Cov. reflect the average quality values of the sequenced individuals of the same family.

AD autosomal dominant, aff affected, AR autosomal recessive, Cons consanguinity, Cov coverage, GT genotype, Hem hemizygous, Het heterozygous, Hom homozygous, Inh Inheritance, LB likely benign, LP likely pathogenic, n number of sequenced individuals, P pathogenic, S simplex, Seq. Indiv. sequenced individuals, Unaff unaffected.

^a^Gene associated with an OMIM phenotype (Further details in Supplementary Table 3).

This approach resulted in the identification of 45 rare SNVs prioritized in the seven RP families of the discovery cohort (~6 variants per family), all of them were absent in homozygous status in unrelated controls (0 homozygous in gnomAD database). According to ACMG^15^ criteria, these variants were classified as pathogenic (*n* = 6), likely pathogenic (*n* = 1), variants of uncertain significance (*n* = 33), and likely benign variants (*n* = 5), which were located in 42 different genes (Table 2). Eleven out of these genes have been previously associated to a human phenotype according to OMIM database (accessed in November 2021) (Supplementary Table 3). Of note, the *RPGR* orf15 region was manually inspected in the 14 patients of the discovery cohort due to its difficulty to sequence. We tested the coverage of this region, resulting in a mean coverage of 10.53x in men and 20.87x in women within the most complex interval (chrX:38144794-38146346; GRCh37) (Supplementary Fig. 2). Non-causal variants were detected here.

The number of variants remaining after the application of each filtering step in family A is depicted in Fig. 4. The pedigree filter further reduced the number of candidate pathogenic variants to 160, including ClinVar pathogenic variants and variants passing “SpliceAI+NNS” thresholds.

As family A was consanguineous, two homozygous variants were firstly prioritized, one in the *CFAP20* gene (c.337 C > T; p.Arg113Trp), and the other in the *FAHD2A* gene (c.328 T > C; p.Cys110Arg); none of which have been previously associated with a human phenotype in OMIM database. It should be noted that, when the threshold values previously described in the literature were used (Supplementary Table 4), the number of variants in each step was greater, being up to 90% more for manual curation (from 2 to 20) (Fig. 4B).

**Fig. 5:**
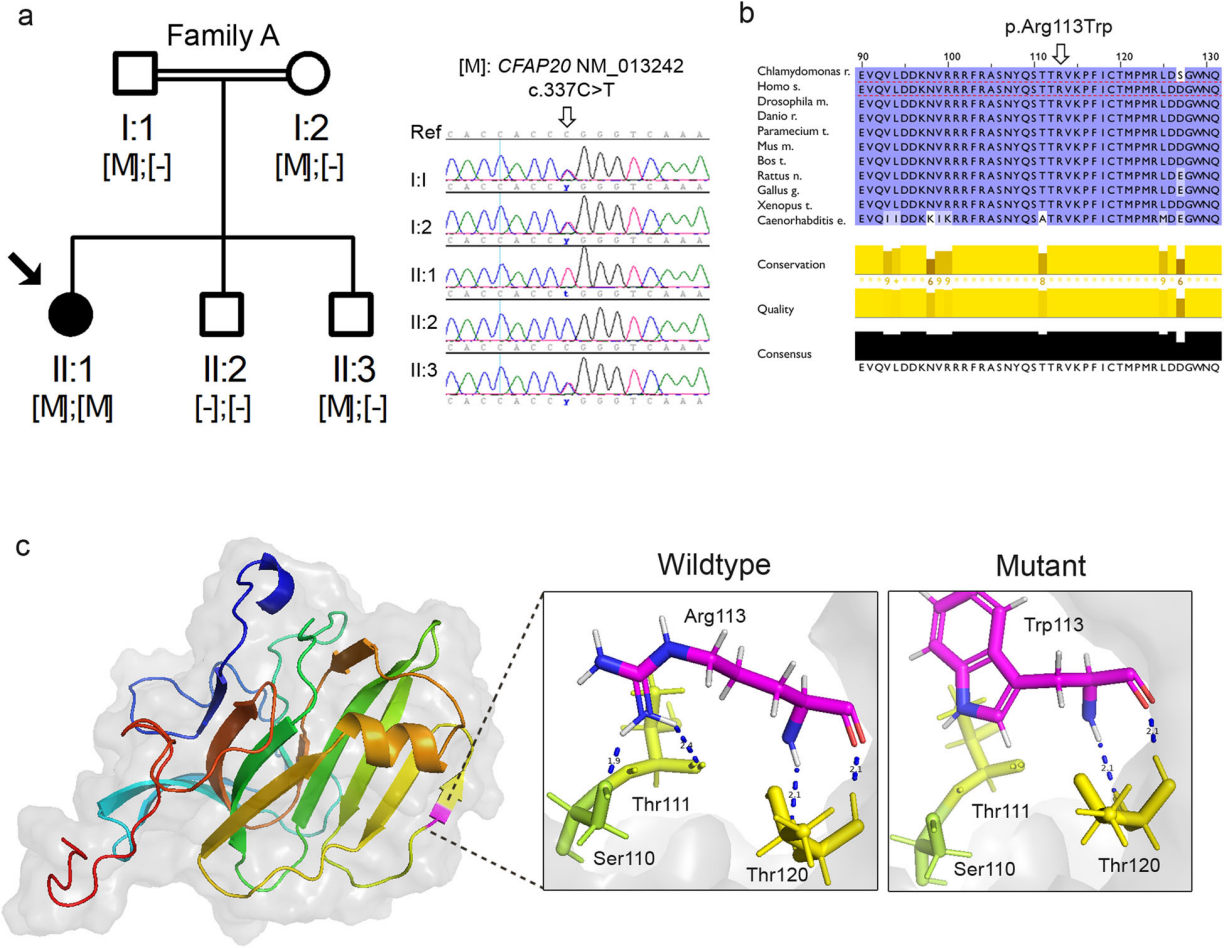
Segregation studies and in silico pathogenicity assessment of the candidate variants identified in *CFAP20*. **a** Pedigree of family A with the segregation analysis of *CFAP20* mutation (NM_013242; [M] = c.337 C > T; p.Arg113Trp). Whole-genome sequenced individuals are marked with an asterisk. Below, the genotypes of each individual are displayed (left panel). Electropherogram depiction of family A individuals confirming the co-segregation of the variant with the disease (right panel). **b** Visualization of the T-Coffee alignment of 11 CFAP20 orthologs using the Jalview program. The conservation annotation histogram (below) shows conservation of the physicochemical properties: an asterisk ‘*’ indicates absolutely conserved residues (score 11), a plus symbol ‘+’ marks columns where physicochemical properties are conserved (score 10); less conserved positions are shown in darker colors with decreasing score. Quality of the alignment based on BLOSUM62, and an alignment consensus row are also shown. Positions are colored white to blue according to increasing sequence identity (BLOSUM62 punctuation). **c** Three-dimensional modeling showing a cartoon view of human CFAP20 protein. The mutated residue (pink) is in a β-strand secondary structure, depicted as an arrow (left panel). A detailed view of wild-type Arg113 vs mutant Trp113 and its interacting amino acids (Ser110, Thr111, and Thr120) (right panel). Hydrogen bonds are shown as blue dashed lines with the donor-acceptor distances depicted in Å.

During the manual prioritization, *CFAP20* was selected for further analysis, since it is a ciliary gene^16–18^ that interacts with a known RP gene (RPGeNet^19^). Besides, the function and mutational data reported in the literature^20,21^ stronger supported the prioritization of *CFAP20* over *FAHD2A*, which was discarded based on its poor functional and mutational bibliographic support, its lack of interaction with other known RP genes, and the milder effect of the variant according to the ACMG^15^ criteria (Table 2). Sanger sequencing confirmed segregation of the *CFAP20* variant with the RP in the five members of Family A (Fig. 5A). Remarkably, up to now, this variant has been detected only in heterozygous state in 5 out of 165,392 unrelated controls (MAF = 0.0000121) from different public allele frequency databases such as gnomAD, EVS, Bravo, 1000 g, and CSVS^22^, which collects genomic data from Spanish-local population. Moreover, we investigated how tolerated were variants in the *CFAP20* gene in the base of the gnomAD constraint metric LOEUF. The statistical performance denoted outstanding discrimination by the LOUEF score, reflected in the high AUC value obtained (AUC = 0.932) in the ROC curve analysis. The LOUEF score for the *CFAP20* gene is 1.008 which is under our established cutoff (≤1.455) (Supplementary Fig. 3).

The manual prioritization in the rest of the families (Families B–G) is resulting in a number of prioritized variants and genes (Table 2). However, further expression, localization, segregation, and interaction studies are needed to evaluate the role of these variants in the etiopathogenesis of the RP in these families.

Regarding the SVs analysis, after applying the pedigree and manual filters, no variants consistent with the disease were identified in the discovery cohort.

### Protein structural analysis, expression assays, localization studies, and mutational screening of *CFAP20*

To evaluate evolutionary conserved positions in CFAP20, we performed the alignment of 11 CFAP20 orthologous sequences using Jalview. The strong evolutionary conservation of the CFAP20 protein and the complete physicochemical conservation of the mutated residue Arg113 is shown in Fig. 5B.

Furthermore, three-dimensional modeling for CFAP20 using PyMOL Molecular Graphics System showed that Arg113, a positively charged amino acid, interacts with three other amino acids through hydrogen bonding (Fig. 5C). Specifically, Arg113 forms one hydrogen bond with Ser110 and Thr111, and two with Thr120. *In silico* mutagenesis at position 113 to tryptophan, a non-polar aromatic amino acid, predicted loss of two hydrogen bonding interaction points, (Ser110, and Thr111).

**Fig. 6:**
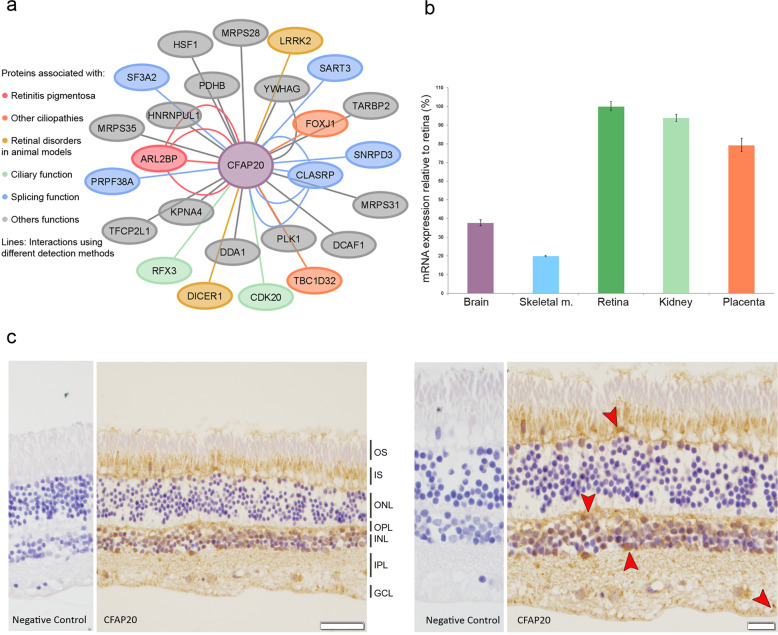
Analysis of CFAP20 interaction network, and expression profiles. **a** Protein-protein interaction (PPI) network analysis of CFAP20 showing common interactions between BioGRID (3.5) and IntAct databases. The PPI map was drawn using Cytoscape v3.8.023. Different colors were employed to mark the interactors with a role in the etiopathogenesis of IRDs and other related disorders, using information from different functional databases (OMIM, Uniprot, etc). Each line represents a PPI identified by a different detection method including validated two hybrid, socioaffinity inference, or coimmunoprecipitation. **b** Relative expression levels of *CFAP20* in commercial cDNA derived from five different human tissues. Depicted is the relative amount of mRNA in retina tissue vs. the other tissues, normalized to the expression of the housekeeping gene *GAPDH*. All the samples were executed in triplicates. Error bars show SD. **c** Immunohistochemical analysis of CFAP20, using rabbit polyclonal anti-GTL3 antibody (ab225952; alias symbol of *CFAP20*), on paraffin-embedded sections of human eye of unaffected donors. Magnification: 40x (left) and 60x (right). Scale bars: 50 µm (left) and 20 µm (right). Immunostaining of the tissue sections showed strong positive staining (brown) of CFAP20 in the inner segment of the photoreceptors, followed by the outer plexiform layer, the nucleus of the cells of the inner nuclear layer, and the nucleus of the ganglion cells (arrows). GCL ganglion cell layer, INL inner nuclear layer, IPL inner plexiform layer, IS inner segment, ONL outer nuclear layer, OPL outer plexiform layer, OS outer segment.

In addition, the protein-protein interaction studies revealed a network, comprised of 25 CFAP20-connected proteins, some of which are involved in ciliary function or forming part of the spliceosome (Fig. 6A). Remarkably, CFAP20 interacts with disease-causing proteins including: (i) ARL2BP, associated with RP, (ii) TBC1D32 and FOXJ1, related with ciliopathies, and (iii) LRRK2 and DICER1, involved in retinal degeneration in animal models.

In order to study the expression of *CFAP20* in different human tissues, we used real-time PCR and ready-to-use cDNA from retina, brain, placenta, kidney, and skeletal muscle. As a result, we found that the expression level of *CFAP20* mRNA was the highest in adult retina, followed by kidney and placenta (Fig. 6B).

The tissue distribution of human CFAP20 was also investigated by immunohistochemistry using human retina sections from unaffected individuals. Specific immunolabeling using the CFAP20 antibodies was observed, from the stronger to the weaker staining, in the inner segment of the photoreceptor cells, the outer plexiform layer, the nucleus of the cells of the inner nuclear layer, and in the ganglion cells layer (Fig. 6C).

Amplicon NGS sequencing of all coding exons and its intronic flanking regions of *CFAP20* revealed no variants consistent with the disease among the 264 additional IRD unsolved cases analyzed.

### Clinical findings in the family A

**Fig. 7:**
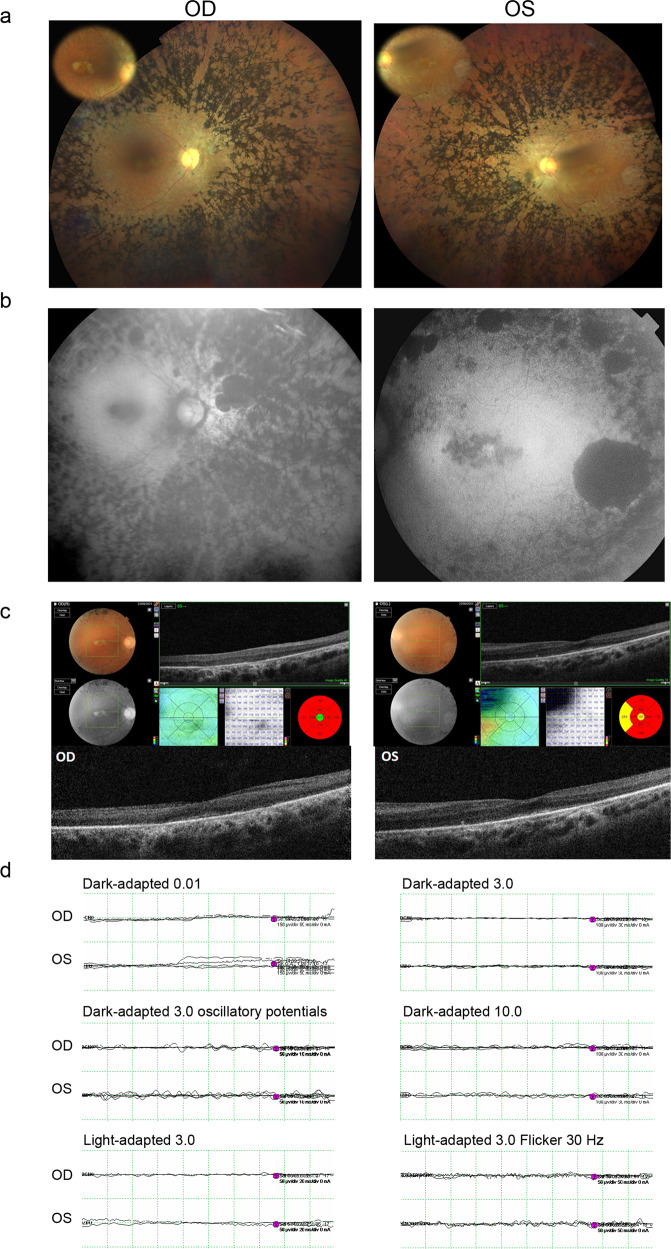
Ophthalmic characterization of the right (OD) and left (OS) eye of the 43-year-old female with RP from the family A. **a** Color fundus photographs showing widespread bone spicule pigmentation, arteriolar narrowing, and atrophic patches in the fovea. **b** Fundus autofluorescence imaging showing hypoautofluorescent lesions in the macula corresponding to retinal pigment epithelium atrophy. **c** Optical coherence tomography of the right (OD) and left (OS) eyes showing generalized atrophy of the photoreceptor cells layer. **d** Full-field electroretinogram of both eyes showing extinguished scotopic (dark-adapted) and photopic (light-adapted) responses bilaterally.

The family A proband, a 43-year-old female, is the first child of first-degree cousin parents with two other unaffected siblings. The patient displayed progressive night blindness with photophobia since age 17 and impaired color vision, poor visual acuity (left eye, 20/100; right eye, 20/63), and concentric narrowing of visual field, at diagnosis. The recent fundoscopic study, and the fundus autofluorescence imaging, were consistent with a clinical diagnosis of typical RP characterized by bone spicule pigmentation, narrowed retinal vessels, loss of the retinal pigment epithelium, and atrophic patches in macula (Fig. 7 A and B). OCT imaging revealed generalized atrophy of the photoreceptor cells layer but relatively preserved in central macula (Fig. 7C). Full-field electroretinography (ERG) revealed completely bilateral extinguished scotopic and photopic responses (Fig. 7D). The abolished ERG responses, the RPE degeneration, and the diminished visual acuity (best-corrected visual acuity of 0.2 in both eyes) indicated an advanced disease. Additional findings included posterior capsular opacification. The patient did not display systemic symptoms consistent with a syndromic phenotype. Other unrelated pathologies present in the index patient were subclinical hypothyroidism and beta-thalassemia.

## Discussion

To date, targeted sequencing, such as gene-panel sequencing and WES, are the NGS approaches more frequently used in the clinical setting. However, the recent advances in WGS have enabled wider use of this technology, even leading to its gradual incorporation in some health systems^9^. Currently, we consider that the cost-benefit balance regarding data quality, analytical efforts, and diagnostic rate indicates that panel-based sequencing is still the most efficient first NGS strategy for the detection of disease-causative genetic variants in IRD, at least in the context of the diagnostic routine of public hospitals^23^. However, around 40% of cases remain unsolved after this application, which would be eligible for larger-scale techniques as WGS. Thus, these extended strategies would be applied only as a second step and would not replace panel sequencing. Nevertheless, WGS is starting to emerge as an efficient first-level test^24^, thanks to its ability to screen for both deep-intronic regions and variants in novel genes, and its greater uniformity of coverage allows better detection of structural variants. Before proceeding to the identification of variants in novel genes, it may be helpful to discard the presence of any pathogenic allele types in genes already involved in the disease, only in this way, the level of uncertainty associated with the causality of a variant in a new candidate gene would be reduced. However, one of the most important barrier to implementing WGS in the clinical practice is data management and storage^25^. The lack of systematized protocols to filter and prioritize causative variants in WGS data, prompted us to develop an effective approach to be used as a standardized workflow for the identification of disease-relevant variants in novel candidate genes for IRD.

Deleteriousness prediction methods are instrumental for variant effect interpretation helping to prioritize large amounts of data generated by sequencing projects. This study provides a comprehensive analysis of which predictor tool, or combination of them, is best suited for discovery applications, as well as which are the most reliable cutoffs regardless of those reported in the literature. In this regard, although CADDv1.6 prediction showed the highest performance, probably because it is an ensembled method that provides scores for all types of variants^26^, the filtered FP rate was still very elevated. The combination of this method with the predictors MAPP, Grantham, and SIFT enabled us to further reduce the number of neutral variants. Additionally, the use of our customized cutoffs, instead of the published thresholds, allowed us to significantly reduce the number of variants on the common VCF file, resulting in an increased effectiveness by reducing the number of variants for manual filtering. Of note, although this pipeline could be used for the analysis of both, WES and panel data, it is specially designed for WGS data, since not all annotation tools work well with large sequencing experiments^27^.

Our results demonstrated the importance of integrating different prediction tools in a standardized pipeline and applying filters validated and optimized using local carefully curated datasets. In fact, previous work highlighted the need for a detailed catalog of local variability since there are relevant differences in allelic frequencies of both polymorphic and pathogenic variants between populations^28^. For this reason, working with local datasets is crucial for an accurate establishment of the clinical significance of candidate variants. Although other authors^26,29^ have performed multiple comparisons among prediction methods, the input data was taken from public databases which may not be properly curated or be deficient in local data, leading to the misclassification of variants and limiting the accuracy of the resulting performance estimations^26,29^. In addition, unlike other studies in which variants with high MAF composed the neutral dataset^29,30^, our group of benign variants was previously filtered by MAF letting us test how well a predictor performs when the benign variants have the same allele frequency that known pathogenic variants. This fact approaches our study to a real filtering scenario being able to establish a more precise fixed threshold. The favorable results obtained using heterogeneous validation cohorts demonstrated that our optimized pipeline could be applied to the analysis of NGS data from individuals with other genetic disorders, not only for IRDs patients. Hence, the implemented translational strategy allows an accurate prioritization and assessment of NGS data in the clinical setting, which is essential to establish personalized medicine.

Remarkably, the application of our pipeline to the discovery cohort allowed the identification of one homozygous variant (c.337 C > T; p.Arg113Trp) in the candidate gene *CFAP20* as the most likely cause of non-syndromic RP in one of the families. Previous studies, involving unicellular^16,31^, and multicellular organisms^18^, showed that Bug22 (ortholog name of the cilia and flagella associated protein 20, *CFAP20*) plays a critical role in cilia and flagella formation and morphogenesis. Bug22 depletion causes defects in ciliary and flagellar morphology and motility in Paramecium^16^, Chlamydomonas^17^, and Drosophila^18^ (Supplementary Table 5). Of note, knockdown experiments in Zebrafish^17^ revealed a phenotype consistent with ciliary dysfunction^32^ including a curved body axis, short somite length, and defective heart-looping orientation. In addition, CFAP20 has also been detected in the primary cilium-derived photosensory rod outer segments of mouse retina^33^. These results implied that *CFAP20* may be also important for assembly or stability of cilia in vertebrates^17^. Moreover, depletion of CFAP20 in human hTERT-RPE1 cells resulted in the appearance of longer cilia, and reduced axonemal polyglutamylation^18^, demonstrating the implication of *CFAP20* in the regulation of post-translational modifications of the ciliary axoneme in human cells. The fact that almost one-quarter of known photoreceptor degeneration genes are associated with ciliary structure or function^33,34^, along with the high evolutionary conservation of CFAP20, and its low LOEUF score (below our cutoff), support the prioritization *CFAP20* as a candidate gene for autosomal recessive IRD.

Sequencing of more than one individual per family and the application of the recurrence filter has allowed us to refine the number of likely causative homozygous variants, which in consanguineous individuals would be expected to be higher. Our patient, born to consanguineous parents, harbored a homozygous rare missense variant in *CFAP20* (c.337 C > T; p.Arg113Trp), and received a clinical diagnosis consistent with non-syndromic RP. Recently, a conference report described another family with three affected individuals with clinical manifestations partially resembling the phenotype observed in our proband, including RP with an onset in adolescence^21^. These patients harbored two heterozygous *CFAP20* variants, one missense, and one canonical splicing variant, segregating in the family^21^. In addition, the three siblings had a history of learning disabilities in school and motor coordination difficulties, suggesting the implication of *CFAP20* in a syndromic form of RP. As occurs with mutations in ~30 ciliary genes^35^, the manifestation of extra-ocular features can vary from patient to patient^36,37^, depending on the severity of the mutations^36,37^, the genetic background^38^, the presence of genetic modifiers^39^ or tissue-specific alternative splicing^40^, among other factors. Interestingly, depending on the mutation, the same ciliary gene can cause syndromic or non-syndromic retinopathies, thus emphasizing the highly refined specialization of the photoreceptor neurosensory cilia, and raising the possibility of photoreceptor-specific molecular mechanisms^41^.

Further, we observed high *CFAP20* gene expression in the retina compared to other tissues, and localization in the inner segment of photoreceptor cells, suggesting that CFAP20 could have a role in the human retina. Moreover, the molecular modeling of CFAP20 revealed that the p.Arg113 residue may be involved in some interactions with important biological roles. In fact, p.Arg113 was predicted to interact with p.Thr111, one of the seven consensus positions in species that have cilia or centrioles, suggesting a relevant role of this specific residue in the development and function of the cilia or centrioles^16^. These data suggest that the *CFAP20* variant, p.Arg113Trp, could affect protein folding and interaction with the consensus residue p.Thr111.

PPI network analysis of CFAP20 significantly contributed to our understanding of potential relationships between CFAP20 interactors and retinal disease mechanisms. One of the top-ranked interactors of *CFAP20* was *ARL2BP*, a known autosomal recessive RP gene^42^ required for the formation of ciliary doublets of the photoreceptors and for the morphogenesis of its outer segment^43^. We also found other ciliopathy associated partners of *CFAP20*, namely, *TBC1D32*, mutated in patients with oro-facio-digital syndrome type IX^44,45^; *FOXJ1*, implicated in primary ciliary dyskinesia 43^46^; *LRRK2*, a Parkinson disease 8 gene, involved in retinal degeneration by a gain-of-function mechanism in Drosophila^46,47^; and *DICER1*, which deficit induces retinal pigmented epithelium degeneration in a mouse model of age-related macular degeneration^48^. The establishment of a robust interaction network led us to hypothesize that the variant identified in our family might alter some of the interactions with other crucial proteins involved in the etiology of retinal degeneration. However, further functional studies that deepen our understanding of these interactions and their role in disease are needed to test this hypothesis.

Clinically, genotype and phenotype correlations are only now starting to emerge for *CFAP20*, which demands the comprehensive screening of larger patient cohorts to better understand disease pathogenesis in new cases with candidate *CFAP20* variants. Nevertheless, if confirmed, *CFAP20*-associated disease would be clinically variable, ranging from isolated to syndromic RP with a spectrum of neurological defects. The identification and characterization of additional cases will contribute to a better understanding of the factors influencing the variable expressivity of clinical features possibly associated with mutations in this novel candidate gene.

In conclusion, the arrival of the WGS techniques into the clinical practice has aroused great expectations about its potential for identifying the genetic bases of diseases. In this scenario, the development of a translational pipeline for the analysis of WGS data in the clinical setting, based on the reliable use of computational prediction tools, becomes a priority. The use of statistically proven filtering criteria using in-house curated patient genetic data, reinforced the huge diagnostic and discovery capacity of WGS. Our study suggests that the combination of several prediction tools and the use of customized cutoff values improve enormously WGS-data management. Herein, the application of our pipeline has allowed us to identify, in one family, a homozygous variant in *CFAP20*, a potential candidate gene for autosomal recessive RP. Therefore, our study could contribute to expand the mutational landscape of ciliary genes associated to human diseases, reinforcing the importance of this complex organelle as a key player in photoreceptor degeneration.

## Methods

### Subjects and previous NGS studies

The research was conducted in accordance with the tenets of the Declaration of Helsinki (Edinburgh, 2000)^49^, and all experimental protocols were approved by the Institutional Review Board of the University Hospitals Virgen del Rocio and Virgen Macarena (Spain). Written informed consent was obtained from all participants. The genomic DNA of all subjects was isolated from peripheral blood using standard procedures. All affected individuals underwent a thorough ophthalmic evaluation as described elsewhere^50^.

**Fig. 8:**
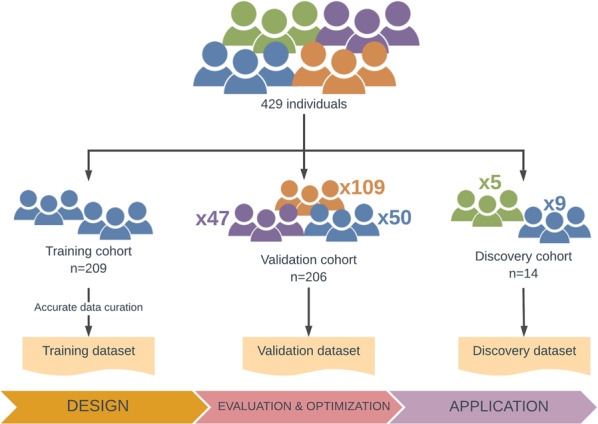
Composition of the cohorts and datasets used in the study. The whole study cohort was composed of 429 subjects including: 268 IRD affected individuals (in blue), 109 hereditary cancer affected individuals (in orange), 47 neurological diseases affected individuals (in purple), and five unaffected relatives of IRD families (in green).

This study involved 429 individuals grouped in three different cohorts: the training cohort (*n* = 209), the validation cohort (*n* = 206), and the discovery cohort (*n* = 14) (Fig. 8). The training cohort comprised 209 IRD patients selected among those who received a genetic diagnosis at the Department of Maternofetal Medicine, Genetics and Reproduction of the University Hospital Virgen del Rocio of Seville in the period from 2016 to 2019 using different NGS targeted approaches^51–53^, among others. The accurate genetic characterization of these patients enabled this group to design and define the prioritization pipeline.

The validation cohort was composed of 206 additional, unrelated patients who also underwent targeted sequencing at our department (unpublished data). This cohort was composed of three sub-cohorts of affected patients from IRD (*n* = 50), hereditary cancer (*n* = 109), and neurological diseases (*n* = 47). The sub-cohort of IRD patients including 33 patients with a genetic diagnosis and 17 patients without a genetic diagnosis to conduct a blind trial, allowing an unbiased evaluation of the parameters proposed with the training dataset. In order to assess if our pipeline could be applied to the analysis of other inherited diseases, the hereditary cancer cohort and the neurological diseases cohort, comprising genetically diagnosed individuals, were employed.

The discovery cohort involved 14 individuals, of which nine were affected and five were unaffected members, belonging to seven unsolved IRD families (Families A–G). WGS was conducted in all the individuals of the discovery cohort, and a comprehensive analysis of the 274 genes previously associated with IRD (RetNet), including coding and non-coding regions, was performed as previously described^54^, but no causal variants were detected in any of these genes. The discovery cohort was employed for the application of the validated workflow in order to achieve their genetic diagnosis and the identification of new disease genes. Interestingly, to facilitate the filtering and prioritization of variants in novel genes, the unaffected individuals of the rest of the families were used as pseudo-controls of the family in the study.

Additionally, 264 unsolved IRD individuals from our cohort were collected in order to conduct the mutational screening of the novel candidate genes.

The genomic data of the individuals belonging to the three cohorts were combined using the VCF sort tool^55^ and the VCF combine tool^56^. The multi-sample VCF files comprised the study datasets (Fig. 8) enabling the application of the pipeline in a more efficient way.

### Curation of the training dataset

The training dataset composed of SNVs affecting IRD genes was first filtered by MAF ( ≤ 0.01) and by the number of homozygous individuals in GnomAD (0, 1). The resulting variants were then classified according to ACMG^15^, using VarSome^57^ v10.1 as a support, and their clinical association in multiple databases (ClinVar, LOVD, HGMD professional, and the literature review). This categorization allowed us to differentiate two groups of variants: (i) Pathogenic and likely pathogenic; and (ii) Benign and likely benign.

The statistical analysis of the splicing predictors was conducted using a subgroup of variants affecting intronic positions ±10 and the first/last codon of the exons. This subgroup was similarly classified as: (i) Pathogenic and likely pathogenic; and (ii) Benign and likely benign attending to the same criteria mentioned above.

Those changes that were not clearly classified in these categories (Variants of Unknown Significance) were discarded for the statistical analysis.

### Predictive tools tested in this study

To obtain the prediction scores used in the statistical analysis, the training dataset was annotated using Alamut® Batch v1.11 software (Interactive Biosoftware), Bystro Genomics^27^, and Ensembl Variant Effect Predictor (VEP, web interface release 104)^58^ (Supplementary Table 4).

Alamut® Batch is based on efficient external prediction tools reporting update information, of which we used the deleteriousness prediction scores for Sorting Intolerant From Tolerant^59^ (SIFT), Grantham^60^, PhastCons^61^, PhyloP^62^, Multivariate Analysis of Protein Polymorphism^63^ (MAPP), Splicing Predictions in Consensus Elements^64^ (SPiCE), Splice Site Finder-like^65^ (SSF), MaxEntScan^66^ (MaxEnt), and NNSplice^67^ (NNS). Bystro Genomics provides three prediction methods: PhastCons-100way, PhyloP-100way, and CADDv1.3. Since the CADD version provided by Bystro is only defined for single-nucleotide variants, a more recent version of CADD (GRCh37-v1.6) was also tested, which was obtained from VEP annotation. This variant annotator gives also the SpliceAI^68^ prediction allowing its assessment. Therefore, two different versions of PhastCons, PhyloP, and CADD were evaluated independently to assess the most efficient method.

To compare the performance of the quantitative score of these prediction methods, SIFT and MAPP scores given by Alamut® Batch were converted, so that a higher score indicates a higher risk of deleteriousness. Similarly, scores of splicing tools SSF, MaxEnt and NNS were converted into the percent variation between the scores for the wild-type sequence and variant sequences. Among the four different delta scores (DS) provided by SpliceAI, the maximum score was used (Supplementary Table 4).

In addition, motivated by the fact that genes that are crucial for the function of an organism will be depleted of loss-of-function variants in natural populations, whereas non-essential genes will tolerate their accumulation^69^, we evaluated the tolerance to inactivation of the novel candidate genes using the constraint metrics from gnomAD. Among them, the LOEUF Score (“loss-of-function observed/expected upper bound fraction”) was used for its good performance to improve molecular diagnosis and advance in the understanding of disease mechanisms^70^.

### Comparison of the predictive tools

To calculate potential cutoff values with a certain degree of sensitivity and specificity for each of the predictive tools, we conducted receiver operating characteristic (ROC) curves using the prediction scores of the training dataset and the ROC curve toolbox of SigmaPlot v14 (Systat Software, Inc). Resulting data were used to establish the optimal cutoff for each prediction method by using three different approaches: Youden’s index^71^, the cutoff value in which sensitivity is equivalent to specificity^72^, and the cutoff value in which sensitivity is 90%.

The area under the ROC curve (AUC) was used to compare the prediction tools, considering a value <0.5 as the result of chance and statistical randomness^73^, and a value close to 1 as a sign of utility of the predictor. The DeLong et al. method^74^ was used for the calculation of AUC since our data type was paired. Sensitivity, specificity, and AUC values were computed with a confidence level of 95%. Due to the existence of missing values for the different prediction methods, the pair-wise deletion^75^ was computed to compare ROC areas. The distribution of both categories of variants (pathogenic and benign) along the prediction scores, were also plotted by dot histograms for each predictor (Supplementary Fig. 1), representing the literature cutoffs (Supplementary Table 4) and our selected optimal values (Table 1) as horizontal lines.

Similarly, a ROC curve analysis was conducted to compare the LOEUF Scores from 207 known autosomal recessive IRD (arIRD) genes (https://sph.uth.edu/retnet/) with the LOEUF Scores from 374 olfactory receptor genes as relatively unconstrained genes. Low LOEUF scores indicate strong selection against predicted loss-of-function (pLoF) variation in a given gene, while high LOEUF scores suggest a relatively higher tolerance to inactivation. The LOEUF cutoff in which sensitivity is 90% was obtained (Supplementary Fig. 3).

In order to ascertain which was the optimal combination of predictors that allowed preserving a high True-Positive (TP) rate, reducing the False-Positive (FP) rate, a combinatorial analysis was performed. Based on its ease of subsequent application, a total of 109 combinations of different predictors, divided into three groups, were analyzed as shown in Supplementary Table 2. We conducted bubble plots to visually inspect the data. To select the most appropriate models, the following ad hoc criteria were established: TP rate ≥85%, FP rate ≤35%, and missing values rate ≤30%. If the model met the criteria, we prioritized a lower FP rate.

Finally, the selected combinatorial models were applied in the IRD validation dataset to determine the most optimal filtering steps for our discovery pipeline, according to the percentage of recovered causal and non-causal variants.

### Variants filtering, prioritization, and pathogenicity assessment

The validated combination of predictors was applied to the WGS data from the discovery cohort as part of our optimized discovery pipeline (Fig. 3).

Briefly, for SNVs and indels, the recurrence filtering, consisting of removing homozygous variants in the unaffected individuals (pseudo-controls), and the multiallelic variants filtering were applied using the tool “Filter tabular” from open source, web-based platform Galaxy^76^ (VCF 1). On the one hand, the VCF 1 file was annotated with the population allele frequency from gnomAD database using the Slivar v0.2.7 software^77^ and, then, the frequency filtering (MAF ≤ 0.01) was applied. The resulting VCF file (VCF 2) was annotated in VEP and filtered by the CADD (CADD PHRED ≥ 22.25) and SpliceAI (max. SpliceAI DS ≥ 0.405) separately. Variants passing these filters were used to create a third and fourth VCF files which were also annotated with Alamut® Batch. Then, MAPP filtering (≤0.098 or missing), Grantham filtering (≥28 or missing), and SIFT filtering (≤0.175 or missing) were applied for the VCF 3, and NNS filtering (≥62.73 or missing) was applied for the VCF 4.

On the other hand, the VCF 1 was intersected with Clinvar VCF (October 2021) to recover variants classified as pathogenic or likely pathogenic in ClinVar database (ClinVar filtering) regardless of whether they meet the above-mentioned filtering criteria or not. This set of variants (VCF 5) was also annotated in Alamut® Batch. All these prioritized variants converged into a single file enriched in pathogenic SNVs and indels (Fig. 3).

Regarding the structural variants (SVs), the CNVs calling was performed using the tool Estimation by Read Depth with Single-nucleotide variants v1.1 (ERDS)^78^, which generated as output a VCF file containing all called SVs per individual. Then, we employed the VCF sort tool^55^ and the VCF combine tool^56^ to create a single multi-sample VCF, which was annotated and ranked using the AnnotSV 2.2 online software^79^. CNVs prioritization was done using the subsequent filters: (i) Genotype filtering which considers only homozygous, heterozygous, and hemizygous deletions and duplications excluding complex and multi-allelic CNVs; (ii) Recurrence filtering which limits the co-occurrence of the same CNV in no more than three individuals of our discovery cohort; (iii) Frequency filtering (MAF ≤ 0.01 or absent in gnomAD); and (iv) SiteType filtering consisting of prioritizing events that include exonic bases. In addition, we used the Mobile Element Locator Tool (MELT v2.2.2)^80^ to discover mobile element insertions (Alu, L1, and SVA elements) in the discovery cohort. The resulting call sets were annotated using AnnotSV and filtered according to the quality status and the recurrence between samples.

Remarkably, a single multi-sample file containing the passing filters variants (SNVs, indels, and SVs) of the 14 individuals, belonging to seven IRD families, was the starting point for the application of the pedigree filtering. This filter should be applied considering the specific pedigree of each family. This step was the first filter specific to the family in the study and focused on the analysis of only those variants present in the index patient, taking into account the genotype, and the phenotype, of the additional sequenced family members. In a first approach, we carry out the prioritization of variants considering the mode of inheritance initially assumed and a common genetic cause in all affected individuals of the same family. However, in those families in which this approach did not lead to candidate variants, the data analysis was conducted under other considerations.

Finally, we conducted a manual curation of candidate variants considering: (i) the number of heterozygous, hemizygous and homozygous individuals and constraint metrics of gnomAD; (ii) the results of the application of ACMG classification^15^ rules; (iii) the clinical significance recorded in additional variant databases (HGMD professional, LOVD, ClinGen, DGV^81^ or DECIPHER^82^); and (iv) the reported retinal association regarding gene function, interaction networks (RPGeNet^19^), expression databases, animal models, etc.

Candidate variants were segregated by Sanger sequencing (SNVs), PCR (MEIs) or RT-PCR (CNVs) according to the manufacturer’s protocols (BigDye® Terminator v3.1 Cycle Sequencing Kit, 3730 DNA Analyzer, Applied Biosystems, USA; Qiagen Multiplex PCR Master Mix, and RT2 SYBR Green ROX qPCR Mastermix Qiagen, Hilden, Germany) in additional family members. The primers used are available in Supplementary Table 6. Structural, expression, localization, and mutational screening studies were conducted if needed.

In case no likely candidate variants were detected using this pipeline, a reanalysis of the data, including the screening of both deep-intronic regions of novel genes, and complex rearrangements, are being conducted.

### Protein structural analysis

The multiple sequence alignment was generated by Jalview v2.11.1.0^83^ with the T-Coffee alignment algorithm^84^. Sequences of CFAP20 orthologs were obtained via UniProt^85^ and filtered for reviewed (Swiss-Prot), including A8IU92 (Chlamydomonas reinhardtii), Q9Y6A4 (Homo sapiens), Q9VKV8 (Drosophila melanogaster), Q6PBJ2 (Danio rerio), A0CDD4 (Paramecium tetraurelia), Q8BTU1 (Mus musculus), Q6B857 (Bos taurus), Q499T7 (Rattus norvegicus), Q5ZHP3 (Gallus gallus), Q6GL74 (Xenopus tropicalis) and Q86D25 (Caenorhabditis elegans).

Protein predictive models of human CFAP20 were obtained using I-Tasser^86,87^. Among the predicted structures, the model with the highest C-score was selected. To analyze the impact of mutagenesis on terms of size and hydrogen bonding, PyMOL Molecular Graphics System, v1.8^88^ was used.

The protein-protein interaction (PPI) network was created by integrating Biological General Repository for Interaction Datasets (BioGRID v3.5)^89^ and IntAct databases^90^ at EMBL-EBI. To restrict the number of PPIs to those with higher levels of evidence, we removed the PPIs predicted by spoke expanded co-complexes. Cytoscape v3.8.0^91^ was used to construct and visualize the PPI network which included common interaction pairs in both databases. The function of connected genes was checked in OMIM (https://omim.org/), Uniprot^85^, and the literature.

### Expression and localization studies in the human retina

The expression of the human *CFAP20* gene was evaluated by real-time qPCR using the RT^2^ SYBR Green ROX qPCR MasterMix (Qiagen, Hilden, Germany) in an Applied Biosystems 7500HT instrument (Life Technologies, CA, USA) with ready-to-use cDNA from five different tissues: retina (QUICK-Clone™ Clontech Laboratories, Inc., CA, USA), brain, kidney, placenta and skeletal muscle (Zyagen, CA, USA). The relative expression of *CFAP20* in the mRNA in retina tissue vs. the other tissues was determined using the comparative Ct (2^-ΔΔCt^) method^92^ with *GAPDH* as endogenous control. All the samples were executed in triplicates.

Localization studies of human CFAP20 in retina sections were done by immunohistochemistry. The human retina sections belonged to five unaffected donors from the University Hospital Virgen del Rocio-Institute of Biomedicine of Seville Biobank (Andalusian Public Health System Biobank and ISCIII-Red de Biobancos PT17/0015/0041). For this purpose, four-micrometer-thick tissue sections from paraffin blocks were baked for 20 min at 65 °C. Antigen retrieval was performed with a PT Link instrument (Agilent, CA, USA), using EDTA buffer (97°C, 20 min). Sections were immersed in H_2_O_2_ aqueous solution (Blocking peroxidase reagent, Agilent, CA, USA) for 10 min to exhaust endogenous peroxidase activity and then covered with 1% blocking reagent (Roche, Mannheim, Germany) in PBS, to block nonspecific binding sites. Sections were then incubated with a 1:400 dilution of primary antibody (Abcam, ab225952) for 1 h at room temperature in a humid chamber. Later, horseradish peroxidase polymer conjugated secondary antibodies (Visualization reagent, Agilent, CA, USA) were used for 1 h at room temperature in a humid chamber and 3,3'-diaminobenzidine was applied for 5 min to develop immunoreactivity. Slides were counterstained with hematoxylin and mounted in DPX (BDH Laboratories, Poole, UK). Images of the stained sections were obtained with an Olympus BX61 microscope and the cellSens Dimension software (Olympus, PA, USA).

### Mutational screening of *CFAP20* in additional IRD families

To evaluate the prevalence of *CFAP20* variants in additional IRD families of our cohort, we designed an amplicon NGS-based approach of all coding exons of *CFAP20* and their flanking intronic regions (Supplementary Table 6). For this purpose, 264 additional unsolved IRD patients underwent deep-amplicon sequencing using a Custom rhAmpSeq library Panel (Integrated DNA Technologies, Inc., IA, USA) in the Illumina’s MiSeq instrument (2 × 150bp paired-end). Data analysis was conducted using MiSeq Reporter software (v2.6) without flag duplicates.

### Reporting summary

Further information on research design is available in the Nature Research Reporting Summary linked to this article.

## Supplementary information

Supplementary Materials

Reporting summary

## Data Availability

The authors confirm that the data supporting the findings of this study are available within the article and its supplementary materials. The prioritized variants were submitted to ClinVar database under the accession ID: SCV002061327. The Whole-genome sequencing data are not publicly available due to families enrolled in this study did not provide additional consent to share raw dataset in a public repository. De-identified data or additional specific variant information may be accessible and requested from corresponding authors G.A. (guillermo.antinolo.sspa@juntadeandalucia.es) and S.B. (salud.borrego.sspa@juntadeandalucia.es).
