# Supplementary material for: A comprehensive WGS-based pipeline for the identification of new candidate genes in inherited retinal dystrophies
Source: NPJ Genom Med. 2022 Mar 4;7:17. doi: 10.1038/s41525-022-00286-0 (PMC8897414; doi:10.1038/s41525-022-00286-0)
Supplement: Supplementary file 1 — Supplementary Materials [file 41525_2022_286_MOESM1_ESM.pdf]

Supplementary Information

A comprehensive WGS-based pipeline for the identification of new candidate genes in inherited retinal dystrophies.

INDEX

- **Supplementary Figure 1. Dot histograms for the distribution of pathogenic and benign variants along the scores range predicted by the different tools.** 2
- **Supplementary Figure 2. Overview of the mean coverage per position along the complex RPGR orf 15 region in the 14 individuals of the discovery cohort by WGS.** 3
- **Supplementary Figure 3. Evaluation of the gnomad constraint metric LOEUF as an estimator of pathogenicity comparing autosomal recessive inherited retinal dystrophy (arIRD) genes vs. olfactory receptor (OR) genes.** 4
- **Supplementary Table 1. List of variants comprising the training data classified as pathogenic or benign variants after an accurate curation.** 5
- **Supplementary Table 2. Predictor combination groups tested during the combinatorial analysis.** 46
- **Supplementary Table 3. Genes prioritized with an associated phenotype according to OMIM database identified in the discovery cohort.** 47
- **Supplementary Table 4. Comparative of the different variant annotation tools and the *in-silico* predictors used in this study.** 48
- **Supplementary Table 5. Different CFAP20 in vivo and in vitro knock-out and knock-down models showing the type of inactivation and its phenotypic effect.** 49
- **Supplementary Table 6. Primers used during the family segregation, mutational screening, and expression studies of *CFAP20* gene.** 50
- **Supplementary references** 51

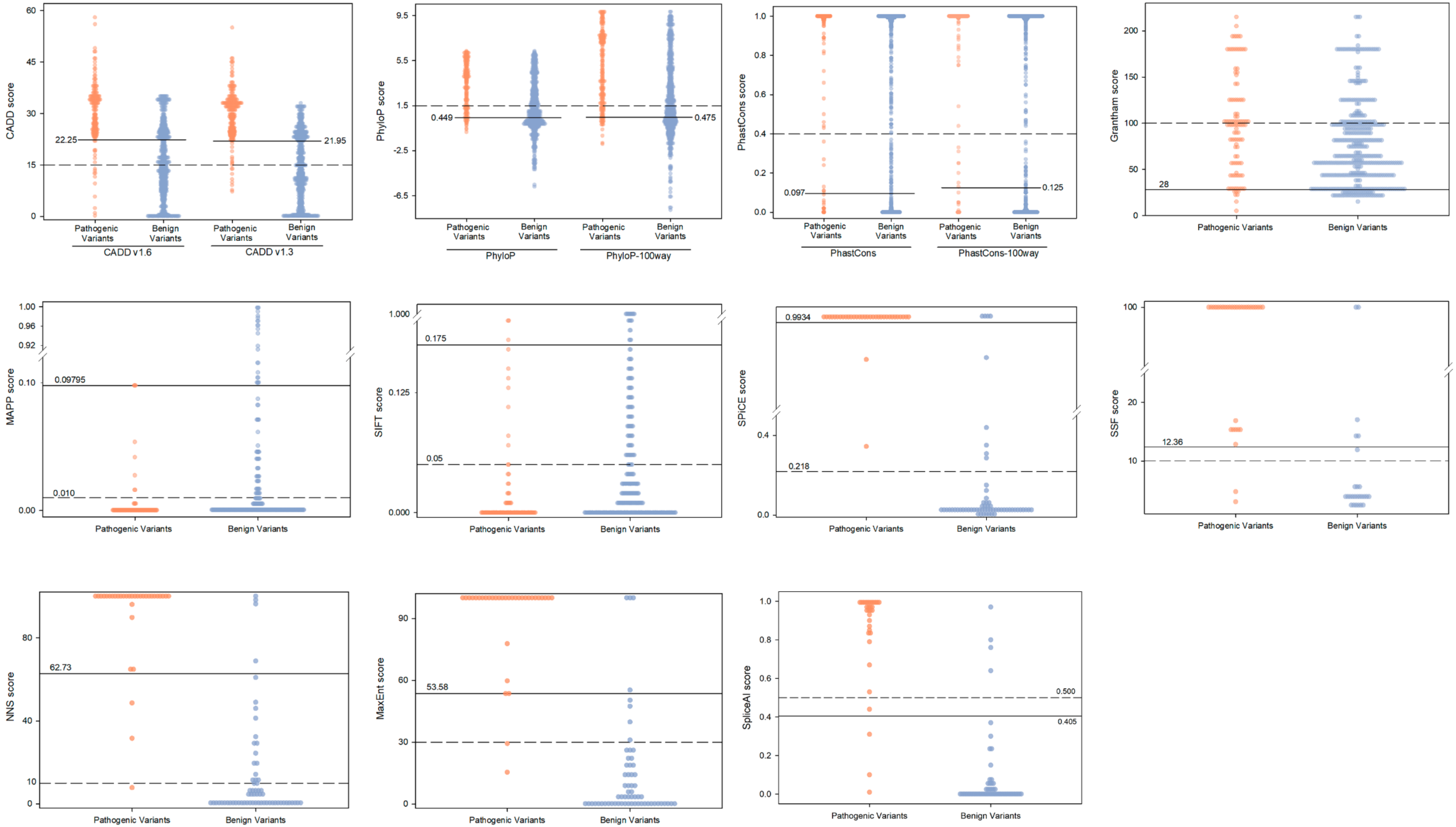

Supplementary Figure 1. Dot histograms for the distribution of pathogenic and benign variants along the scores range predicted by the different tools. Each dot symbolizes a different variant. The optimal cutoff values whose sensitivity is equal to 90% are represented with a continuous line whereas, the cutoff values previously described in the literature are represented with a dashed line.

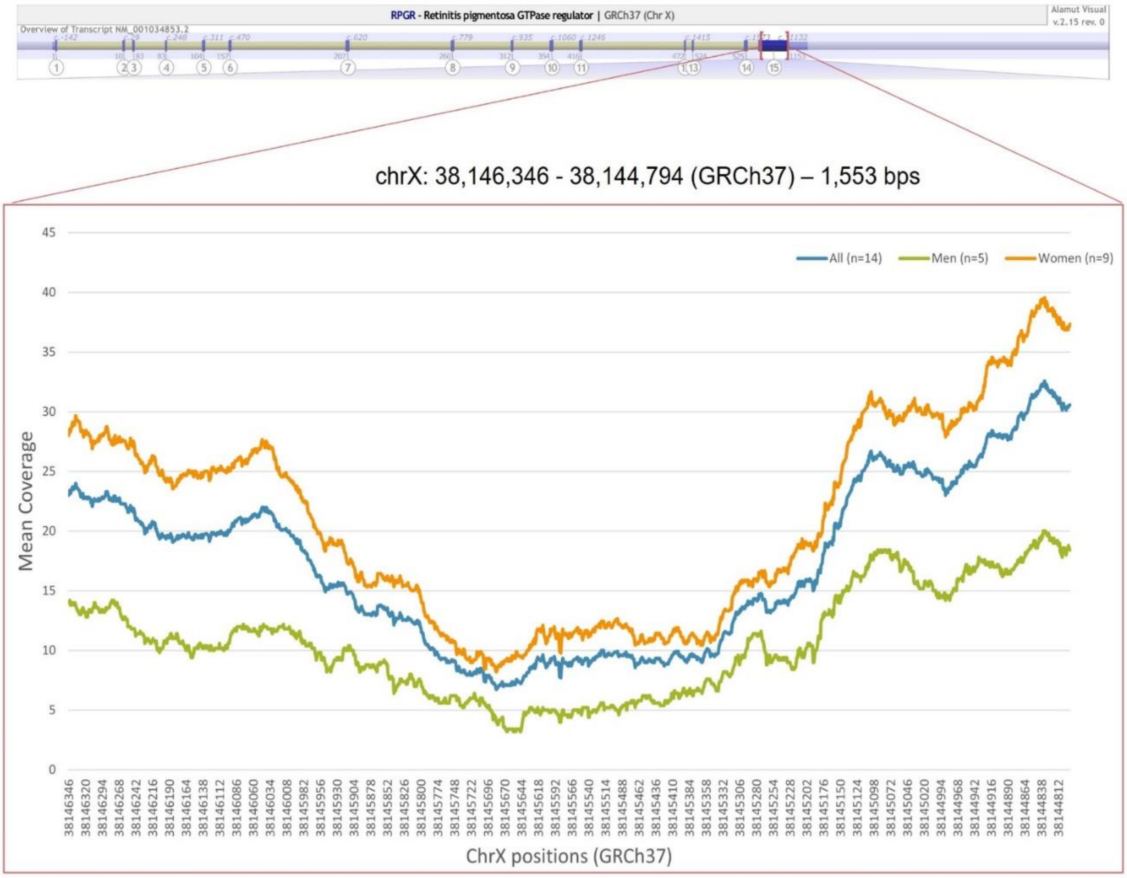

Supplementary Figure 2. Overview of the mean coverage per position along the complex RPGR orf 15 region in the 14 individuals of the discovery cohort by WGS.
An average coverage of 10.53x in men and 20.87x in women was obtained within this interval. Men (n = 5) have been represented in green, women (n = 9) in orange, and the entire discovery cohort (n = 14) has been represented in blue.

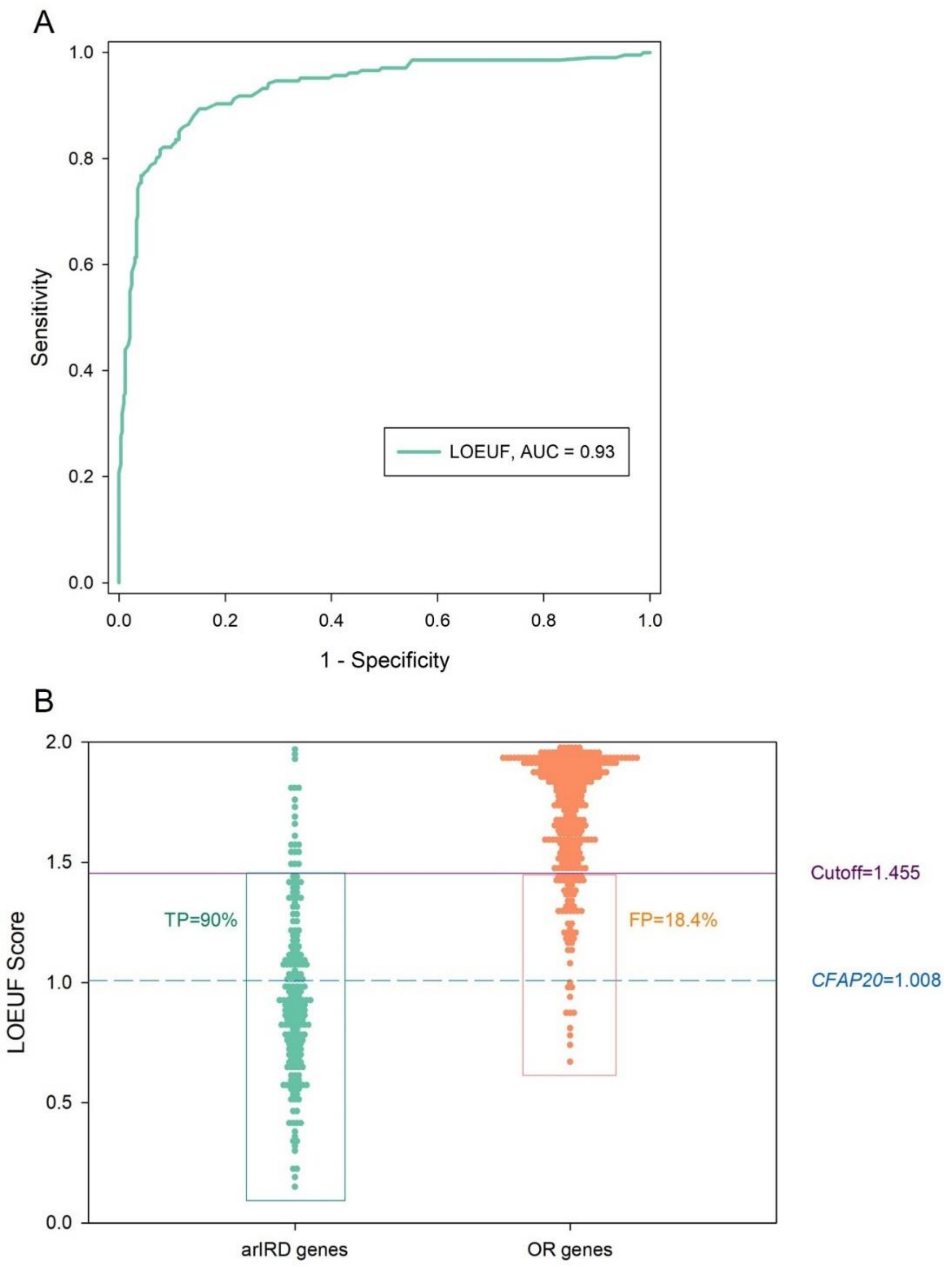

Supplementary Figure 3. Evaluation of the gnomad constraint metric LOEUF as an estimator of pathogenicity comparing autosomal recessive inherited retinal dystrophy (arIRD) genes vs. olfactory receptor (OR) genes.

A. ROC curve analysis showing the usefulness of the LOEUF score to discriminate arIRD genes (constrained group) from olfactory receptor genes (unconstrained group). B. Dot density plot showing the different distribution of the arIRD genes and the OR genes along the LOUEF spectrum that represents tolerance to inactivation. The cutoff for which sensitivity=90% is represented with a purple line (cutoff≤1.455). The *CFAP20* LOUEF value=1.008 is represented with a blue dashed line. Abbreviations: arIRD: autosomal recessive Inherited retinal dystrophy; FP: False positive rate; OR: olfactory receptor; TP: True positive rate.

Supplementary Table 1. List of variants comprising the training data classified as pathogenic or benign variants after an accurate curation.

Clasification	Genomic-level	Gene	Variant Type	cDNA	Protein
Pathogenic	Chr1:g.94467517A>C	*ABCA4*	missense	NM_000350.2:c.6179T>G	p.Leu2060Arg
Pathogenic	Chr1:g.94471056G>A	*ABCA4*	stop gain	NM_000350.2:c.6088C>T	p.Arg2030*
Pathogenic	Chr1:g.94473791C>T	*ABCA4*	exonic canonical splicing	NM_000350.2:c.5898G>A	r.spl?
Pathogenic	Chr1:g.94474323A>G	*ABCA4*	missense	NM_000350.2:c.5819T>C	p.Leu1940Pro
Pathogenic	Chr1:g.94476351C>T	*ABCA4*	canonical splicing	NM_000350.2:c.5714+5G>A	r.spl?
Pathogenic	Chr1:g.94485276_94485290del	*ABCA4*	in-frame	NM_000350.2:c.5044_5058del	p.Val1682_Val1686del
Pathogenic	Chr1:g.94486888G>C	*ABCA4*	missense	NM_000350.2:c.4926C>G	p.Ser1642Arg
Pathogenic	Chr1:g.94487503C>T	*ABCA4*	missense	NM_000350.2:c.4672G>A	p.Gly1558Arg
Pathogenic	Chr1:g.94495083G>A	*ABCA4*	missense	NM_000350.2:c.4457C>T	p.Pro1486Leu
Pathogenic	Chr1:g.94495167G>C	*ABCA4*	stop gain	NM_000350.2:c.4373C>G	p.Ser1458*
Pathogenic	Chr1:g.94496547C>T	*ABCA4*	canonical splicing	NM_000350.2:c.4253+5G>A	r.spl?
Pathogenic	Chr1:g.94506901C>A	*ABCA4*	missense	NM_000350.2:c.3386G>T	p.Arg1129Leu
Pathogenic	Chr1:g.94506923C>T	*ABCA4*	missense	NM_000350.2:c.3364G>A	p.Glu1122Lys
Pathogenic	Chr1:g.94509026G>A	*ABCA4*	missense	NM_000350.2:c.3056C>T	p.Thr1019Met
Pathogenic	Chr1:g.94522239A>T	*ABCA4*	missense	NM_000350.2:c.2300T>A	p.Val767Asp
Pathogenic	Chr1:g.94526212G>A	*ABCA4*	stop gain	NM_000350.2:c.2041C>T	p.Arg681*
Pathogenic	Chr1:g.94528132C>T	*ABCA4*	canonical splicing	NM_000350.2:c.1937+1G>A	r.spl
Pathogenic	Chr1:g.94528142A>C	*ABCA4*	missense	NM_000350.2:c.1928T>G	p.Val643Gly
Pathogenic	Chr1:g.94528251C>G	*ABCA4*	missense	NM_000350.2:c.1819G>C	p.Gly607Arg

Clasification	Genomic-level	Gene	Variant Type	cDNA	Protein
Pathogenic	Chr1:g.94528266G>A	*ABCA4*	missense	NM_000350.2:c.1804C>T	p.Arg602Trp
Pathogenic	Chr1:g.94528819G>A	*ABCA4*	missense	NM_000350.2:c.1609C>T	p.Arg537Cys
Pathogenic	Chr1:g.94544895G>A	*ABCA4*	stop gain	NM_000350.2:c.1222C>T	p.Arg408*
Pathogenic	Chr1:g.94564418G>A	*ABCA4*	stop gain	NM_000350.2:c.700C>T	p.Gln234*
Pathogenic	Chr1:g.94564447del	*ABCA4*	frameshift	NM_000350.2:c.671del	p.Thr224Argfs*17
Pathogenic	Chr1:g.94564484G>A	*ABCA4*	missense	NM_000350.2:c.634C>T	p.Arg212Cys
Pathogenic	Chr1:g.94568675T>C	*ABCA4*	missense	NM_000350.2:c.466A>G	p.Ile156Val
Pathogenic	Chr1:g.94574147G>A	*ABCA4*	missense	NM_000350.2:c.428C>T	p.Pro143Leu
Pathogenic	Chr1:g.94577073A>C	*ABCA4*	missense	NM_000350.2:c.223T>G	p.Cys75Gly
Pathogenic	Chr1:g.94586550G>A	*ABCA4*	missense	NM_000350.2:c.52C>T	p.Arg18Trp
Pathogenic	Chr1:g.94586570A>G	*ABCA4*	missense	NM_000350.2:c.32T>C	p.Leu11Pro
Pathogenic	Chr16:g.16282764A>G	*ABCC6*	missense	NM_001171.5:c.1703T>C	p.Phe568Ser
Pathogenic	Chr5:g.89939678del	*ADGRV1*	frameshift	NM_032119.3:c.2612del	p.Gly871Glufs*8
Pathogenic	Chr5:g.90078982_90078989del	*ADGRV1*	frameshift	NM_032119.3:c.13273_13280del	p.Leu4425Asnfs*8
Pathogenic	Chr17:g.6329086_6329091dup	*AIPL1*	in-frame	NM_014336.4:c.844_849dup	p.Glu282_Ala283dup
Pathogenic	Chr17:g.6329946C>G	*AIPL1*	missense	NM_014336.4:c.773G>C	p.Arg258Pro
Pathogenic	Chr2:g.73650091_73650092insA	*ALMS1*	frameshift	ENST00000264448.6:c.753_754insA	p.Ala252Serfs*8
Pathogenic	Chr11:g.66278554del	*BBS1*	frameshift	NM_024649.5:c.118del	p.Cys40Alafs*2
Pathogenic	Chr11:g.66293652T>G	*BBS1*	missense	NM_024649.5:c.1169T>G	p.Met390Arg
Pathogenic	Chr11:g.66298363A>G	*BBS1*	canonical splicing	NM_024649.5:c.1474-2A>G	r.spl
Pathogenic	Chr12:g.76740521del	*BBS10*	frameshift	NM_024685.4:c.1244del	p.His415Leufs*16
Pathogenic	Chr12:g.76741492G>C	*BBS10*	missense	NM_024685.4:c.273C>G	p.Cys91Trp
Pathogenic	Chr12:g.76741494dup	*BBS10*	frameshift	NM_024685.4:c.271dup	p.Cys91Leufs*5
Pathogenic	Chr16:g.56531672G>A	*BBS2*	stop gain	NM_031885.3:c.1780C>T	p.Arg594*

Clasification	Genomic-level	Gene	Variant Type	cDNA	Protein
Pathogenic	Chr16:g.56545070C>T	*BBS2*	canonical splicing	NM_031885.3:c.471+1G>A	r.spl
Pathogenic	Chr16:g.56545140del	*BBS2*	frameshift	NM_031885.3:c.402del	p.Ala136Argfs*65
Pathogenic	Chr16:g.56545141G>C	*BBS2*	missense	NM_031885.3:c.401C>G	p.Pro134Arg
Pathogenic	Chr11:g.61724355_61724356del	*BEST1*	frameshift	NM_001139443.1:c.341_342del	p.Leu114Glnfs*57
Pathogenic	Chr11:g.61724874C>T	*BEST1*	missense	NM_001139443.1:c.472C>T	p.Arg158Cys
Pathogenic	Chr11:g.61727516G>A	*BEST1*	canonical splicing	NM_001139443.1:c.920+1G>A	r.spl
Pathogenic	Chr11:g.61730047C>T	*BEST1*	missense	NM_001139443.1:c.1241C>T	p.Pro414Leu
Pathogenic	Chr4:g.15569358C>T	*CC2D2A*	missense	NM_001080522.2:c.3347C>T	p.Thr1116Met
Pathogenic	Chr10:g.73545491G>A	*CDH23*	missense	NM_022124.5:c.5816G>A	p.Arg1939Lys
Pathogenic	Chr10:g.73559034C>A	*CDH23*	stop gain	NM_022124.5:c.7221C>A	p.Tyr2407*
Pathogenic	Chr10:g.73567342G>A	*CDH23*	missense	NM_022124.5:c.8378G>A	p.Arg2793Gln
Pathogenic	Chr10:g.85970923T>C	*CDHR1*	canonical splicing	NM_033100.3:c.1485+2T>C	r.spl
Pathogenic	Chr20:g.34085578A>T	*CEP250*	stop gain	NM_007186.5:c.3337A>T	p.Lys1113*
Pathogenic	Chr12:g.88443069_88443073del	*CEP290*	frameshift	NM_025114.3:c.7328_7332del	p.Glu2443Glyfs*11
Pathogenic	Chr12:g.88453716del	*CEP290*	frameshift	NM_025114.3:c.6604del	p.Ile2202Leufs*24
Pathogenic	Chr12:g.88477732C>A	*CEP290*	canonical splicing	NM_025114.3:c.4705-1G>T	r.spl
Pathogenic	Chr12:g.88479860G>A	*CEP290*	stop gain	NM_025114.3:c.4393C>T	p.Arg1465*
Pathogenic	Chr12:g.88496677T>C	*CEP290*	missense	NM_025114.3:c.2929A>G	p.Arg977Gly
Pathogenic	Chr12:g.88524330T>A	*CEP290*	stop gain	NM_025114.3:c.508A>T	p.Lys170*
Pathogenic	Chr2:g.182423344G>A	*CERKL*	stop gain	NM_001030311.2:c.847C>T	p.Arg283*
Pathogenic	ChrX:g.85219054_85219057del	*CHM*	frameshift	NM_000390.3:c.315_318del	p.Ser105Argfs*20

Clasification	Genomic-level	Gene	Variant Type	cDNA	Protein
Pathogenic	ChrX:g.85219059T>C	*CHM*	canonical splicing	NM_000390.3:c.315-2A>G	r.spl
Pathogenic	ChrX:g.85282528G>C	*CHM*	stop gain	NM_000390.3:c.83C>G	p.Ser28*
Pathogenic	Chr3:g.150659368C>T	*CLRN1*	canonical splicing	NM_001195794.1:c.433+1G>A	r.spl
Pathogenic	Chr4:g.47954625G>A	*CNGA1*	stop gain	NM_001142564.1:c.301C>T	p.Arg101*
Pathogenic	Chr2:g.99012654T>C	*CNGA3*	missense	NM_001298.2:c.1021T>C	p.Ser341Pro
Pathogenic	Chr2:g.99013338C>T	*CNGA3*	missense	NM_001298.2:c.1705C>T	p.Arg569Cys
Pathogenic	Chr16:g.57938706_57938707del	*CNGB1*	stop gain	NM_001297.4:c.2565_2566del	p.Phe856*
Pathogenic	Chr16:g.57951243C>T	*CNGB1*	missense	NM_001297.4:c.2095G>A	p.Asp699Asn
Pathogenic	Chr8:g.87656009del	*CNGB3*	frameshift	NM_019098.4:c.1148del	p.Thr383Ilefs*13
Pathogenic	Chr1:g.197297979_197297987del	*CRB1*	in-frame	NM_201253.2:c.498_506del	p.Ile167_Gly169del
Pathogenic	Chr1:g.197298094_197298100del	*CRB1*	frameshift	NM_201253.2:c.613_619del	p.Ile205Aspfs*13
Pathogenic	Chr1:g.197313607G>A	*CRB1*	canonical splicing	NM_201253.2:c.848+1G>A	r.spl
Pathogenic	Chr1:g.197390660C>T	*CRB1*	missense	NM_201253.2:c.1702C>T	p.His568Tyr
Pathogenic	Chr1:g.197390718G>A	*CRB1*	missense	NM_201253.2:c.1760G>A	p.Cys587Tyr
Pathogenic	Chr1:g.197396682del	*CRB1*	frameshift	NM_201253.2:c.2227del	p.Val743Serfs*11
Pathogenic	Chr1:g.197396745C>T	*CRB1*	missense	NM_201253.2:c.2290C>T	p.Arg764Cys
Pathogenic	Chr1:g.197397037del	*CRB1*	frameshift	NM_201253.2:c.2582del	p.Asn861Ilefs*21
Pathogenic	Chr1:g.197398590T>A	*CRB1*	stop gain	NM_201253.2:c.2688T>A	p.Cys896*
Pathogenic	Chr1:g.197403836G>A	CRB1	exonic canonical splicing	NM_201253.2:c.2843G>A	r.spl?
Pathogenic	Chr1:g.197404292T>C	*CRB1*	missense	NM_201253.2:c.3299T>C	p.Ile1100Thr
Pathogenic	Chr1:g.197411405G>T	*CRB1*	stop gain	NM_201253.2:c.3988G>T	p.Glu1330*

Clasification	Genomic-level	Gene	Variant Type	cDNA	Protein
Pathogenic	Chr19:g.48342749A>G	*CRX*	missense	NM_000554.4:c.425A>G	p.Tyr142Cys
Pathogenic	Chr19:g.48343052del	*CRX*	frameshift	NM_000554.4:c.728del	p.Gly243Aspfs*128
Pathogenic	Chr6:g.65098735T>C	*EYS*	canonical splicing	NM_001292009.1:c.5928-2A>G	r.spl
Pathogenic	Chr6:g.65146137C>A	*EYS*	stop gain	NM_001292009.1:c.5857G>T	p.Glu1953*
Pathogenic	Chr6:g.66005808del	*EYS*	frameshift	NM_001292009.1:c.1971del	p.Ser658Valfs*4
Pathogenic	Chr2:g.62066830T>A	*FAM161A*	stop gain	NM_001201543.1:c.1309A>T	p.Arg437*
Pathogenic	Chr6:g.42141500C>T	*GUCA1A*	missense	NM_000409.4:c.149C>T	p.Pro50Leu
Pathogenic	Chr17:g.7906612C>T	*GUCY2D*	missense	NM_000180.3:c.247C>T	p.Arg83Cys
Pathogenic	Chr17:g.7915624C>A	*GUCY2D*	missense	NM_000180.3:c.1912C>A	p.Leu638Met
Pathogenic	Chr17:g.7917215C>T	*GUCY2D*	missense	NM_000180.3:c.2281C>T	p.Arg761Trp
Pathogenic	Chr17:g.7918019G>A	*GUCY2D*	missense	NM_000180.3:c.2513G>A	p.Arg838His
Pathogenic	Chr7:g.128038574T>C	*IMPDH1*	missense	NM_000883.3:c.968A>G	p.Lys323Arg
Pathogenic	Chr4:g.155665618G>A	*LRAT*	missense	NM_001301645.1:c.140G>A	p.Arg47Gln
Pathogenic	Chr4:g.155665641C>G	*LRAT*	missense	NM_001301645.1:c.163C>G	p.Arg55Gly
Pathogenic	Chr4:g.155665659T>A	*LRAT*	missense	NM_001301645.1:c.181T>A	p.Tyr61Asn
Pathogenic	Chr11:g.119216273del	*MFRP*	frameshift	NM_031433.3:c.498del	p.Asn167Thrfs*25
Pathogenic	Chr11:g.76867064C>T	*MYO7A*	missense	NM_000260.3:c.397C>T	p.His133Tyr
Pathogenic	Chr11:g.76867064dup	*MYO7A*	frameshift	NM_000260.3:c.397dup	p.His133Profs*7
Pathogenic	Chr11:g.76873969del	*MYO7A*	frameshift	NM_000260.3:c.1625del	p.Lys542Argfs*80
Pathogenic	Chr11:g.76885859A>G	*MYO7A*	missense	NM_000260.3:c.1993A>G	p.Ile665Val
Pathogenic	Chr11:g.76890874C>T	*MYO7A*	stop gain	NM_000260.3:c.2461C>T	p.Gln821*
Pathogenic	Chr11:g.76893006C>T	*MYO7A*	stop gain	NM_000260.3:c.2914C>T	p.Arg972*
Pathogenic	Chr11:g.76901162C>T	*MYO7A*	missense	NM_000260.3:c.3728C>T	p.Pro1243Leu
Pathogenic	Chr11:g.76903180del	*MYO7A*	frameshift	NM_000260.3:c.4009del	p.Glu1337Serfs*62
Pathogenic	Chr11:g.76919517C>T	*MYO7A*	stop gain	NM_000260.3:c.5899C>T	p.Arg1967*

Clasification	Genomic-level	Gene	Variant Type	cDNA	Protein
Pathogenic	Chr1:g.10035731G>A	*NMNAT1*	missense	NM_001297778.1:c.197G>A	p.Arg66Gln
Pathogenic	Chr1:g.10042688G>A	*NMNAT1*	missense	NM_001297778.1:c.769G>A	p.Glu257Lys
Pathogenic	NG_009113.1:g.5928A>C	*NR2E3*	canonical splicing	NM_014249.2:c.119-2A>C	r.spl
Pathogenic	NG_009113.1:g.6005_6013del	*NR2E3*	in-frame	NM_014249.2:c.194_202del	p.Asn65_Cys67del
Pathogenic	NG_009113.1:g.8055dup	*NR2E3*	frameshift	NM_014249.2:c.967dup	p.Met323Asnfs*18
Pathogenic	Chr14:g.24550505del	*NRL*	frameshift	NM_001354768.1:c.654del	p.Cys219Valfs*4
Pathogenic	Chr10:g.126086620G>T	*OAT*	missense	NM_000274.3:c.1211C>A	p.Ala404Asp
Pathogenic	Chr3:g.193355071G>A	*OPA1*	canonical splicing	NM_130837.2:c.1035+1G>A	r.spl
Pathogenic	Chr3:g.193355072T>C	*OPA1*	canonical splicing	NM_130837.2:c.1035+2T>C	r.spl
Pathogenic	Chr4:g.619800G>A	*PDE6B*	missense	NM_000283.3:c.385G>A	p.Glu129Lys
Pathogenic	Chr4:g.648670del	*PDE6B*	frameshift	NM_000283.3:c.985del	p.Val329Serfs*30
Pathogenic	Chr4:g.654360del	*PDE6B*	frameshift	NM_000283.3:c.1572del	p.Tyr525Thrfs*50
Pathogenic	Chr4:g.657565_657605del	*PDE6B*	frameshift	NM_000283.3:c.1927_1967del	p.Asn643Aspfs*29
Pathogenic	Chr4:g.658734G>A	*PDE6B*	canonical splicing	NM_000283.3:c.2193+1G>A	r.spl
Pathogenic	Chr10:g.102770539del	*PDZD7*	frameshift	NM_001195263.1:c.2107del	p.Ser703Valfs*20
Pathogenic	Chr1:g.46659965A>C	*POMGNT1*	missense	NM_001243766.1:c.860T>G	p.Ile287Ser
Pathogenic	Chr4:g.15991448C>A	*PROM1*	canonical splicing	NM_006017.2:c.1984-1G>T	r.spl
Pathogenic	Chr4:g.16035035C>T	*PROM1*	missense	NM_006017.2:c.401G>A	p.Arg134His
Pathogenic	Chr1:g.150316677C>A	*PRPF3*	missense	NM_004698.3:c.1466C>A	p.Ala489Asp
Pathogenic	Chr19:g.54622014G>A	*PRPF31*	canonical splicing	NM_015629.3:c.238+1G>A	r.spl

Clasification	Genomic-level	Gene	Variant Type	cDNA	Protein
Pathogenic	Chr19:g.54631485dup	*PRPF31*	frameshift	NM_015629.3:c.983dup	p.Asp329Argfs*146
Pathogenic	Chr17:g.1554110dup	*PRPF8*	frameshift	NM_006445.3:c.6994dup	p.Asp2332Glyfs*53
Pathogenic	Chr17:g.1554178T>C	*PRPF8*	missense	NM_006445.3:c.6926A>G	p.His2309Arg
Pathogenic	Chr6:g.42666160C>T	*PRPH2*	missense	NM_000322.4:c.914G>A	p.Gly305Asp
Pathogenic	Chr6:g.42672239G>C	*PRPH2*	stop gain	NM_000322.4:c.692C>G	p.Ser231*
Pathogenic	Chr6:g.42672350C>T	*PRPH2*	canonical splicing	NM_000322.4:c.582-1G>A	r.spl
Pathogenic	Chr6:g.42689579C>T	*PRPH2*	missense	NM_000322.4:c.494G>A	p.Cys165Tyr
Pathogenic	Chr6:g.42689652A>G	*PRPH2*	missense	NM_000322.4:c.421T>C	p.Tyr141His
Pathogenic	Chr6:g.42689937G>A	*PRPH2*	stop gain	NM_000322.4:c.136C>T	p.Arg46*
Pathogenic	Chr6:g.42689971_42689973del	PRPH2	in-frame	NM_000322.4:c.100_102del	p.Ile34del
Pathogenic	ChrX:g.106871904T>C	*PRPS1*	missense	NM_002764.3:c.46T>C	p.Ser16Pro
Pathogenic	Chr14:g.68191906T>C	*RDH12*	missense	NM_152443.2:c.278T>C	p.Leu93Pro
Pathogenic	Chr14:g.68191923C>A	*RDH12*	missense	NM_152443.2:c.295C>A	p.Leu99Ile
Pathogenic	Chr14:g.68193713C>T	*RDH12*	missense	NM_152443.2:c.464C>T	p.Thr155Ile
Pathogenic	Chr14:g.68193730C>T	*RDH12*	missense	NM_152443.2:c.481C>T	p.Arg161Trp
Pathogenic	Chr14:g.68193884G>C	*RDH12*	missense	NM_152443.2:c.635G>C	p.Arg212Pro
Pathogenic	Chr14:g.68200497C>T	*RDH12*	stop gain	NM_152443.2:c.883C>T	p.Arg295*
Pathogenic	Chr3:g.129247892G>A	*RHO*	missense	NM_000539.3:c.316G>A	p.Gly106Arg
Pathogenic	Chr3:g.129249760C>T	*RHO*	missense	NM_000539.3:c.403C>T	p.Arg135Trp
Pathogenic	Chr3:g.129251107G>A	*RHO*	missense	NM_000539.3:c.544G>A	p.Gly182Ser
Pathogenic	Chr3:g.129251615G>A	*RHO*	exonic canonical splicing	NM_000539.3:c.936G>A	r.spl?
Pathogenic	Chr3:g.129252449_129252458del	*RHO*	canonical splicing	NM_000539.3:c.937-2_944del	r.spl

Clasification	Genomic-level	Gene	Variant Type	cDNA	Protein
Pathogenic	Chr8:g.55533894_55533895dup	*RP1*	frameshift	NM_006269.1:c.368_369dup	p.Pro124Alafs*20
Pathogenic	Chr8:g.55537628C>T	*RP1*	stop gain	NM_006269.1:c.1186C>T	p.Arg396*
Pathogenic	Chr8:g.55538067C>G	*RP1*	stop gain	NM_006269.1:c.1625C>G	p.Ser542*
Pathogenic	Chr8:g.55538471C>T	*RP1*	stop gain	NM_006269.1:c.2029C>T	p.Arg677*
Pathogenic	Chr8:g.55542323C>T	*RP1*	stop gain	NM_006269.1:c.5881C>T	p.Gln1961*
Pathogenic	ChrX:g.46736996del	*RP2*	frameshift	NM_006915.2:c.940del	p.Ile314Leufs*2
Pathogenic	ChrX:g.46736996dup	*RP2*	frameshift	NM_006915.2:c.940dup	p.Ile314Asnfs*15
Pathogenic	Chr1:g.68903896A>G	*RPE65*	missense	NM_000329.2:c.1102T>C	p.Tyr368His
Pathogenic	Chr1:g.68904971T>G	*RPE65*	missense	NM_000329.2:c.761A>C	p.Glu254Ala
Pathogenic	Chr1:g.68910501_68910520del	*RPE65*	frameshift	NM_000329.2:c.292_311del	p.Ile98Hisfs*26
Pathogenic	Chr1:g.68914349T>C	*RPE65*	missense	NM_000329.2:c.52A>G	p.Thr18Ala
Pathogenic	ChrX:g.38145073_38145074del	*RPGR*	frameshift	NM_001034853.1:c.3178_3179del	p.Glu1060Argfs*18
Pathogenic	ChrX:g.38145596_38145597del	*RPGR*	frameshift	NM_001034853.1:c.2655_2656del	p.Glu886Glyfs*192
Pathogenic	ChrX:g.38145846_38145847del	*RPGR*	frameshift	NM_001034853.1:c.2405_2406del	p.Glu802Glyfs*32
Pathogenic	ChrX:g.38145992_38145995del	*RPGR*	frameshift	NM_001034853.1:c.2257_2260del	p.Gly753Lysfs*61
Pathogenic	ChrX:g.38146015_38146018del	*RPGR*	frameshift	NM_001034853.1:c.2234_2237del	p.Arg745Lysfs*69
Pathogenic	ChrX:g.38146058C>A	*RPGR*	stop gain	NM_001034853.1:c.2194G>T	p.Glu732*
Pathogenic	ChrX:g.38163933_38163934del	*RPGR*	frameshift	NM_001034853.1:c.888_889del	p.Ile297Lysfs*48
Pathogenic	ChrX:g.38163979_38163980del	*RPGR*	stop gain	NM_001034853.1:c.842_843del	p.Phe281*
Pathogenic	ChrX:g.38169907G>A	*RPGR*	stop gain	NM_001034853.1:c.739C>T	p.Gln247*
Pathogenic	ChrX:g.38170010_38170011del	*RPGR*	frameshift	NM_001034853.1:c.635_636del	p.Tyr212Cysfs*6
Pathogenic	ChrX:g.38176568C>A	*RPGR*	canonical splicing	NM_001034853.1:c.619+1G>T	r.spl
Pathogenic	ChrX:g.38176702del	*RPGR*	frameshift	NM_001034853.1:c.486del	p.Phe162Leufs*13
Pathogenic	ChrX:g.38180344T>A	*RPGR*	canonical splicing	NM_001034853.1:c.248-2A>T	r.spl

Clasification	Genomic-level	Gene	Variant Type	cDNA	Protein
Pathogenic	Chr14:g.21775922del	*RPGRIP1*	frameshift	NM_020366.3:c.833del	p.Arg278Hisfs*15
Pathogenic	Chr14:g.21785874del	*RPGRIP1*	frameshift	NM_020366.3:c.1171del	p.Ser391Alafs*12
Pathogenic	Chr14:g.21785923dup	RPGRIP1	frameshift	NM_020366.3:c.1220dup	p.Gln408Alafs*13
Pathogenic	Chr14:g.21793390G>A	*RPGRIP1*	canonical splicing	NM_020366.3:c.2216-1G>A	r.spl
Pathogenic	Chr14:g.21793477C>T	*RPGRIP1*	stop gain	NM_020366.3:c.2302C>T	p.Arg768*
Pathogenic	Chr14:g.21795967G>T	*RPGRIP1*	canonical splicing	NM_020366.3:c.2895+1G>T	r.spl
Pathogenic	Chr14:g.21796628C>T	*RPGRIP1*	stop gain	NM_020366.3:c.2941C>T	p.Arg981*
Pathogenic	Chr14:g.21819304_21819306del	*RPGRIP1*	in-frame	NM_020366.3:c.3790_3792del	p.Lys1264del
Pathogenic	ChrX:g.18660144A>G	*RS1*	missense	NM_000330.3:c.655T>C	p.Cys219Arg
Pathogenic	ChrX:g.18660161C>T	*RS1*	missense	NM_000330.3:c.638G>A	p.Arg213Gln
Pathogenic	ChrX:g.18662549C>G	*RS1*	canonical splicing	NM_000330.3:c.522+1G>C	r.spl
Pathogenic	ChrX:g.18662611T>C	*RS1*	missense	NM_000330.3:c.461A>G	p.Gln154Arg
Pathogenic	ChrX:g.18662650C>T	*RS1*	missense	NM_000330.3:c.422G>A	p.Arg141His
Pathogenic	ChrX:g.18674859C>T	*RS1*	stop gain	NM_000330.3:c.98G>A	p.Trp33*
Pathogenic	ChrX:g.18690135A>G	*RS1*	canonical splicing	NM_000330.3:c.52+2T>C	r.spl
Pathogenic	Chr2:g.234237188C>T	*SAG*	stop gain	NM_000541.4:c.577C>T	p.Arg193*
Pathogenic	Chr9:g.32541999dup	*TOPORS*	frameshift	NM_005802.4:c.2524dup	p.Thr842Asnfs*31
Pathogenic	Chr8:g.63973904del	*TTPA*	frameshift	NM_000370.3:c.744del	p.Glu249Asnfs*15
Pathogenic	Chr6:g.35471534C>A	*TULP1*	stop gain	NM_003322.5:c.1204G>T	p.Glu402*
Pathogenic	Chr6:g.35471589G>T	*TULP1*	missense	NM_003322.5:c.1149C>A	p.Asp383Glu
Pathogenic	Chr11:g.17552741del	*USH1C*	frameshift	NM_153676.3:c.347del	p.His116Profs*15
Pathogenic	Chr1:g.215808009G>T	*USH2A*	stop gain	NM_206933.2:c.15089C>A	p.Ser5030*

Clasification	Genomic-level	Gene	Variant Type	cDNA	Protein
Pathogenic	Chr1:g.215844436C>A	*USH2A*	stop gain	NM_206933.2:c.14011G>T	p.Glu4671*
Pathogenic	Chr1:g.215844625G>A	*USH2A*	stop gain	NM_206933.2:c.13822C>T	p.Arg4608*
Pathogenic	Chr1:g.215847445T>G	*USH2A*	missense	NM_206933.2:c.13808A>C	p.His4603Pro
Pathogenic	Chr1:g.215847677G>A	*USH2A*	stop gain	NM_206933.2:c.13576C>T	p.Arg4526*
Pathogenic	Chr1:g.215847722C>T	*USH2A*	missense	NM_206933.2:c.13531G>A	p.Ala4511Thr
Pathogenic	Chr1:g.215847879del	*USH2A*	frameshift	NM_206933.2:c.13374del	p.Glu4458Aspfs*3
Pathogenic	Chr1:g.215848064C>A	*USH2A*	stop gain	NM_206933.2:c.13189G>T	p.Glu4397*
Pathogenic	Chr1:g.215848679G>A	*USH2A*	missense	NM_206933.2:c.12574C>T	p.Arg4192Cys
Pathogenic	Chr1:g.215848684A>G	*USH2A*	missense	NM_206933.2:c.12569T>C	p.Val4190Ala
Pathogenic	Chr1:g.215848796del	*USH2A*	frameshift	NM_206933.2:c.12457del	p.Ala4153Profs*14
Pathogenic	Chr1:g.215848882G>C	*USH2A*	missense	NM_206933.2:c.12371C>G	p.Pro4124Arg
Pathogenic	Chr1:g.215853490C>T	*USH2A*	canonical splicing	NM_206933.2:c.12294+1G>A	r.spl
Pathogenic	Chr1:g.215853720T>C	*USH2A*	canonical splicing	NM_206933.2:c.12067-2A>G	r.spl
Pathogenic	Chr1:g.215914717C>T	USH2A	exonic canonical splicing	NM_206933.2:c.11711G>A	r.spl?
Pathogenic	Chr1:g.215933077C>T	*USH2A*	missense	NM_206933.2:c.11156G>A	p.Arg3719His
Pathogenic	Chr1:g.215955412G>A	*USH2A*	missense	NM_206933.2:c.10712C>T	p.Thr3571Met
Pathogenic	Chr1:g.215956262G>A	*USH2A*	missense	NM_206933.2:c.10403C>T	p.Pro3468Leu
Pathogenic	Chr1:g.215960065G>T	*USH2A*	stop gain	NM_206933.2:c.10334C>A	p.Ser3445*
Pathogenic	Chr1:g.215960126_215960127dup	*USH2A*	frameshift	NM_206933.2:c.10272_10273dup	p.Cys3425Phefs*4
Pathogenic	Chr1:g.215963510C>T	*USH2A*	missense	NM_206933.2:c.10073G>A	p.Cys3358Tyr
Pathogenic	Chr1:g.215972408A>G	*USH2A*	missense	NM_206933.2:c.9799T>C	p.Cys3267Arg

Clasification	Genomic-level	Gene	Variant Type	cDNA	Protein
Pathogenic	Chr1:g.215990476G>A	*USH2A*	missense	NM_206933.2:c.9433C>T	p.Leu3145Phe
Pathogenic	Chr1:g.216062182G>T	*USH2A*	stop gain	NM_206933.2:c.7809C>A	p.Cys2603*
Pathogenic	Chr1:g.216073486G>A	*USH2A*	missense	NM_206933.2:c.7525C>T	p.Arg2509Trp
Pathogenic	Chr1:g.216138812G>A	*USH2A*	stop gain	NM_206933.2:c.6967C>T	p.Arg2323*
Pathogenic	Chr1:g.216270538G>A	*USH2A*	stop gain	NM_206933.2:c.4645C>T	p.Arg1549*
Pathogenic	Chr1:g.216348747C>A	*USH2A*	stop gain	NM_206933.2:c.4474G>T	p.Glu1492*
Pathogenic	Chr1:g.216363636A>G	*USH2A*	missense	NM_206933.2:c.4325T>C	p.Phe1442Ser
Pathogenic	Chr1:g.216371824G>A	*USH2A*	missense	NM_206933.2:c.3914C>T	p.Pro1305Leu
Pathogenic	Chr1:g.216380719T>C	*USH2A*	missense	NM_206933.2:c.3212A>G	p.Asn1071Ser
Pathogenic	Chr1:g.216405371del	*USH2A*	frameshift	NM_206933.2:c.2917del	p.Gln973Lysfs*38
Pathogenic	Chr1:g.216420437del	*USH2A*	frameshift	NM_206933.2:c.2299del	p.Glu767Serfs*21
Pathogenic	Chr1:g.216420460C>A	*USH2A*	missense	NM_206933.2:c.2276G>T	p.Cys759Phe
Pathogenic	Chr1:g.216424240C>T	*USH2A*	canonical splicing	NM_206933.2:c.2167+5G>A	r.spl?
Pathogenic	Chr1:g.216424377C>A	*USH2A*	stop gain	NM_206933.2:c.2035G>T	p.Gly679*
Pathogenic	Chr1:g.216462754T>C	*USH2A*	canonical splicing	NM_206933.2:c.1841-2A>G	r.spl
Pathogenic	Chr1:g.216495263A>G	*USH2A*	missense	NM_206933.2:c.1606T>C	p.Cys536Arg
Pathogenic	Chr1:g.216495309G>T	*USH2A*	stop gain	NM_206933.2:c.1560C>A	p.Cys520*
Pathogenic	Chr1:g.216496975C>T	*USH2A*	missense	NM_206933.2:c.1391G>A	p.Arg464His
Pathogenic	Chr1:g.216497624del	*USH2A*	frameshift	NM_206933.2:c.1214del	p.Asn405Ilefs*3
Pathogenic	Chr1:g.216498790G>A	*USH2A*	missense	NM_206933.2:c.1000C>T	p.Arg334Trp
Pathogenic	Chr1:g.216498867_216498870dup	*USH2A*	frameshift	NM_206933.2:c.920_923dup	p.His308Glnfs*16
Pathogenic	Chr1:g.216498882C>T	*USH2A*	missense	NM_206933.2:c.908G>A	p.Arg303His
Pathogenic	Chr1:g.216498883G>T	*USH2A*	missense	NM_206933.2:c.907C>A	p.Arg303Ser

Clasification	Genomic-level	Gene	Variant Type	cDNA	Protein
Pathogenic	Chr1:g.216500961G>A	*USH2A*	stop gain	NM_206933.2:c.820C>T	p.Arg274*
Pathogenic	Chr1:g.216538325C>A	*USH2A*	missense	NM_206933.2:c.754G>T	p.Gly252Cys
Pathogenic	Chr1:g.216595504C>T	*USH2A*	missense	NM_206933.2:c.175G>A	p.Gly59Arg
Pathogenic	Chr4:g.6303282G>A	*WFS1*	missense	NM_001145853.1:c.1760G>A	p.Arg587Gln
Pathogenic	Chr4:g.6303573C>T	*WFS1*	missense	NM_001145853.1:c.2051C>T	p.Ala684Val
Benign	Chr1:g.94463648G>A	*ABCA4*	synonymous	NM_000350.2:c.6498C>T	p.Ile2166=
Benign	Chr1:g.94467426C>T	*ABCA4*	synonymous	NM_000350.2:c.6270G>A	p.Pro2090=
Benign	Chr1:g.94474351C>T	*ABCA4*	missense	NM_000350.2:c.5791G>A	p.Gly1931Ser
Benign	Chr1:g.94490566C>T	*ABCA4*	synonymous	NM_000350.2:c.4578G>A	p.Thr1526=
Benign	Chr1:g.94495191A>G	*ABCA4*	non-canonical splicing	NM_000350.2:c.4353-4T>C	p.?
Benign	Chr1:g.94497337C>T	*ABCA4*	synonymous	NM_000350.2:c.4125G>A	p.Ala1375=
Benign	Chr1:g.94497490C>T	*ABCA4*	synonymous	NM_000350.2:c.3972G>A	p.Ala1324=
Benign	Chr1:g.94502328G>A	*ABCA4*	missense	NM_000350.2:c.3830C>T	p.Thr1277Met
Benign	Chr1:g.94512603G>A	*ABCA4*	synonymous	NM_000350.2:c.2790C>T	p.Cys930=
Benign	Chr1:g.94514466T>C	*ABCA4*	missense	NM_000350.2:c.2701A>G	p.Thr901Ala
Benign	Chr1:g.94520768G>A	*ABCA4*	missense	NM_000350.2:c.2486C>T	p.Thr829Met
Benign	Chr1:g.94543278G>A	*ABCA4*	missense	NM_000350.2:c.1522C>T	p.Arg508Cys
Benign	Chr1:g.94543300C>T	*ABCA4*	synonymous	NM_000350.2:c.1500G>A	p.Arg500=
Benign	Chr1:g.94544235G>A	*ABCA4*	missense	NM_000350.2:c.1267C>T	p.His423Tyr
Benign	Chr1:g.94546104A>G	*ABCA4*	synonymous	NM_000350.2:c.1029T>C	p.Asn343=
Benign	Chr1:g.94564392G>A	*ABCA4*	synonymous	NM_000350.2:c.726C>T	p.Asp242=
Benign	Chr16:g.16248775G>A	*ABCC6*	synonymous	NM_001171.5:c.3996C>T	p.His1332=
Benign	Chr16:g.16248816C>A	*ABCC6*	missense	NM_001171.5:c.3955G>T	p.Ala1319Ser
Benign	Chr16:g.16259618G>A	*ABCC6*	synonymous	NM_001171.5:c.3168C>T	p.Asp1056=
Benign	Chr20:g.25282971G>A	*ABHD12*	synonymous	NM_015600.4:c.1041C>T	p.Ile347=

Clasification	Genomic-level	Gene	Variant Type	cDNA	Protein
Benign	Chr20:g.25284255G>A	*ABHD12*	synonymous	NM_015600.4:c.960C>T	p.His320=
Benign	Chr20:g.25290152C>T	*ABHD12*	missense	NM_015600.4:c.679G>A	p.Val227Ile
Benign	Chr10:g.27512126T>C	*ACBD5*	non-canonical splicing	NM_001352568.1:c.544+10A>G	p.?
Benign	Chr8:g.38874784C>A	*ADAM9*	missense	NM_003816.2:c.457C>A	p.Gln153Lys
Benign	Chr5:g.89854743T>C	*ADGRV1*	non-canonical splicing	NM_032119.3:c.22+9T>C	p.?
Benign	Chr5:g.89910839A>G	*ADGRV1*	non-canonical splicing	NM_032119.3:c.207+3A>G	r.spl?
Benign	Chr5:g.89925235G>T	*ADGRV1*	missense	NM_032119.3:c.1718G>T	p.Gly573Val
Benign	Chr5:g.89930940G>A	*ADGRV1*	missense	NM_032119.3:c.1849G>A	p.Val617Met
Benign	Chr5:g.89933637G>A	*ADGRV1*	synonymous	NM_032119.3:c.2112G>A	p.Pro704=
Benign	Chr5:g.89943443G>T	*ADGRV1*	missense	NM_032119.3:c.3151G>T	p.Asp1051Tyr
Benign	Chr5:g.89949740A>G	*ADGRV1*	missense	NM_032119.3:c.4349A>G	p.Lys1450Arg
Benign	Chr5:g.89954009G>A	*ADGRV1*	missense	NM_032119.3:c.4666G>A	p.Glu1556Lys
Benign	Chr5:g.89971951A>G	*ADGRV1*	missense	NM_032119.3:c.5368A>G	p.Ile1790Val
Benign	Chr5:g.89988445T>C	*ADGRV1*	synonymous	NM_032119.3:c.6975T>C	p.Asp2325=
Benign	Chr5:g.89988464A>T	*ADGRV1*	missense	NM_032119.3:c.6994A>T	p.Ile2332Phe
Benign	Chr5:g.89990343C>T	*ADGRV1*	synonymous	NM_032119.3:c.7770C>T	p.Ser2590=
Benign	Chr5:g.89999487A>G	*ADGRV1*	missense	NM_032119.3:c.8161A>G	p.Ile2721Val
Benign	Chr5:g.90007092C>G	*ADGRV1*	missense	NM_032119.3:c.8995C>G	p.Gln2999Glu
Benign	Chr5:g.90012539G>A	*ADGRV1*	missense	NM_032119.3:c.9440G>A	p.Arg3147Gln
Benign	Chr5:g.90012552G>A	*ADGRV1*	non-canonical splicing	NM_032119.3:c.9447+6G>A	r.spl?
Benign	Chr5:g.90020788G>A	*ADGRV1*	synonymous	NM_032119.3:c.9888G>A	p.Gly3296=
Benign	Chr5:g.90024584C>T	*ADGRV1*	synonymous	NM_032119.3:c.10260C>T	p.Phe3420=

Clasification	Genomic-level	Gene	Variant Type	cDNA	Protein
Benign	Chr5:g.90041524A>G	*ADGRV1*	missense	NM_032119.3:c.10886A>G	p.Lys3629Arg
Benign	Chr5:g.90050795C>A	*ADGRV1*	canonical splicing	NM_032119.3:c.11378-5C>A	p.?
Benign	Chr5:g.90051001C>T	*ADGRV1*	missense	NM_032119.3:c.11579C>T	p.Pro3860Leu
Benign	Chr5:g.90052843C>T	*ADGRV1*	synonymous	NM_032119.3:c.11805C>T	p.Asn3935=
Benign	Chr5:g.90055259G>A	*ADGRV1*	missense	NM_032119.3:c.11974G>A	p.Asp3992Asn
Benign	Chr5:g.90070067G>A	*ADGRV1*	missense	NM_032119.3:c.12350G>A	p.Arg4117His
Benign	Chr5:g.90079151A>G	*ADGRV1*	non-canonical splicing	NM_032119.3:c.13433+9A>G	p.?
Benign	Chr5:g.90083991A>T	*ADGRV1*	missense	NM_032119.3:c.13757A>T	p.Glu4586Val
Benign	Chr5:g.90103532G>T	*ADGRV1*	missense	NM_032119.3:c.14950G>T	p.Ala4984Ser
Benign	Chr5:g.90106961A>T	*ADGRV1*	missense	NM_032119.3:c.15884A>T	p.Glu5295Val
Benign	Chr5:g.90149238A>G	*ADGRV1*	missense	NM_032119.3:c.17342A>G	p.Gln5781Arg
Benign	Chr5:g.90149281C>T	*ADGRV1*	synonymous	NM_032119.3:c.17385C>T	p.Val5795=
Benign	Chr5:g.90151576G>A	*ADGRV1*	synonymous	NM_032119.3:c.17613G>A	p.Gln5871=
Benign	Chr5:g.90398027dup	*ADGRV1*	non-canonical splicing	NM_032119.3:c.18311-9dup	p.?
Benign	Chr5:g.90445884A>G	*ADGRV1*	missense	NM_032119.3:c.18470A>G	p.Asn6157Ser
Benign	Chr6:g.135749885C>T	*AHI1*	synonymous	NM_001134830.1:c.2505G>A	p.Arg835=
Benign	Chr6:g.135763812G>T	*AHI1*	missense	NM_001134830.1:c.1820C>A	p.Ala607Glu
Benign	Chr17:g.6328871_6328882del	*AIPL1*	in-frame	NM_014336.4:c.1053_1064del	p.Ala352_Pro355del
Benign	Chr17:g.6328925T>G	*AIPL1*	missense	NM_014336.4:c.1010A>C	p.Glu337Ala
Benign	Chr2:g.73613059G>A	*ALMS1*	synonymous	ENST00000264448.6:c.63G>A	p.Glu21=
Benign	Chr2:g.73646454C>G	*ALMS1*	non-canonical splicing	ENST00000264448.6:c.646+8C>G	p.?
Benign	Chr2:g.73651975T>C	*ALMS1*	synonymous	ENST00000264448.6:c.1182T>C	p.Tyr394=

Clasification	Genomic-level	Gene	Variant Type	cDNA	Protein
Benign	Chr2:g.73676823A>G	*ALMS1*	missense	ENST00000264448.6:c.3166A>G	p.Ser1056Gly
Benign	Chr2:g.73677535G>C	*ALMS1*	missense	ENST00000264448.6:c.3878G>C	p.Ser1293Thr
Benign	Chr2:g.73677554G>A	*ALMS1*	synonymous	ENST00000264448.6:c.3897G>A	p.Ser1299=
Benign	Chr2:g.73677663A>G	*ALMS1*	missense	ENST00000264448.6:c.4006A>G	p.Thr1336Ala
Benign	Chr2:g.73677803A>G	*ALMS1*	synonymous	ENST00000264448.6:c.4146A>G	p.Gln1382=
Benign	Chr2:g.73679521C>A	*ALMS1*	missense	ENST00000264448.6:c.5864C>A	p.Pro1955Gln
Benign	Chr2:g.73679661A>G	*ALMS1*	missense	ENST00000264448.6:c.6004A>G	p.Ile2002Val
Benign	Chr2:g.73681101G>A	*ALMS1*	missense	ENST00000264448.6:c.7444G>A	p.Val2482Met
Benign	Chr2:g.73717531A>G	*ALMS1*	synonymous	ENST00000264448.6:c.8442A>G	p.Ser2814=
Benign	Chr2:g.73718104C>T	*ALMS1*	synonymous	ENST00000264448.6:c.9015C>T	p.His3005=
Benign	Chr2:g.73746995G>A	*ALMS1*	synonymous	ENST00000264448.6:c.9630G>A	p.Lys3210=
Benign	Chr2:g.73799792G>C	*ALMS1*	synonymous	ENST00000264448.6:c.10785G>C	p.Val3595=
Benign	Chr2:g.73800108C>A	*ALMS1*	synonymous	ENST00000264448.6:c.11101C>A	p.Arg3701=
Benign	Chr2:g.73826625T>C	*ALMS1*	missense	ENST00000264448.6:c.11642T>C	p.Met3881Thr
Benign	Chr2:g.73828417C>G	*ALMS1*	missense	ENST00000264448.6:c.11965C>G	p.Pro3989Ala
Benign	Chr11:g.66278141C>T	*BBS1*	missense	NM_024649.5:c.11C>T	p.Ala4Val
Benign	Chr11:g.66278186A>G	*BBS1*	non-canonical splicing	NM_024649.5:c.47+9A>G	p.?
Benign	Chr11:g.66291054C>A	*BBS1*	non-canonical splicing	NM_024649.5:c.951+7C>A	p.?
Benign	Chr11:g.66291280T>G	*BBS1*	missense	NM_024649.5:c.1037T>G	p.Val346Gly
Benign	Chr11:g.66293661G>A	*BBS1*	missense	NM_024649.5:c.1178G>A	p.Arg393Gln
Benign	Chr12:g.76740554T>C	*BBS10*	missense	NM_024685.4:c.1211A>G	p.His404Arg
Benign	Chr12:g.76740654A>G	*BBS10*	missense	NM_024685.4:c.1111T>C	p.Cys371Arg
Benign	Chr12:g.76740879C>T	*BBS10*	missense	NM_024685.4:c.886G>A	p.Ala296Thr
Benign	Chr16:g.56533778C>T	*BBS2*	missense	NM_031885.3:c.1439G>A	p.Arg480Gln

Clasification	Genomic-level	Gene	Variant Type	cDNA	Protein
Benign	Chr16:g.56540114A>G	*BBS2*	missense	NM_031885.3:c.635T>C	p.Met212Thr
Benign	Chr16:g.56544843A>G	*BBS2*	non-canonical splicing	NM_031885.3:c.472-10T>C	p.?
Benign	Chr4:g.122749355C>G	*BBS7*	missense	NM_176824.2:c.1960G>C	p.Asp654His
Benign	Chr11:g.61724853C>T	*BEST1*	non-canonical splicing	NM_001139443.1:c.457-6C>T	p.?
Benign	Chr11:g.61725609_61725614dup	*BEST1*	non-canonical splicing	NM_001139443.1:c.535-9_535-4dup	p.?
Benign	Chr11:g.61730145T>C	*BEST1*	missense	NM_001139443.1:c.1339T>C	p.Ser447Pro
Benign	Chr11:g.119210464T>G	*C1QTNF5*	synonymous	NM_001278431.1:c.309A>C	p.Arg103=
Benign	Chr11:g.119210565G>C	*C1QTNF5*	non-canonical splicing	NM_001278431.1:c.215-7C>G	p.?
Benign	Chr19:g.6678189G>A	*C3*	synonymous	NM_000064.3:c.4824C>T	p.Ser1608=
Benign	Chr19:g.6686754C>T	*C3*	non-canonical splicing	NM_000064.3:c.3646+3G>A	r.spl?
Benign	Chr19:g.6693437C>A	*C3*	synonymous	NM_000064.3:c.3216G>T	p.Arg1072=
Benign	Chr19:g.6694472G>A	*C3*	missense	NM_000064.3:c.3124C>T	p.Arg1042Trp
Benign	Chr19:g.6707142G>A	*C3*	synonymous	NM_000064.3:c.2190C>T	p.Tyr730=
Benign	Chr19:g.6707877C>G	*C3*	missense	NM_000064.3:c.1909G>C	p.Gly637Arg
Benign	Chr19:g.6712591C>G	*C3*	synonymous	NM_000064.3:c.1047G>C	p.Val349=
Benign	Chr17:g.58234828A>G	*CA4*	synonymous	NM_000717.4:c.309A>G	p.Gly103=
Benign	Chr17:g.58234928A>C	*CA4*	missense	NM_000717.4:c.409A>C	p.Met137Leu
Benign	ChrX:g.49061669G>A	*CACNA1F*	synonymous	NM_005183.3:c.5862C>T	p.Asp1954=
Benign	ChrX:g.49062205G>A	*CACNA1F*	synonymous	NM_005183.3:c.5574C>T	p.Gly1858=
Benign	ChrX:g.49065081C>G	*CACNA1F*	missense	NM_005183.3:c.5050G>C	p.Gly1684Arg
Benign	ChrX:g.49076230C>T	*CACNA1F*	synonymous	NM_005183.3:c.2439G>A	p.Glu813=

Clasification	Genomic-level	Gene	Variant Type	cDNA	Protein
Benign	ChrX:g.49076241C>T	*CACNA1F*	missense	NM_005183.3:c.2428G>A	p.Glu810Lys
Benign	ChrX:g.49079179T>G	*CACNA1F*	missense	NM_005183.3:c.2237A>C	p.Asn746Thr
Benign	ChrX:g.49081369A>G	*CACNA1F*	synonymous	NM_005183.3:c.1764T>C	p.Tyr588=
Benign	Chr11:g.76804776G>A	*CAPN5*	missense	NM_004055.4:c.214G>A	p.Asp72Asn
Benign	Chr4:g.15539578T>C	*CC2D2A*	synonymous	NM_001080522.2:c.1821T>C	p.Ile607=
Benign	Chr4:g.15539703C>T	*CC2D2A*	missense	NM_001080522.2:c.1946C>T	p.Thr649Met
Benign	Chr4:g.15572054C>T	*CC2D2A*	missense	NM_001080522.2:c.3529C>T	p.Arg1177Cys
Benign	Chr10:g.73269961C>T	*CDH23*	missense	NM_022124.5:c.268C>T	p.Arg90Trp
Benign	Chr10:g.73270925G>A	*CDH23*	missense	NM_022124.5:c.385G>A	p.Ala129Thr
Benign	Chr10:g.73326687C>T	*CDH23*	synonymous	NM_022124.5:c.618C>T	p.Asn206=
Benign	Chr10:g.73330636C>T	*CDH23*	synonymous	NM_022124.5:c.714C>T	p.Asn238=
Benign	Chr10:g.73375284C>T	*CDH23*	synonymous	NM_022124.5:c.856C>T	p.Leu286=
Benign	Chr10:g.73377062A>G	*CDH23*	missense	NM_022124.5:c.1046A>G	p.Asn349Ser
Benign	Chr10:g.73447449G>A	*CDH23*	missense	NM_022124.5:c.2032G>A	p.Val678Ile
Benign	Chr10:g.73453966C>T	*CDH23*	missense	NM_022124.5:c.2239C>T	p.Arg747Cys
Benign	Chr10:g.73461773G>A	*CDH23*	non-canonical splicing	NM_022124.5:c.2398-6G>A	p.?
Benign	Chr10:g.73464685C>T	*CDH23*	synonymous	NM_022124.5:c.2751C>T	p.Leu917=
Benign	Chr10:g.73466722G>A	*CDH23*	missense	NM_022124.5:c.3022G>A	p.Val1008Met
Benign	Chr10:g.73472463G>A	*CDH23*	missense	NM_022124.5:c.3262G>A	p.Val1088Met
Benign	Chr10:g.73472486G>C	*CDH23*	synonymous	NM_022124.5:c.3285G>C	p.Val1095=
Benign	Chr10:g.73483817G>A	*CDH23*	missense	NM_022124.5:c.3385G>A	p.Ala1129Thr
Benign	Chr10:g.73491829C>T	*CDH23*	synonymous	NM_022124.5:c.3801C>T	p.Thr1267=
Benign	Chr10:g.73491858C>T	*CDH23*	missense	NM_022124.5:c.3830C>T	p.Thr1277Ile
Benign	Chr10:g.73491873A>G	*CDH23*	missense	NM_022124.5:c.3845A>G	p.Asn1282Ser
Benign	Chr10:g.73492073C>T	*CDH23*	missense	NM_022124.5:c.4045C>T	p.Arg1349Cys

Clasification	Genomic-level	Gene	Variant Type	cDNA	Protein
Benign	Chr10:g.73500672G>A	*CDH23*	missense	NM_022124.5:c.4582G>A	p.Glu1528Lys
Benign	Chr10:g.73545471G>A	*CDH23*	synonymous	NM_022124.5:c.5796G>A	p.Pro1932=
Benign	Chr10:g.73550106C>T	*CDH23*	synonymous	NM_022124.5:c.5985C>T	p.Tyr1995=
Benign	Chr10:g.73550937C>T	*CDH23*	missense	NM_022124.5:c.6098C>T	p.Ser2033Leu
Benign	Chr10:g.73553333C>T	*CDH23*	synonymous	NM_022124.5:c.6648C>T	p.Ala2216=
Benign	Chr10:g.73556957G>A	*CDH23*	missense	NM_022124.5:c.6809G>A	p.Arg2270His
Benign	Chr10:g.73558877T>C	*CDH23*	missense	NM_022124.5:c.7064T>C	p.Ile2355Thr
Benign	Chr10:g.73560490A>C	*CDH23*	missense	NM_022124.5:c.7460A>C	p.Asn2487Thr
Benign	Chr10:g.73563105G>A	*CDH23*	synonymous	NM_022124.5:c.7800G>A	p.Glu2600=
Benign	Chr10:g.73567460G>A	*CDH23*	synonymous	NM_022124.5:c.8496G>A	p.Val2832=
Benign	Chr10:g.73569583T>C	*CDH23*	missense	NM_022124.5:c.8729T>C	p.Met2910Thr
Benign	Chr10:g.73569597C>T	*CDH23*	missense	NM_022124.5:c.8743C>T	p.Arg2915Cys
Benign	Chr10:g.73569757T>C	*CDH23*	missense	NM_022124.5:c.8903T>C	p.Val2968Ala
Benign	Chr10:g.73569767C>T	*CDH23*	synonymous	NM_022124.5:c.8913C>T	p.Phe2971=
Benign	Chr10:g.73569837C>T	*CDH23*	non-canonical splicing	NM_022124.5:c.8979+4C>T	r.spl?
Benign	Chr10:g.73571273G>A	*CDH23*	synonymous	NM_022124.5:c.9204G>A	p.Ala3068=
Benign	Chr10:g.73574953G>A	*CDH23*	missense	NM_022124.5:c.9983G>A	p.Arg3328His
Benign	Chr10:g.73575014C>G	*CDH23*	synonymous	NM_022124.5:c.10044C>G	p.Pro3348=
Benign	Chr10:g.85958847C>T	*CDHR1*	synonymous	NM_033100.3:c.408C>T	p.Ile136=
Benign	Chr10:g.85961583C>T	*CDHR1*	synonymous	NM_033100.3:c.546C>T	p.Ala182=
Benign	Chr10:g.85961584G>A	*CDHR1*	missense	NM_033100.3:c.547G>A	p.Val183Met
Benign	Chr10:g.85967950G>A	*CDHR1*	synonymous	NM_033100.3:c.984G>A	p.Ala328=
Benign	Chr10:g.85968099G>A	*CDHR1*	missense	NM_033100.3:c.1133G>A	p.Arg378Gln
Benign	Chr10:g.85968557C>T	*CDHR1*	synonymous	NM_033100.3:c.1240C>T	p.Leu414=
Benign	Chr10:g.85970887A>C	*CDHR1*	missense	NM_033100.3:c.1451A>C	p.Asn484Thr

Clasification	Genomic-level	Gene	Variant Type	cDNA	Protein
Benign	Chr10:g.85970897G>A	*CDHR1*	synonymous	NM_033100.3:c.1461G>A	p.Gly487=
Benign	Chr10:g.85970906C>T	*CDHR1*	synonymous	NM_033100.3:c.1470C>T	p.Ser490=
Benign	Chr10:g.85970911T>G	*CDHR1*	missense	NM_033100.3:c.1475T>G	p.Val492Gly
Benign	Chr10:g.85971970C>G	*CDHR1*	missense	NM_033100.3:c.1589C>G	p.Thr530Ser
Benign	Chr10:g.85971975C>G	*CDHR1*	missense	NM_033100.3:c.1594C>G	p.Pro532Ala
Benign	Chr10:g.85972913G>A	*CDHR1*	missense	NM_033100.3:c.1849G>A	p.Ala617Thr
Benign	Chr10:g.85974019G>A	*CDHR1*	missense	NM_033100.3:c.2222G>A	p.Arg741His
Benign	Chr10:g.85974188A>C	*CDHR1*	synonymous	NM_033100.3:c.2391A>C	p.Pro797=
Benign	Chr10:g.85974277C>G	*CDHR1*	missense	NM_033100.3:c.2480C>G	p.Pro827Arg
Benign	Chr20:g.34060636C>T	*CEP250*	missense	NM_007186.5:c.1189C>T	p.Arg397Trp
Benign	Chr20:g.34067081C>T	*CEP250*	missense	NM_007186.5:c.2120C>T	p.Thr707Met
Benign	Chr20:g.34079058G>T	*CEP250*	missense	NM_007186.5:c.2775G>T	p.Lys925Asn
Benign	Chr20:g.34090817C>T	*CEP250*	synonymous	NM_007186.5:c.4620C>T	p.Ser1540=
Benign	Chr12:g.88449354T>A	*CEP290*	missense	NM_025114.3:c.6959A>T	p.Gln2320Leu
Benign	Chr12:g.88453691C>T	*CEP290*	missense	NM_025114.3:c.6629G>A	p.Arg2210His
Benign	Chr12:g.88462318T>C	*CEP290*	missense	NM_025114.3:c.6116A>G	p.Asp2039Gly
Benign	Chr12:g.88477679T>C	*CEP290*	missense	NM_025114.3:c.4757A>G	p.His1586Arg
Benign	Chr12:g.88480212T>C	*CEP290*	missense	NM_025114.3:c.4258A>G	p.Ile1420Val
Benign	Chr12:g.88481597G>A	*CEP290*	missense	NM_025114.3:c.4154C>T	p.Thr1385Ile
Benign	Chr12:g.88483091G>A	*CEP290*	synonymous	NM_025114.3:c.3747C>T	p.Leu1249=
Benign	Chr12:g.88500653C>T	*CEP290*	synonymous	NM_025114.3:c.2616G>A	p.Ser872=
Benign	Chr12:g.88508967A>C	*CEP290*	non-canonical splicing	NM_025114.3:c.1825-8T>G	p.?
Benign	Chr12:g.88512301C>T	*CEP290*	missense	NM_025114.3:c.1670G>A	p.Arg557His
Benign	Chr12:g.88514835T>C	*CEP290*	missense	NM_025114.3:c.1298A>G	p.Asp433Gly
Benign	Chr12:g.88519104G>C	*CEP290*	missense	NM_025114.3:c.1108C>G	p.Gln370Glu

Clasification	Genomic-level	Gene	Variant Type	cDNA	Protein
Benign	Chr12:g.88519133C>T	*CEP290*	missense	NM_025114.3:c.1079G>A	p.Arg360Gln
Benign	Chr12:g.88523652G>A	*CEP290*	missense	NM_025114.3:c.671C>T	p.Thr224Ile
Benign	Chr12:g.88525004del	*CEP290*	non-canonical splicing	NM_025114.3:c.442-9del	p.?
Benign	Chr2:g.182403834G>A	*CERKL*	missense	NM_001030311.2:c.1601C>T	p.Ser534Leu
Benign	Chr2:g.182423284C>T	*CERKL*	non-canonical splicing	NM_001030311.2:c.898+9G>A	p.?
Benign	Chr2:g.182521707C>T	*CERKL*	synonymous	NM_001030311.2:c.27G>A	p.Arg9=
Benign	Chr1:g.196643027T>C	*CFH*	synonymous	NM_000186.3:c.285T>C	p.Thr95=
Benign	Chr1:g.196684768A>G	*CFH*	missense	NM_000186.3:c.1565A>G	p.Asp522Gly
Benign	Chr1:g.196709880A>G	*CFH*	missense	NM_000186.3:c.2914A>G	p.Lys972Glu
Benign	Chr1:g.196712598T>C	*CFH*	synonymous	NM_000186.3:c.3150T>C	p.Asn1050=
Benign	ChrX:g.85156079G>A	*CHM*	non-canonical splicing	NM_000390.3:c.1349+10C>T	p.?
Benign	ChrX:g.85156166A>G	*CHM*	synonymous	NM_000390.3:c.1272T>C	p.Gly424=
Benign	ChrX:g.85213928G>T	*CHM*	synonymous	NM_000390.3:c.757C>A	p.Arg253=
Benign	ChrX:g.85302508T>C	*CHM*	missense	NM_000390.3:c.29A>G	p.Asp10Gly
Benign	Chr15:g.78398100T>G	*CIB2*	missense	NM_001301224.1:c.538A>C	p.Lys180Gln
Benign	Chr15:g.78401685C>T	*CIB2*	missense	NM_001301224.1:c.253G>A	p.Glu85Lys
Benign	Chr16:g.28488924C>T	*CLN3*	synonymous	NM_000086.2:c.1230G>A	p.Ala410=
Benign	Chr16:g.28489096C>T	*CLN3*	missense	NM_000086.2:c.1159G>A	p.Ala387Thr
Benign	Chr16:g.28493803C>G	*CLN3*	missense	NM_000086.2:c.901G>C	p.Gly301Arg
Benign	Chr16:g.28502814C>T	*CLN3*	synonymous	NM_000086.2:c.114G>A	p.Ala38=
Benign	Chr3:g.150690413A>C	*CLRN1*	missense	NM_001195794.1:c.83T>G	p.Leu28Trp
Benign	Chr4:g.47938732A>G	*CNGA1*	synonymous	NM_001142564.1:c.1986T>C	p.Thr662=
Benign	Chr4:g.47939137A>G	*CNGA1*	synonymous	NM_001142564.1:c.1581T>C	p.Pro527=

Clasification	Genomic-level	Gene	Variant Type	cDNA	Protein
Benign	Chr4:g.47939360A>G	*CNGA1*	missense	NM_001142564.1:c.1358T>C	p.Ile453Thr
Benign	Chr4:g.47939441G>A	*CNGA1*	missense	NM_001142564.1:c.1277C>T	p.Thr426Ile
Benign	Chr4:g.47944120A>G	*CNGA1*	synonymous	NM_001142564.1:c.702T>C	p.Tyr234=
Benign	Chr4:g.47945274C>T	*CNGA1*	missense	NM_001142564.1:c.580G>A	p.Asp194Asn
Benign	Chr4:g.47945275G>A	*CNGA1*	synonymous	NM_001142564.1:c.579C>T	p.Asn193=
Benign	Chr4:g.47951876A>G	*CNGA1*	synonymous	NM_001142564.1:c.480T>C	p.Asn160=
Benign	Chr2:g.99013202C>T	*CNGA3*	synonymous	NM_001298.2:c.1569C>T	p.Asn523=
Benign	Chr2:g.99013504A>G	*CNGA3*	missense	NM_001298.2:c.1871A>G	p.Lys624Arg
Benign	Chr16:g.57918095C>T	*CNGB1*	synonymous	NM_001297.4:c.3729G>A	p.Pro1243=
Benign	Chr16:g.57918264C>T	*CNGB1*	missense	NM_001297.4:c.3560G>A	p.Arg1187Gln
Benign	Chr16:g.57921913G>A	*CNGB1*	missense	NM_001297.4:c.3308C>T	p.Ala1103Val
Benign	Chr16:g.57931776C>G	*CNGB1*	missense	NM_001297.4:c.3019G>C	p.Val1007Leu
Benign	Chr16:g.57945776G>T	*CNGB1*	synonymous	NM_001297.4:c.2373C>A	p.Val791=
Benign	Chr16:g.57946905G>A	*CNGB1*	non-canonical splicing	NM_001297.4:c.2305-7C>T	p.?
Benign	Chr16:g.57949163C>T	*CNGB1*	missense	NM_001297.4:c.2294G>A	p.Arg765His
Benign	Chr16:g.57949191C>T	*CNGB1*	missense	NM_001297.4:c.2266G>A	p.Gly756Ser
Benign	Chr8:g.87588042G>C	*CNGB3*	missense	NM_019098.4:c.2420C>G	p.Ala807Gly
Benign	Chr8:g.87588261C>T	*CNGB3*	missense	NM_019098.4:c.2201G>A	p.Gly734Glu
Benign	Chr8:g.87588262C>G	*CNGB3*	missense	NM_019098.4:c.2200G>C	p.Gly734Arg
Benign	Chr8:g.87590922C>G	*CNGB3*	missense	NM_019098.4:c.2098G>C	p.Ala700Pro
Benign	Chr8:g.87679266C>T	*CNGB3*	missense	NM_019098.4:c.739G>A	p.Ala247Thr
Benign	Chr8:g.87738778C>T	*CNGB3*	missense	NM_019098.4:c.319G>A	p.Gly107Arg
Benign	Chr13:g.40298637_40298638del	*COG6*	non-canonical splicing	NM_020751.2:c.1693-8_1693-7del	p.?
Benign	Chr1:g.103540265G>T	*COL11A1*	missense	NM_080629.2:c.560C>A	p.Thr187Lys

Clasification	Genomic-level	Gene	Variant Type	cDNA	Protein
Benign	Chr12:g.48368612G>T	*COL2A1*	missense	NM_001844.5:c.3920C>A	p.Thr1307Asn
Benign	Chr12:g.48372456C>T	*COL2A1*	missense	NM_001844.5:c.2819G>A	p.Arg940Gln
Benign	Chr12:g.48381439G>A	*COL2A1*	synonymous	NM_001844.5:c.1176C>T	p.Arg392=
Benign	Chr12:g.48388220G>A	*COL2A1*	missense	NM_001844.5:c.803C>T	p.Pro268Leu
Benign	Chr1:g.197297616C>G	*CRB1*	missense	NM_201253.2:c.135C>G	p.Cys45Trp
Benign	Chr1:g.197316487C>T	*CRB1*	missense	NM_201253.2:c.866C>T	p.Thr289Met
Benign	Chr1:g.197325986C>A	*CRB1*	synonymous	NM_201253.2:c.1014C>A	p.Ile338=
Benign	Chr1:g.197390318G>A	*CRB1*	missense	NM_201253.2:c.1360G>A	p.Gly454Arg
Benign	Chr1:g.197396762C>T	*CRB1*	synonymous	NM_201253.2:c.2307C>T	p.Arg769=
Benign	Chr1:g.197396943A>T	*CRB1*	missense	NM_201253.2:c.2488A>T	p.Ile830Phe
Benign	Chr1:g.197407783C>T	*CRB1*	missense	NM_201253.2:c.3856C>T	p.Arg1286Trp
Benign	Chr1:g.197446848G>A	*CRB1*	missense	NM_201253.2:c.4060G>A	p.Ala1354Thr
Benign	Chr19:g.48337728C>G	*CRX*	missense	NM_000554.4:c.28C>G	p.His10Asp
Benign	Chr19:g.48339602C>T	*CRX*	missense	NM_000554.4:c.203C>T	p.Ala68Val
Benign	Chr19:g.48339661T>G	*CRX*	non-canonical splicing	NM_000554.4:c.252+10T>G	p.?
Benign	Chr19:g.48342693G>A	*CRX*	synonymous	NM_000554.4:c.369G>A	p.Thr123=
Benign	Chr19:g.48342873G>A	*CRX*	synonymous	NM_000554.4:c.549G>A	p.Gly183=
Benign	Chr19:g.48342896A>C	*CRX*	missense	NM_000554.4:c.572A>C	p.Tyr191Ser
Benign	Chr19:g.48343114G>A	*CRX*	missense	NM_000554.4:c.790G>A	p.Val264Met
Benign	Chr8:g.67988721A>G	*CSPP1*	missense	NM_024790.6:c.212A>G	p.Lys71Arg
Benign	Chr4:g.187130349G>A	*CYP4V2*	missense	NM_207352.3:c.1328G>A	p.Arg443Gln
Benign	ChrX:g.31950276T>C	*DMD*	missense	NM_004006.2:c.6683A>G	p.Glu2228Gly
Benign	ChrX:g.32361275C>T	*DMD*	synonymous	NM_004006.2:c.5715G>A	p.Glu1905=
Benign	ChrX:g.32383152C>A	*DMD*	missense	NM_004006.2:c.5010G>T	p.Trp1670Cys
Benign	ChrX:g.32481564A>G	*DMD*	missense	NM_004006.2:c.3424T>C	p.Cys1142Arg

Clasification	Genomic-level	Gene	Variant Type	cDNA	Protein
Benign	ChrX:g.32717327T>C	*DMD*	missense	NM_004006.2:c.733A>G	p.Ile245Val
Benign	ChrX:g.32862944G>A	*DMD*	synonymous	NM_004006.2:c.220C>T	p.Leu74=
Benign	Chr2:g.56103787T>C	*EFEMP1*	missense	NM_001039348.2:c.851A>G	p.Glu284Gly
Benign	Chr2:g.56145020G>A	*EFEMP1*	synonymous	NM_001039348.2:c.297C>T	p.Thr99=
Benign	Chr2:g.56149558G>A	*EFEMP1*	synonymous	NM_001039348.2:c.18C>T	p.Phe6=
Benign	Chr10:g.50678884T>G	*ERCC6*	missense	NM_000124.3:c.3122A>C	p.Gln1041Pro
Benign	Chr10:g.50682162G>A	*ERCC6*	missense	NM_000124.3:c.2509C>T	p.Arg837Cys
Benign	Chr10:g.50690906G>A	*ERCC6*	missense	NM_000124.3:c.1996C>T	p.Arg666Cys
Benign	Chr10:g.50732202T>G	*ERCC6*	missense	NM_000124.3:c.1274A>C	p.Asp425Ala
Benign	Chr10:g.50732522G>A	*ERCC6*	synonymous	NM_000124.3:c.954C>T	p.Ala318=
Benign	Chr6:g.64430642C>T	*EYS*	synonymous	NM_001292009.1:c.9348G>A	p.Lys3116=
Benign	Chr6:g.64431463A>G	*EYS*	missense	NM_001292009.1:c.8527T>C	p.Tyr2843His
Benign	Chr6:g.64436488A>G	*EYS*	synonymous	NM_001292009.1:c.8220T>C	p.His2740=
Benign	Chr6:g.64487976G>C	*EYS*	synonymous	NM_001292009.1:c.7821C>G	p.Gly2607=
Benign	Chr6:g.64498113G>A	*EYS*	synonymous	NM_001292009.1:c.7608C>T	p.Ile2536=
Benign	Chr6:g.64499022C>T	*EYS*	missense	NM_001292009.1:c.7507G>A	p.Glu2503Lys
Benign	Chr6:g.64574085T>C	*EYS*	missense	NM_001292009.1:c.7222A>G	p.Thr2408Ala
Benign	Chr6:g.64776324G>A	*EYS*	missense	NM_001292009.1:c.6632C>T	p.Ser2211Leu
Benign	Chr6:g.65016935A>T	*EYS*	missense	NM_001292009.1:c.6119T>A	p.Val2040Asp
Benign	Chr6:g.65300769G>A	*EYS*	missense	NM_001292009.1:c.4991C>T	p.Thr1664Ile
Benign	Chr6:g.65300869G>A	*EYS*	missense	NM_001292009.1:c.4891C>T	p.Pro1631Ser
Benign	Chr6:g.65301652G>A	*EYS*	missense	NM_001292009.1:c.4108C>T	p.His1370Tyr
Benign	Chr6:g.65303192A>G	*EYS*	missense	NM_001292009.1:c.3695T>C	p.Ile1232Thr
Benign	Chr6:g.65622473G>A	*EYS*	missense	NM_001292009.1:c.2545C>T	p.Arg849Cys
Benign	Chr6:g.66005927C>T	*EYS*	missense	NM_001292009.1:c.1852G>A	p.Gly618Ser

Clasification	Genomic-level	Gene	Variant Type	cDNA	Protein
Benign	Chr6:g.66045049C>T	*EYS*	non-canonical splicing	NM_001292009.1:c.1600-10G>A	p.?
Benign	Chr6:g.66063461T>G	*EYS*	missense	NM_001292009.1:c.1349A>C	p.Asn450Thr
Benign	Chr2:g.62053677A>G	*FAM161A*	synonymous	NM_001201543.1:c.2064T>C	p.Ile688=
Benign	Chr14:g.92357580C>T	*FBLN5*	missense	NM_006329.3:c.604G>A	p.Gly202Arg
Benign	Chr14:g.92403446A>G	*FBLN5*	missense	NM_006329.3:c.224T>C	p.Val75Ala
Benign	Chr1:g.213032386C>A	*FLVCR1*	missense	NM_014053.3:c.592C>A	p.His198Asn
Benign	Chr1:g.213032389C>T	*FLVCR1*	missense	NM_014053.3:c.595C>T	p.Leu199Phe
Benign	Chr1:g.213032541T>C	*FLVCR1*	non-canonical splicing	NM_014053.3:c.738+9T>C	p.?
Benign	Chr17:g.79495766C>T	*FSCN2*	missense	NM_001077182.2:c.209C>T	p.Ser70Leu
Benign	Chr17:g.79495973C>T	*FSCN2*	missense	NM_001077182.2:c.416C>T	p.Pro139Leu
Benign	Chr17:g.79496142C>T	*FSCN2*	synonymous	NM_001077182.2:c.585C>T	p.Arg195=
Benign	Chr17:g.79496248C>T	*FSCN2*	missense	NM_001077182.2:c.691C>T	p.Pro231Ser
Benign	Chr17:g.79496367C>T	*FSCN2*	synonymous	NM_001077182.2:c.810C>T	p.Tyr270=
Benign	Chr17:g.79503213G>A	*FSCN2*	missense	NM_001077182.2:c.1025G>A	p.Arg342Gln
Benign	Chr17:g.79503772C>T	*FSCN2*	synonymous	NM_001077182.2:c.1302C>T	p.Asp434=
Benign	Chr17:g.79504047T>G	*FSCN2*	missense	NM_001077182.2:c.1492T>G	p.Ser498Ala
Benign	Chr6:g.42130650C>G	*GUCA1A*	non-canonical splicing	NM_000409.4:c.-457-8C>G	p.?
Benign	Chr17:g.7906439C>T	*GUCY2D*	missense	NM_000180.3:c.74C>T	p.Ser25Phe
Benign	Chr17:g.7906494_7906499del	*GUCY2D*	in-frame	NM_000180.3:c.129_134del	p.Leu44_Leu45del
Benign	Chr17:g.7906854T>G	*GUCY2D*	synonymous	NM_000180.3:c.489T>G	p.Pro163=
Benign	Chr17:g.7907369C>T	*GUCY2D*	synonymous	NM_000180.3:c.921C>T	p.Ala307=
Benign	Chr17:g.7907469C>A	*GUCY2D*	missense	NM_000180.3:c.1021C>A	p.Gln341Lys
Benign	Chr17:g.7909801C>T	*GUCY2D*	missense	NM_000180.3:c.1147C>T	p.Arg383Trp

Clasification	Genomic-level	Gene	Variant Type	cDNA	Protein
Benign	Chr17:g.7909924C>T	*GUCY2D*	missense	NM_000180.3:c.1270C>T	p.Arg424Trp
Benign	Chr17:g.7910817C>T	*GUCY2D*	missense	NM_000180.3:c.1537C>T	p.Leu513Phe
Benign	Chr17:g.7910832G>A	*GUCY2D*	missense	NM_000180.3:c.1552G>A	p.Gly518Ser
Benign	Chr17:g.7915870C>T	*GUCY2D*	missense	NM_000180.3:c.2059C>T	p.His687Tyr
Benign	Chr5:g.140056256C>T	*HARS*	missense	NM_002109.5:c.1177G>A	p.Val393Met
Benign	Chr10:g.71128330C>T	*HK1*	synonymous	NM_001322365.1:c.639C>T	p.Ser213=
Benign	Chr10:g.71139779G>A	*HK1*	missense	NM_001322365.1:c.1298G>A	p.Arg433His
Benign	Chr10:g.71152072C>T	*HK1*	non-canonical splicing	NM_001322365.1:c.2324+8C>T	p.?
Benign	Chr1:g.185984522C>A	*HMCN1*	missense	NM_031935.2:c.4862C>A	p.Ala1621Asp
Benign	Chr1:g.186064582C>T	*HMCN1*	missense	NM_031935.2:c.10502C>T	p.Ser3501Leu
Benign	Chr10:g.124248943C>T	*HTRA1*	missense	NM_002775.4:c.578C>T	p.Pro193Leu
Benign	Chr16:g.1616239T>C	*IFT140*	synonymous	NM_014714.3:c.1824A>G	p.Thr608=
Benign	Chr7:g.128034606T>C	*IMPDH1*	missense	NM_000883.3:c.1598A>G	p.Gln533Arg
Benign	Chr7:g.128037009T>C	*IMPDH1*	missense	NM_000883.3:c.1142A>G	p.His381Arg
Benign	Chr7:g.128040477C>T	*IMPDH1*	synonymous	NM_000883.3:c.696G>A	p.Thr232=
Benign	Chr7:g.128041130G>A	*IMPDH1*	missense	NM_000883.3:c.443C>T	p.Thr148Met
Benign	Chr20:g.10621495G>C	*JAG1*	missense	NM_000214.3:c.3135C>G	p.Asn1045Lys
Benign	Chr20:g.10625624C>T	*JAG1*	missense	NM_000214.3:c.2231G>A	p.Arg744Gln
Benign	Chr20:g.10632876A>G	*JAG1*	synonymous	NM_000214.3:c.909T>C	p.His303=
Benign	Chr10:g.94353214G>C	*KIF11*	canonical splicing	NM_004523.3:c.77+5G>C	r.spl?
Benign	Chr18:g.7011295T>G	*LAMA1*	non-canonical splicing	NM_005559.3:c.3687+4A>C	r.spl?
Benign	Chr6:g.80197180T>C	*LCA5*	synonymous	NM_001122769.2:c.1635A>G	p.Pro545=
Benign	Chr6:g.80198875A>C	*LCA5*	missense	NM_001122769.2:c.1157T>G	p.Met386Arg

Clasification	Genomic-level	Gene	Variant Type	cDNA	Protein
Benign	Chr6:g.80201320T>C	*LCA5*	synonymous	NM_001122769.2:c.1083A>G	p.Pro361=
Benign	Chr6:g.80223341C>T	*LCA5*	missense	NM_001122769.2:c.308G>A	p.Arg103Gln
Benign	Chr4:g.110772990C>G	*LRIT3*	synonymous	NM_198506.4:c.447C>G	p.Leu149=
Benign	Chr11:g.68080234_68080242del	*LRP5*	in-frame	NM_002335.4:c.52_60del	p.Leu18_Leu20del
Benign	Chr2:g.112687011A>C	*MERTK*	missense	NM_006343.2:c.376A>C	p.Thr126Pro
Benign	Chr2:g.112705097G>A	*MERTK*	missense	NM_006343.2:c.710G>A	p.Arg237His
Benign	Chr2:g.112725832A>G	*MERTK*	non-canonical splicing	NM_006343.2:c.960+3A>G	r.spl?
Benign	Chr2:g.112779952G>C	*MERTK*	missense	NM_006343.2:c.2467G>C	p.Glu823Gln
Benign	Chr2:g.112785993G>A	*MERTK*	missense	NM_006343.2:c.2552G>A	p.Arg851Lys
Benign	Chr1:g.12057461C>T	*MFN2*	synonymous	NM_014874.3:c.582C>T	p.Asp194=
Benign	Chr1:g.12061533G>A	*MFN2*	missense	NM_014874.3:c.892G>A	p.Gly298Arg
Benign	Chr1:g.12064941G>A	*MFN2*	synonymous	NM_014874.3:c.1452G>A	p.Thr484=
Benign	Chr1:g.12066601C>T	*MFN2*	missense	NM_014874.3:c.1723C>T	p.Arg575Cys
Benign	Chr11:g.119210565G>C	*MFRP*	non-canonical splicing	NM_031433.3:c.*1111-7C>G	p.?
Benign	Chr11:g.119211403A>C	*MFRP*	canonical splicing	NM_031433.3:c.*853+2T>G	r.spl
Benign	Chr11:g.119212589A>G	*MFRP*	missense	NM_031433.3:c.1493T>C	p.Val498Ala
Benign	Chr11:g.119213441C>T	*MFRP*	non-canonical splicing	NM_031433.3:c.1256-4G>A	p.?
Benign	Chr11:g.119214644C>T	*MFRP*	missense	NM_031433.3:c.1006G>A	p.Gly336Arg
Benign	Chr11:g.119216210T>C	*MFRP*	synonymous	NM_031433.3:c.561A>G	p.Glu187=
Benign	Chr11:g.119217092G>A	*MFRP*	non-canonical splicing	NM_031433.3:c.55-8C>T	p.?
Benign	Chr4:g.128854212A>G	*MFSD8*	missense	NM_152778.2:c.791T>C	p.Ile264Thr

Clasification	Genomic-level	Gene	Variant Type	cDNA	Protein
Benign	Chr20:g.10386290G>C	*MKKS*	missense	NM_018848.3:c.1318C>G	p.Gln440Glu
Benign	Chr20:g.10393292C>T	*MKKS*	missense	NM_018848.3:c.871G>A	p.Val291Ile
Benign	Chr4:g.100503095G>A	*MTTP*	missense	NM_001300785.1:c.176G>A	p.Arg59Gln
Benign	Chr4:g.100542308G>C	*MTTP*	missense	NM_001300785.1:c.2514G>C	p.Leu838Phe
Benign	Chr12:g.110019365G>A	*MVK*	non-canonical splicing	NM_000431.3:c.527+10G>A	p.?
Benign	Chr11:g.76867711A>G	*MYO7A*	missense	NM_000260.3:c.476A>G	p.Glu159Gly
Benign	Chr11:g.76869352G>A	*MYO7A*	synonymous	NM_000260.3:c.879G>A	p.Val293=
Benign	Chr11:g.76872050T>C	*MYO7A*	missense	NM_000260.3:c.1232T>C	p.Val411Ala
Benign	Chr11:g.76872106C>T	*MYO7A*	missense	NM_000260.3:c.1288C>T	p.Arg430Cys
Benign	Chr11:g.76883842C>T	*MYO7A*	missense	NM_000260.3:c.1846C>T	p.Arg616Trp
Benign	Chr11:g.76885923G>A	*MYO7A*	missense	NM_000260.3:c.2057G>A	p.Arg686His
Benign	Chr11:g.76886437G>A	*MYO7A*	missense	NM_000260.3:c.2114G>A	p.Cys705Tyr
Benign	Chr11:g.76890117C>T	*MYO7A*	missense	NM_000260.3:c.2309C>T	p.Ala770Val
Benign	Chr11:g.76890901C>A	*MYO7A*	missense	NM_000260.3:c.2488C>A	p.Arg830Ser
Benign	Chr11:g.76891451G>A	*MYO7A*	missense	NM_000260.3:c.2618G>A	p.Arg873Gln
Benign	Chr11:g.76892490G>A	*MYO7A*	missense	NM_000260.3:c.2759G>A	p.Arg920Gln
Benign	Chr11:g.76892617G>C	*MYO7A*	missense	NM_000260.3:c.2886G>C	p.Gln962His
Benign	Chr11:g.76903227G>T	*MYO7A*	synonymous	NM_000260.3:c.4056G>T	p.Thr1352=
Benign	Chr11:g.76908650C>T	*MYO7A*	non-canonical splicing	NM_000260.3:c.4441+7C>T	p.?
Benign	Chr11:g.76912557G>A	*MYO7A*	synonymous	NM_000260.3:c.4917G>A	p.Thr1639=
Benign	Chr11:g.76912577C>T	*MYO7A*	missense	NM_000260.3:c.4937C>T	p.Ser1646Leu
Benign	Chr11:g.76912623C>T	*MYO7A*	synonymous	NM_000260.3:c.4983C>T	p.Asp1661=
Benign	Chr11:g.76915212C>T	*MYO7A*	synonymous	NM_000260.3:c.5418C>T	p.Ala1806=
Benign	Chr11:g.76916520C>A	*MYO7A*	synonymous	NM_000260.3:c.5494C>A	p.Arg1832=

Clasification	Genomic-level	Gene	Variant Type	cDNA	Protein
Benign	Chr11:g.76916669C>T	*MYO7A*	non-canonical splicing	NM_000260.3:c.5636+7C>T	p.?
Benign	Chr11:g.76922875G>A	*MYO7A*	missense	NM_000260.3:c.6247G>A	p.Ala2083Thr
Benign	Chr11:g.76925033G>A	*MYO7A*	non-canonical splicing	NM_000260.3:c.6558+9G>A	p.?
Benign	Chr2:g.182542838G>T	*NEUROD1*	missense	NM_002500.4:c.750C>A	p.Ser250Arg
Benign	Chr2:g.182542865G>C	*NEUROD1*	missense	NM_002500.4:c.723C>G	p.His241Gln
Benign	Chr1:g.10032254C>G	*NMNAT1*	non-canonical splicing	NM_001297778.1:c.115+8C>G	p.?
Benign	Chr1:g.10042362T>C	*NMNAT1*	missense	NM_001297778.1:c.443T>C	p.Val148Ala
Benign	Chr2:g.110889309C>T	*NPHP1*	missense	NM_000272.3:c.1757G>A	p.Arg586Gln
Benign	Chr3:g.132437948A>G	*NPHP3*	missense	NM_153240.5:c.560T>C	p.Leu187Pro
Benign	Chr3:g.132441011C>G	*NPHP3*	synonymous	NM_153240.5:c.189G>C	p.Gly63=
Benign	Chr1:g.5923976G>A	*NPHP4*	synonymous	NM_015102.4:c.4114C>T	p.Leu1372=
Benign	Chr1:g.5927838T>C	*NPHP4*	missense	NM_015102.4:c.3434A>G	p.Lys1145Arg
Benign	Chr1:g.5934594G>A	*NPHP4*	synonymous	NM_015102.4:c.3168C>T	p.His1056=
Benign	Chr1:g.5934707C>T	*NPHP4*	missense	NM_015102.4:c.3055G>A	p.Asp1019Asn
Benign	Chr1:g.5935014G>A	*NPHP4*	synonymous	NM_015102.4:c.2964C>T	p.Ala988=
Benign	Chr1:g.5947503C>T	*NPHP4*	synonymous	NM_015102.4:c.2328G>A	p.Pro776=
Benign	Chr1:g.5964781G>A	*NPHP4*	missense	NM_015102.4:c.2039C>T	p.Thr680Met
Benign	Chr1:g.5965684C>T	*NPHP4*	non-canonical splicing	NM_015102.4:c.1763+8G>A	p.?
Benign	Chr1:g.5987711G>A	*NPHP4*	missense	NM_015102.4:c.1439C>T	p.Ser480Leu
Benign	Chr1:g.5987724G>C	*NPHP4*	missense	NM_015102.4:c.1426C>G	p.Pro476Ala
Benign	Chr1:g.5993350G>T	*NPHP4*	missense	NM_015102.4:c.1159C>A	p.His387Asn
Benign	Chr1:g.6012770T>A	*NPHP4*	missense	NM_015102.4:c.800A>T	p.His267Leu

Clasification	Genomic-level	Gene	Variant Type	cDNA	Protein
Benign	NG_009113.1:g.5932G>A	*NR2E3*	missense	NM_014249.2:c.121G>A	p.Val41Met
Benign	NG_009113.1:g.6951G>A	*NR2E3*	missense	NM_014249.2:c.740G>A	p.Arg247Gln
Benign	NG_009113.1:g.6965A>C	*NR2E3*	non-canonical splicing	NM_014249.2:c.747+7A>C	p.?
Benign	NG_009113.1:g.6967C>T	*NR2E3*	non-canonical splicing	NM_014249.2:c.747+9C>T	p.?
Benign	Chr10:g.126090344G>A	*OAT*	missense	NM_000274.3:c.965C>T	p.Thr322Ile
Benign	Chr10:g.126092359G>A	*OAT*	non-canonical splicing	NM_000274.3:c.771+8C>T	p.?
Benign	ChrX:g.13757042G>A	*OFD1*	non-canonical splicing	NM_003611.2:c.381+9G>A	p.?
Benign	ChrX:g.13779248A>G	*OFD1*	missense	NM_003611.2:c.2305A>G	p.Arg769Gly
Benign	Chr10:g.55581760C>T	*PCDH15*	missense	NM_001142763.1:c.5747G>A	p.Arg1916His
Benign	Chr10:g.55581880G>T	*PCDH15*	missense	NM_001142763.1:c.5627C>A	p.Thr1876Lys
Benign	Chr10:g.55581921G>A	*PCDH15*	synonymous	NM_001142763.1:c.5586C>T	p.Ala1862=
Benign	Chr10:g.55582072G>A	*PCDH15*	missense	NM_001142763.1:c.5435C>T	p.Pro1812Leu
Benign	Chr10:g.55582200_55582208del	*PCDH15*	in-frame	NM_001142763.1:c.5299_5307del	p.Pro1767_Pro1769del
Benign	Chr10:g.55582438A>C	*PCDH15*	missense	NM_001142763.1:c.5069T>G	p.Leu1690Arg
Benign	Chr10:g.55587188A>G	*PCDH15*	synonymous	NM_001142763.1:c.4347T>C	p.Gly1449=
Benign	Chr10:g.55600246G>T	*PCDH15*	missense	NM_001142763.1:c.3832C>A	p.Arg1278Ser
Benign	Chr10:g.55626500C>A	*PCDH15*	missense	NM_001142763.1:c.3634G>T	p.Ala1212Ser
Benign	Chr10:g.55721636C>A	*PCDH15*	missense	NM_001142763.1:c.2900G>T	p.Arg967Leu
Benign	Chr10:g.55780012C>T	*PCDH15*	synonymous	NM_001142763.1:c.2706G>A	p.Glu902=
Benign	Chr10:g.55780122C>T	*PCDH15*	missense	NM_001142763.1:c.2596G>A	p.Val866Met
Benign	Chr10:g.55780140G>A	*PCDH15*	missense	NM_001142763.1:c.2578C>T	p.Arg860Trp
Benign	Chr10:g.55780165G>A	*PCDH15*	synonymous	NM_001142763.1:c.2553C>T	p.Val851=

Clasification	Genomic-level	Gene	Variant Type	cDNA	Protein
Benign	Chr10:g.55782754C>G	*PCDH15*	missense	NM_001142763.1:c.2439G>C	p.Lys813Asn
Benign	Chr10:g.55782767G>T	*PCDH15*	missense	NM_001142763.1:c.2426C>A	p.Thr809Asn
Benign	Chr10:g.55782792C>T	*PCDH15*	missense	NM_001142763.1:c.2401G>A	p.Val801Ile
Benign	Chr10:g.55782957C>T	*PCDH15*	exonic canonical splicing	NM_001142763.1:c.2236G>A	r.spl?
Benign	Chr10:g.55839183T>C	*PCDH15*	missense	NM_001142763.1:c.2014A>G	p.Thr672Ala
Benign	Chr10:g.55892709G>A	*PCDH15*	missense	NM_001142763.1:c.1858C>T	p.Arg620Cys
Benign	Chr10:g.55955627G>A	*PCDH15*	missense	NM_001142763.1:c.1136C>T	p.Pro379Leu
Benign	Chr10:g.56077211A>G	*PCDH15*	non-canonical splicing	NM_001142763.1:c.721-10T>C	p.?
Benign	Chr10:g.56106198T>C	*PCDH15*	missense	NM_001142763.1:c.536A>G	p.Asn179Ser
Benign	Chr5:g.149240540A>G	*PDE6A*	non-canonical splicing	NM_000440.2:c.2507-6T>C	p.?
Benign	Chr5:g.149262992A>G	*PDE6A*	exonic canonical splicing	NM_000440.2:c.2135T>C	r.spl?
Benign	Chr5:g.149278948G>A	*PDE6A*	missense	NM_000440.2:c.1253C>T	p.Thr418Met
Benign	Chr5:g.149310665C>T	*PDE6A*	missense	NM_000440.2:c.784G>A	p.Ala262Thr
Benign	Chr5:g.149323870C>A	*PDE6A*	missense	NM_000440.2:c.367G>T	p.Asp123Tyr
Benign	Chr4:g.619619G>C	*PDE6B*	missense	NM_000283.3:c.204G>C	p.Glu68Asp
Benign	Chr4:g.619635C>T	*PDE6B*	missense	NM_000283.3:c.220C>T	p.Arg74Cys
Benign	Chr4:g.619864A>T	*PDE6B*	missense	NM_000283.3:c.449A>T	p.Asn150Ile
Benign	Chr4:g.647913G>A	*PDE6B*	synonymous	NM_000283.3:c.897G>A	p.Pro299=
Benign	Chr4:g.648675T>C	*PDE6B*	synonymous	NM_000283.3:c.990T>C	p.Ile330=
Benign	Chr4:g.650773C>T	*PDE6B*	synonymous	NM_000283.3:c.1218C>T	p.Asp406=

Clasification	Genomic-level	Gene	Variant Type	cDNA	Protein
Benign	Chr4:g.651288G>A	*PDE6B*	canonical splicing	NM_000283.3:c.1401+5G>A	r.spl?
Benign	Chr4:g.652751C>T	*PDE6B*	missense	NM_000283.3:c.1412C>T	p.Ala471Val
Benign	Chr4:g.657561C>T	*PDE6B*	synonymous	NM_000283.3:c.1923C>T	p.Thr641=
Benign	Chr4:g.657607A>G	*PDE6B*	missense	NM_000283.3:c.1969A>G	p.Ile657Val
Benign	Chr10:g.95372854G>T	*PDE6C*	missense	NM_006204.3:c.372G>T	p.Glu124Asp
Benign	Chr10:g.95385328C>T	*PDE6C*	non-canonical splicing	NM_006204.3:c.865-4C>T	p.?
Benign	Chr10:g.102770396C>A	*PDZD7*	missense	NM_001195263.1:c.2250G>T	p.Trp750Cys
Benign	Chr10:g.102775470G>A	*PDZD7*	missense	NM_001195263.1:c.1672C>T	p.Arg558Trp
Benign	Chr10:g.102778025C>T	*PDZD7*	synonymous	NM_001195263.1:c.1353G>A	p.Lys451=
Benign	Chr10:g.102778802G>C	*PDZD7*	missense	NM_001195263.1:c.1101C>G	p.Asp367Glu
Benign	Chr6:g.137167260G>A	*PEX7*	missense	NM_000288.3:c.467G>A	p.Ser156Asn
Benign	Chr10:g.13320306_13320308dup	*PHYH*	in-frame	NM_001323082.1:c.1016_1018dup	p.Asn339_Leu340insHis
Benign	Chr10:g.13330464C>T	*PHYH*	missense	NM_001323082.1:c.580G>A	p.Ala194Thr
Benign	Chr19:g.7604795T>C	*PNPLA6*	non-canonical splicing	NM_001166111.1:c.441-9T>C	p.?
Benign	Chr19:g.7607772C>T	*PNPLA6*	synonymous	NM_001166111.1:c.1515C>T	p.Phe505=
Benign	Chr19:g.7615983G>T	*PNPLA6*	missense	NM_001166111.1:c.2201G>T	p.Arg734Leu
Benign	Chr1:g.46660248T>C	*POMGNT1*	missense	NM_001243766.1:c.728A>G	p.Asp243Gly
Benign	Chr1:g.46662669C>T	*POMGNT1*	missense	NM_001243766.1:c.208G>A	p.Glu70Lys
Benign	Chr4:g.15982165_15982166insGA	*PROM1*	canonical splicing	NM_006017.2:c.2374-6_2374-5insTC	p.?
Benign	Chr4:g.15985975T>C	*PROM1*	missense	NM_006017.2:c.2284A>G	p.Ser762Gly
Benign	Chr4:g.15992900G>C	*PROM1*	missense	NM_006017.2:c.1928C>G	p.Ala643Gly

Clasification	Genomic-level	Gene	Variant Type	cDNA	Protein
Benign	Chr4:g.16000039T>C	*PROM1*	missense	NM_006017.2:c.1651A>G	p.Lys551Glu
Benign	Chr4:g.16000080T>C	*PROM1*	missense	NM_006017.2:c.1610A>G	p.Asp537Gly
Benign	Chr4:g.16002138G>A	*PROM1*	missense	NM_006017.2:c.1559C>T	p.Thr520Met
Benign	Chr4:g.16008270C>T	*PROM1*	missense	NM_006017.2:c.1345G>A	p.Val449Met
Benign	Chr4:g.16025951T>G	*PROM1*	missense	NM_006017.2:c.661A>C	p.Thr221Pro
Benign	Chr4:g.16026841G>C	*PROM1*	missense	NM_006017.2:c.604C>G	p.Arg202Gly
Benign	Chr4:g.16035123A>G	*PROM1*	missense	NM_006017.2:c.313T>C	p.Tyr105His
Benign	Chr1:g.150305441A>G	*PRPF3*	non-canonical splicing	NM_004698.3:c.508-9A>G	p.?
Benign	Chr1:g.150307457G>A	*PRPF3*	synonymous	NM_004698.3:c.780G>A	p.Glu260=
Benign	Chr1:g.150325416C>T	*PRPF3*	synonymous	NM_004698.3:c.2013C>T	p.Asp671=
Benign	Chr1:g.150325422G>A	*PRPF3*	synonymous	NM_004698.3:c.2019G>A	p.Ala673=
Benign	Chr19:g.54627916G>A	*PRPF31*	missense	NM_015629.3:c.736G>A	p.Ala246Thr
Benign	Chr19:g.54627916G>C	*PRPF31*	missense	NM_015629.3:c.736G>C	p.Ala246Pro
Benign	Chr19:g.54627917C>T	*PRPF31*	missense	NM_015629.3:c.737C>T	p.Ala246Val
Benign	Chr19:g.54632524C>T	*PRPF31*	synonymous	NM_015629.3:c.1239C>T	p.Asn413=
Benign	Chr17:g.1557241C>T	*PRPF8*	synonymous	NM_006445.3:c.6057G>A	p.Pro2019=
Benign	Chr17:g.1564106G>A	*PRPF8*	synonymous	NM_006445.3:c.4524C>T	p.Gly1508=
Benign	Chr17:g.1565000G>A	*PRPF8*	synonymous	NM_006445.3:c.4107C>T	p.Tyr1369=
Benign	Chr17:g.1576820C>T	*PRPF8*	missense	NM_006445.3:c.3488G>A	p.Arg1163Gln
Benign	Chr17:g.1579805G>A	*PRPF8*	synonymous	NM_006445.3:c.2382C>T	p.Tyr794=
Benign	Chr17:g.1582181G>C	*PRPF8*	non-canonical splicing	NM_006445.3:c.1600-6C>G	p.?
Benign	Chr17:g.1582960A>T	*PRPF8*	missense	NM_006445.3:c.1232T>A	p.Phe411Tyr
Benign	Chr17:g.1584325T>C	*PRPF8*	missense	NM_006445.3:c.890A>G	p.Asn297Ser
Benign	Chr6:g.42666170C>T	*PRPH2*	missense	NM_000322.4:c.904G>A	p.Glu302Lys

Clasification	Genomic-level	Gene	Variant Type	cDNA	Protein
Benign	Chr6:g.42672306C>T	*PRPH2*	missense	NM_000322.4:c.625G>A	p.Val209Ile
Benign	Chr6:g.42689824G>A	*PRPH2*	synonymous	NM_000322.4:c.249C>T	p.Tyr83=
Benign	ChrX:g.106893247C>T	*PRPS1*	synonymous	NM_002764.3:c.942C>T	p.Ser314=
Benign	Chr13:g.48878122C>T	*RB1*	missense	NM_000321.2:c.74C>T	p.Pro25Leu
Benign	Chr13:g.48916860C>G	*RB1*	non-canonical splicing	NM_000321.2:c.380+10C>G	p.?
Benign	Chr13:g.48954209T>C	*RB1*	synonymous	NM_000321.2:c.1410T>C	p.Ile470=
Benign	Chr10:g.48382161C>T	*RBP3*	missense	NM_002900.2:c.3488G>A	p.Arg1163Gln
Benign	Chr10:g.48385935C>T	*RBP3*	missense	NM_002900.2:c.3157G>A	p.Gly1053Ser
Benign	Chr10:g.48387887G>T	*RBP3*	missense	NM_002900.2:c.2991C>A	p.His997Gln
Benign	Chr10:g.48387978C>A	*RBP3*	missense	NM_002900.2:c.2900G>T	p.Gly967Val
Benign	Chr10:g.48388136G>A	*RBP3*	synonymous	NM_002900.2:c.2742C>T	p.Pro914=
Benign	Chr10:g.48388381G>A	*RBP3*	missense	NM_002900.2:c.2497C>T	p.Arg833Cys
Benign	Chr10:g.48388561C>T	*RBP3*	missense	NM_002900.2:c.2317G>A	p.Val773Met
Benign	Chr10:g.48388658G>A	*RBP3*	synonymous	NM_002900.2:c.2220C>T	p.Pro740=
Benign	Chr10:g.48389072G>A	*RBP3*	synonymous	NM_002900.2:c.1806C>T	p.His602=
Benign	Chr10:g.48389796G>A	*RBP3*	missense	NM_002900.2:c.1082C>T	p.Thr361Met
Benign	Chr10:g.48390078C>T	*RBP3*	missense	NM_002900.2:c.800G>A	p.Arg267Gln
Benign	Chr10:g.48390627T>G	*RBP3*	missense	NM_002900.2:c.251A>C	p.Asp84Ala
Benign	Chr10:g.48390715C>A	*RBP3*	missense	NM_002900.2:c.163G>T	p.Ala55Ser
Benign	Chr1:g.211652648C>T	*RD3*	synonymous	NM_001164688.1:c.318G>A	p.Glu106=
Benign	Chr14:g.68193807C>T	*RDH12*	synonymous	NM_152443.2:c.558C>T	p.His186=
Benign	Chr10:g.86007496C>T	*RGR*	missense	NM_002921.3:c.229C>T	p.Leu77Phe
Benign	Chr10:g.86008759T>C	*RGR*	synonymous	NM_002921.3:c.330T>C	p.Ser110=
Benign	Chr10:g.86012638C>A	*RGR*	synonymous	NM_002921.3:c.396C>A	p.Ala132=
Benign	Chr10:g.86018269C>A	*RGR*	synonymous	NM_002921.3:c.762C>A	p.Pro254=

Clasification	Genomic-level	Gene	Variant Type	cDNA	Protein
Benign	Chr3:g.129247720C>T	*RHO*	synonymous	NM_000539.3:c.144C>T	p.Ile48=
Benign	Chr3:g.129249801C>T	*RHO*	synonymous	NM_000539.3:c.444C>T	p.Phe148=
Benign	Chr3:g.129249802G>T	*RHO*	missense	NM_000539.3:c.445G>T	p.Gly149Trp
Benign	Chr3:g.129249837C>A	*RHO*	synonymous	NM_000539.3:c.480C>A	p.Thr160=
Benign	Chr3:g.129251532C>G	*RHO*	missense	NM_000539.3:c.853C>G	p.Pro285Ala
Benign	Chr15:g.89753546G>C	*RLBP1*	synonymous	NM_000326.4:c.924C>G	p.Pro308=
Benign	Chr15:g.89754967G>C	*RLBP1*	non-canonical splicing	NM_000326.4:c.684+7C>G	p.?
Benign	Chr15:g.89755011C>T	*RLBP1*	missense	NM_000326.4:c.647G>A	p.Arg216Gln
Benign	Chr11:g.62381013G>A	*ROM1*	missense	NM_000327.3:c.260G>A	p.Ser87Asn
Benign	Chr11:g.62381762T>A	*ROM1*	missense	NM_000327.3:c.623T>A	p.Leu208Gln
Benign	Chr8:g.55533537C>A	*RP1*	missense	NM_006269.1:c.11C>A	p.Thr4Asn
Benign	Chr8:g.55533597C>G	*RP1*	missense	NM_006269.1:c.71C>G	p.Pro24Arg
Benign	Chr8:g.55533754C>T	*RP1*	synonymous	NM_006269.1:c.228C>T	p.Leu76=
Benign	Chr8:g.55537350A>G	*RP1*	missense	NM_006269.1:c.908A>G	p.Glu303Gly
Benign	Chr8:g.55541005C>T	*RP1*	synonymous	NM_006269.1:c.4563C>T	p.Asn1521=
Benign	Chr8:g.55541397G>A	*RP1*	missense	NM_006269.1:c.4955G>A	p.Arg1652His
Benign	Chr8:g.55541620T>C	*RP1*	synonymous	NM_006269.1:c.5178T>C	p.Asn1726=
Benign	Chr8:g.55541745A>G	*RP1*	missense	NM_006269.1:c.5303A>G	p.Asp1768Gly
Benign	Chr8:g.55542066G>C	*RP1*	missense	NM_006269.1:c.5624G>C	p.Gly1875Ala
Benign	Chr8:g.55542484G>A	*RP1*	synonymous	NM_006269.1:c.6042G>A	p.Gly2014=
Benign	Chr8:g.55542640_55542642del	*RP1*	in-frame	NM_006269.1:c.6198_6200del	p.Asp2066del
Benign	Chr8:g.10464449C>G	*RP1L1*	missense	NM_178857.5:c.7159G>C	p.Val2387Leu
Benign	Chr8:g.10464767G>C	*RP1L1*	missense	NM_178857.5:c.6841C>G	p.Pro2281Ala
Benign	Chr8:g.10465140C>A	*RP1L1*	missense	NM_178857.5:c.6468G>T	p.Glu2156Asp
Benign	Chr8:g.10465317T>A	*RP1L1*	missense	NM_178857.5:c.6291A>T	p.Glu2097Asp

Clasification	Genomic-level	Gene	Variant Type	cDNA	Protein
Benign	Chr8:g.10465372_10465461del	*RP1L1*	in-frame	NM_178857.5:c.6147_6236del	p.Gln2049_Ala2078del
Benign	Chr8:g.10465460G>A	*RP1L1*	missense	NM_178857.5:c.6148C>T	p.Pro2050Ser
Benign	Chr8:g.10465590T>A	*RP1L1*	missense	NM_178857.5:c.6018A>T	p.Glu2006Asp
Benign	Chr8:g.10465743C>A	*RP1L1*	missense	NM_178857.5:c.5865G>T	p.Gln1955His
Benign	Chr8:g.10465751G>C	*RP1L1*	missense	NM_178857.5:c.5857C>G	p.Gln1953Glu
Benign	Chr8:g.10465770C>A	*RP1L1*	synonymous	NM_178857.5:c.5838G>T	p.Ala1946=
Benign	Chr8:g.10465863G>T	*RP1L1*	synonymous	NM_178857.5:c.5745C>A	p.Ala1915=
Benign	Chr8:g.10466839G>T	*RP1L1*	missense	NM_178857.5:c.4769C>A	p.Pro1590His
Benign	Chr8:g.10467271C>T	*RP1L1*	missense	NM_178857.5:c.4337G>A	p.Gly1446Asp
Benign	Chr8:g.10468095G>T	*RP1L1*	synonymous	NM_178857.5:c.3513C>A	p.Gly1171=
Benign	Chr8:g.10468709C>T	*RP1L1*	missense	NM_178857.5:c.2899G>A	p.Glu967Lys
Benign	Chr8:g.10469846C>A	*RP1L1*	missense	NM_178857.5:c.1762G>T	p.Asp588Tyr
Benign	Chr8:g.10470318C>A	*RP1L1*	missense	NM_178857.5:c.1290G>T	p.Gln430His
Benign	Chr8:g.10470796G>A	*RP1L1*	missense	NM_178857.5:c.812C>T	p.Thr271Met
Benign	Chr8:g.10480459G>A	*RP1L1*	missense	NM_178857.5:c.253C>T	p.Arg85Trp
Benign	ChrX:g.46696565G>A	*RP2*	synonymous	NM_006915.2:c.30G>A	p.Lys10=
Benign	Chr1:g.68906643G>A	*RPE65*	missense	NM_000329.2:c.536C>T	p.Ala179Val
Benign	Chr1:g.68910277G>A	*RPE65*	synonymous	NM_000329.2:c.432C>T	p.Tyr144=
Benign	ChrX:g.38145180C>T	*RPGR*	synonymous	NM_001034853.1:c.3072G>A	p.Glu1024=
Benign	ChrX:g.38145411C>T	*RPGR*	synonymous	NM_001034853.1:c.2841G>A	p.Glu947=
Benign	ChrX:g.38145646T>C	*RPGR*	missense	NM_001034853.1:c.2606A>G	p.Glu869Gly
Benign	ChrX:g.38145718T>C	*RPGR*	missense	NM_001034853.1:c.2534A>G	p.Glu845Gly
Benign	ChrX:g.38145733_38145753del	*RPGR*	in-frame	NM_001034853.1:c.2499_2519del	p.Glu850_Gly856del
Benign	ChrX:g.38146026_38146049del	*RPGR*	in-frame	NM_001034853.1:c.2203_2226del	p.His735_Glu742del
Benign	ChrX:g.38146049G>T	*RPGR*	missense	NM_001034853.1:c.2203C>A	p.His735Asn
Benign	ChrX:g.38146050C>A	*RPGR*	missense	NM_001034853.1:c.2202G>T	p.Glu734Asp

Clasification	Genomic-level	Gene	Variant Type	cDNA	Protein
Benign	ChrX:g.38156552G>T	*RPGR*	missense	NM_001034853.1:c.1399C>A	p.Gln467Lys
Benign	ChrX:g.38169914C>T	*RPGR*	synonymous	NM_001034853.1:c.732G>A	p.Lys244=
Benign	ChrX:g.38182653G>A	*RPGR*	synonymous	NM_001034853.1:c.153C>T	p.Thr51=
Benign	Chr14:g.21756185T>C	*RPGRIP1*	missense	NM_020366.3:c.50T>C	p.Ile17Thr
Benign	Chr14:g.21769315C>T	*RPGRIP1*	missense	NM_020366.3:c.409C>T	p.Arg137Cys
Benign	Chr14:g.21771487dup	*RPGRIP1*	non-canonical splicing	NM_020366.3:c.588-3dup	p.?
Benign	Chr14:g.21792786A>G	*RPGRIP1*	missense	NM_020366.3:c.1772A>G	p.Lys591Arg
Benign	Chr14:g.21792934C>T	*RPGRIP1*	synonymous	NM_020366.3:c.1920C>T	p.Ala640=
Benign	Chr14:g.21794291G>A	*RPGRIP1*	missense	NM_020366.3:c.2669G>A	p.Arg890Gln
Benign	Chr14:g.21796716G>A	*RPGRIP1*	missense	NM_020366.3:c.3029G>A	p.Arg1010Lys
Benign	Chr14:g.21813279C>G	*RPGRIP1*	missense	NM_020366.3:c.3540C>G	p.Asp1180Glu
Benign	Chr14:g.21813359A>T	*RPGRIP1*	non-canonical splicing	NM_020366.3:c.3617+3A>T	r.spl?
Benign	Chr14:g.21816459A>G	*RPGRIP1*	missense	NM_020366.3:c.3746A>G	p.Asp1249Gly
Benign	Chr16:g.53686647A>G	*RPGRIP1L*	missense	NM_015272.4:c.1952T>C	p.Leu651Pro
Benign	Chr2:g.234217866G>A	*SAG*	missense	NM_000541.4:c.31G>A	p.Glu11Lys
Benign	Chr2:g.234217902G>A	*SAG*	missense	NM_000541.4:c.67G>A	p.Asp23Asn
Benign	Chr2:g.234229468C>T	*SAG*	missense	NM_000541.4:c.374C>T	p.Thr125Met
Benign	Chr2:g.234235804C>A	*SAG*	missense	NM_000541.4:c.473C>A	p.Thr158Lys
Benign	Chr2:g.234238158C>G	*SAG*	missense	NM_000541.4:c.668C>G	p.Pro223Arg
Benign	Chr2:g.234240316C>G	*SAG*	missense	NM_000541.4:c.764C>G	p.Ser255Trp
Benign	Chr2:g.234255472G>A	*SAG*	missense	NM_000541.4:c.1132G>A	p.Val378Ile
Benign	Chr1:g.871143G>A	*SAMD11*	non-canonical splicing	NM_152486.2:c.306-9G>A	p.?
Benign	Chr1:g.874816_874817insT	*SAMD11*	frameshift	NM_152486.2:c.682_683insT	p.Pro228Leufs*227

Clasification	Genomic-level	Gene	Variant Type	cDNA	Protein
Benign	Chr1:g.877874T>G	*SAMD11*	non-canonical splicing	NM_152486.2:c.1064+6T>G	r.spl?
Benign	Chr1:g.877981G>C	*SAMD11*	missense	NM_152486.2:c.1107G>C	p.Glu369Asp
Benign	Chr1:g.878029C>T	*SAMD11*	synonymous	NM_152486.2:c.1155C>T	p.Asn385=
Benign	Chr1:g.878159C>T	*SAMD11*	missense	NM_152486.2:c.1285C>T	p.Arg429Trp
Benign	Chr1:g.879373A>G	*SAMD11*	missense	NM_152486.2:c.1886A>G	p.Glu629Gly
Benign	Chr2:g.96949460C>G	*SNRNP200*	non-canonical splicing	NM_014014.4:c.4585-9G>C	p.?
Benign	Chr2:g.96949605G>T	*SNRNP200*	synonymous	NM_014014.4:c.4530C>A	p.Ser1510=
Benign	Chr2:g.96949752G>A	*SNRNP200*	non-canonical splicing	NM_014014.4:c.4393-10C>T	p.?
Benign	Chr2:g.96955046C>T	*SNRNP200*	synonymous	NM_014014.4:c.3012G>A	p.Leu1004=
Benign	Chr9:g.32541468G>C	*TOPORS*	missense	NM_005802.4:c.3055C>G	p.Gln1019Glu
Benign	Chr9:g.32541998G>T	*TOPORS*	missense	NM_005802.4:c.2525C>A	p.Thr842Asn
Benign	Chr9:g.32542458G>A	*TOPORS*	missense	NM_005802.4:c.2065C>T	p.Arg689Cys
Benign	Chr9:g.32542793G>T	*TOPORS*	missense	NM_005802.4:c.1730C>A	p.Ser577Tyr
Benign	Chr9:g.32542998T>C	*TOPORS*	missense	NM_005802.4:c.1525A>G	p.Lys509Glu
Benign	Chr9:g.32543285T>G	*TOPORS*	missense	NM_005802.4:c.1238A>C	p.Gln413Pro
Benign	Chr9:g.32543709T>C	*TOPORS*	missense	NM_005802.4:c.814A>G	p.Ile272Val
Benign	Chr9:g.32550909G>A	*TOPORS*	missense	NM_005802.4:c.61C>T	p.Pro21Ser
Benign	Chr3:g.3189149C>G	*TRNT1*	missense	NM_182916.2:c.818C>G	p.Ala273Gly
Benign	Chr15:g.31354871C>A	*TRPM1*	missense	NM_001252020.1:c.1051G>T	p.Val351Phe
Benign	Chr8:g.63978511A>G	*TTPA*	synonymous	NM_000370.3:c.504T>C	p.Ala168=
Benign	Chr6:g.35467767C>T	*TULP1*	missense	NM_003322.5:c.1486G>A	p.Ala496Thr
Benign	Chr6:g.35471506G>A	*TULP1*	non-canonical splicing	NM_003322.5:c.1224+8C>T	p.?

Clasification	Genomic-level	Gene	Variant Type	cDNA	Protein
Benign	Chr6:g.35477011C>A	*TULP1*	missense	NM_003322.5:c.797G>T	p.Gly266Val
Benign	Chr6:g.35477073C>T	*TULP1*	synonymous	NM_003322.5:c.735G>A	p.Ala245=
Benign	Chr6:g.35477666C>T	*TULP1*	missense	NM_003322.5:c.539G>A	p.Arg180His
Benign	Chr6:g.35479447G>C	*TULP1*	synonymous	NM_003322.5:c.327C>G	p.Ala109=
Benign	Chr6:g.35479525C>T	*TULP1*	synonymous	NM_003322.5:c.249G>A	p.Ala83=
Benign	Chr17:g.26875677G>C	*UNC119*	synonymous	NM_005148.3:c.267C>G	p.Val89=
Benign	Chr11:g.17517160C>T	*USH1C*	missense	NM_153676.3:c.2611G>A	p.Ala871Thr
Benign	Chr11:g.17517203T>C	*USH1C*	synonymous	NM_153676.3:c.2568A>G	p.Val856=
Benign	Chr11:g.17527386A>G	*USH1C*	synonymous	NM_153676.3:c.2124T>C	p.Ser708=
Benign	Chr11:g.17530922C>T	*USH1C*	missense	NM_153676.3:c.1994G>A	p.Ser665Asn
Benign	Chr11:g.17531094G>A	*USH1C*	missense	NM_153676.3:c.1822C>T	p.Pro608Ser
Benign	Chr11:g.17531110G>A	*USH1C*	synonymous	NM_153676.3:c.1806C>T	p.Pro602=
Benign	Chr11:g.17532052C>T	*USH1C*	missense	NM_153676.3:c.1430G>A	p.Arg477Gln
Benign	Chr11:g.17544995C>T	*USH1C*	missense	NM_153676.3:c.790G>A	p.Val264Ile
Benign	Chr11:g.17547981C>T	*USH1C*	missense	NM_153676.3:c.587G>A	p.Arg196Gln
Benign	Chr11:g.17548863C>T	*USH1C*	missense	NM_153676.3:c.403G>A	p.Val135Ile
Benign	Chr11:g.17553080G>A	*USH1C*	synonymous	NM_153676.3:c.114C>T	p.Asp38=
Benign	Chr17:g.72915582A>C	*USH1G*	missense	NM_173477.4:c.1349T>G	p.Met450Arg
Benign	Chr17:g.72915919C>T	*USH1G*	missense	NM_173477.4:c.1012G>A	p.Gly338Arg
Benign	Chr17:g.72916094G>C	*USH1G*	missense	NM_173477.4:c.837C>G	p.Asp279Glu
Benign	Chr17:g.72916098G>A	*USH1G*	missense	NM_173477.4:c.833C>T	p.Ser278Leu
Benign	Chr17:g.72916417G>C	*USH1G*	missense	NM_173477.4:c.514C>G	p.Arg172Gly
Benign	Chr1:g.215802311A>G	*USH2A*	missense	NM_206933.2:c.15364T>C	p.Cys5122Arg
Benign	Chr1:g.215802339A>G	*USH2A*	synonymous	NM_206933.2:c.15336T>C	p.Pro5112=
Benign	Chr1:g.215820902G>A	*USH2A*	missense	NM_206933.2:c.14753C>T	p.Thr4918Met
Benign	Chr1:g.215821030G>A	*USH2A*	synonymous	NM_206933.2:c.14625C>T	p.Ala4875=

Clasification	Genomic-level	Gene	Variant Type	cDNA	Protein
Benign	Chr1:g.215824137C>A	*USH2A*	missense	NM_206933.2:c.14140G>T	p.Ala4714Ser
Benign	Chr1:g.215844553G>A	*USH2A*	missense	NM_206933.2:c.13894C>T	p.Pro4632Ser
Benign	Chr1:g.215847521T>C	*USH2A*	missense	NM_206933.2:c.13732A>G	p.Lys4578Glu
Benign	Chr1:g.215847622C>T	*USH2A*	missense	NM_206933.2:c.13631G>A	p.Gly4544Asp
Benign	Chr1:g.215848642G>A	*USH2A*	missense	NM_206933.2:c.12611C>T	p.Thr4204Met
Benign	Chr1:g.215848693C>T	*USH2A*	missense	NM_206933.2:c.12560G>A	p.Arg4187His
Benign	Chr1:g.215848695A>G	*USH2A*	synonymous	NM_206933.2:c.12558T>C	p.Ile4186=
Benign	Chr1:g.215848910G>A	*USH2A*	missense	NM_206933.2:c.12343C>T	p.Arg4115Cys
Benign	Chr1:g.215940071T>G	*USH2A*	missense	NM_206933.2:c.10999A>C	p.Thr3667Pro
Benign	Chr1:g.215953228G>A	*USH2A*	synonymous	NM_206933.2:c.10896C>T	p.Leu3632=
Benign	Chr1:g.215972336C>T	*USH2A*	missense	NM_206933.2:c.9871G>A	p.Gly3291Ser
Benign	Chr1:g.215990414A>G	*USH2A*	synonymous	NM_206933.2:c.9495T>C	p.Asp3165=
Benign	Chr1:g.216061854C>A	*USH2A*	missense	NM_206933.2:c.8137G>T	p.Ala2713Ser
Benign	Chr1:g.216062273C>T	*USH2A*	missense	NM_206933.2:c.7718G>A	p.Arg2573His
Benign	Chr1:g.216166366C>T	*USH2A*	synonymous	NM_206933.2:c.6801G>A	p.Pro2267=
Benign	Chr1:g.216166466G>A	*USH2A*	missense	NM_206933.2:c.6701C>T	p.Ala2234Val
Benign	Chr1:g.216172296G>A	*USH2A*	missense	NM_206933.2:c.6590C>T	p.Thr2197Ile
Benign	Chr1:g.216246464G>A	*USH2A*	synonymous	NM_206933.2:c.5751C>T	p.Tyr1917=
Benign	Chr1:g.216246489T>C	*USH2A*	missense	NM_206933.2:c.5726A>G	p.Tyr1909Cys
Benign	Chr1:g.216246603C>T	*USH2A*	missense	NM_206933.2:c.5612G>A	p.Gly1871Asp
Benign	Chr1:g.216246638G>A	*USH2A*	synonymous	NM_206933.2:c.5577C>T	p.Phe1859=
Benign	Chr1:g.216270469G>A	*USH2A*	missense	NM_206933.2:c.4714C>T	p.Leu1572Phe
Benign	Chr1:g.216348663T>A	*USH2A*	missense	NM_206933.2:c.4558A>T	p.Ile1520Phe
Benign	Chr1:g.216419979C>T	*USH2A*	synonymous	NM_206933.2:c.2757G>A	p.Gln919=
Benign	Chr1:g.216420434A>G	*USH2A*	missense	NM_206933.2:c.2302T>C	p.Cys768Arg
Benign	Chr1:g.216420480A>G	*USH2A*	synonymous	NM_206933.2:c.2256T>C	p.His752=

Clasification	Genomic-level	Gene	Variant Type	cDNA	Protein
Benign	Chr1:g.216595504C>T	*USH2A*	missense	NM_206933.2:c.175G>A	p.Gly59Arg
Benign	Chr5:g.82808027C>T	*VCAN*	missense	NM_004385.4:c.854C>T	p.Ala285Val
Benign	Chr5:g.82816359A>C	*VCAN*	missense	NM_004385.4:c.2234A>C	p.Lys745Thr
Benign	Chr5:g.82832829G>A	*VCAN*	missense	NM_004385.4:c.4007G>A	p.Arg1336Gln
Benign	Chr5:g.82833421A>T	*VCAN*	synonymous	NM_004385.4:c.4599A>T	p.Ala1533=
Benign	Chr5:g.82833426A>G	*VCAN*	missense	NM_004385.4:c.4604A>G	p.Glu1535Gly
Benign	Chr5:g.82834946G>A	*VCAN*	missense	NM_004385.4:c.6124G>A	p.Asp2042Asn
Benign	Chr5:g.82835304A>G	*VCAN*	missense	NM_004385.4:c.6482A>G	p.Lys2161Arg
Benign	Chr5:g.82836201T>C	*VCAN*	missense	NM_004385.4:c.7379T>C	p.Val2460Ala
Benign	Chr5:g.82836889G>A	*VCAN*	synonymous	NM_004385.4:c.8067G>A	p.Thr2689=
Benign	Chr5:g.82836911C>T	*VCAN*	missense	NM_004385.4:c.8089C>T	p.Arg2697Cys
Benign	Chr5:g.82837270A>T	*VCAN*	synonymous	NM_004385.4:c.8448A>T	p.Thr2816=
Benign	Chr5:g.82837717A>G	*VCAN*	synonymous	NM_004385.4:c.8895A>G	p.Glu2965=
Benign	Chr5:g.82837741G>T	*VCAN*	missense	NM_004385.4:c.8919G>T	p.Gln2973His
Benign	Chr5:g.82837847G>A	*VCAN*	missense	NM_004385.4:c.9025G>A	p.Glu3009Lys
Benign	Chr5:g.82843891C>G	*VCAN*	missense	NM_004385.4:c.9481C>G	p.Leu3161Val
Benign	Chr5:g.82875967T>G	*VCAN*	missense	NM_004385.4:c.10049T>G	p.Ile3350Ser
Benign	Chr4:g.6292935G>A	*WFS1*	missense	NM_001145853.1:c.472G>A	p.Glu158Lys
Benign	Chr4:g.6293695G>A	*WFS1*	missense	NM_001145853.1:c.683G>A	p.Arg228His
Benign	Chr4:g.6296880G>A	*WFS1*	synonymous	NM_001145853.1:c.825G>A	p.Ala275=
Benign	Chr4:g.6303328G>A	*WFS1*	synonymous	NM_001145853.1:c.1806G>A	p.Ala602=
Benign	Chr4:g.6303551G>A	*WFS1*	missense	NM_001145853.1:c.2029G>A	p.Ala677Thr
Benign	Chr4:g.6303788C>T	*WFS1*	missense	NM_001145853.1:c.2266C>T	p.Arg756Cys
Benign	Chr4:g.6304117C>A	*WFS1*	missense	NM_001145853.1:c.2595C>A	p.His865Gln
Benign	Chr4:g.6304132C>T	*WFS1*	synonymous	NM_001145853.1:c.2610C>T	p.Thr870=
Benign	Chr9:g.117166272G>A	*WHRN*	synonymous	NM_015404.3:c.2322C>T	p.Ser774=

Clasification	Genomic-level	Gene	Variant Type	cDNA	Protein
Benign	Chr9:g.117168668C>T	*WHRN*	missense	NM_015404.3:c.2203G>A	p.Val735Ile
Benign	Chr9:g.117168759C>A	*WHRN*	synonymous	NM_015404.3:c.2112G>T	p.Leu704=
Benign	Chr9:g.117185687G>A	*WHRN*	synonymous	NM_015404.3:c.1533C>T	p.Pro511=
Benign	Chr9:g.117186665A>G	*WHRN*	synonymous	NM_015404.3:c.1365T>C	p.Ser455=
Benign	Chr9:g.117188508G>A	*WHRN*	synonymous	NM_015404.3:c.1149C>T	p.Thr383=
Benign	Chr9:g.117228631C>T	*WHRN*	synonymous	NM_015404.3:c.879G>A	p.Thr293=
Benign	Chr9:g.117266708G>A	*WHRN*	missense	NM_015404.3:c.374C>T	p.Pro125Leu
Benign	Chr9:g.117266747T>G	*WHRN*	missense	NM_015404.3:c.335A>C	p.Glu112Ala
Benign	Chr9:g.117267011G>A	*WHRN*	missense	NM_015404.3:c.71C>T	p.Ala24Val
Benign	Chr9:g.117267047G>A	*WHRN*	missense	NM_015404.3:c.35C>T	p.Ser12Leu
Benign	Chr9:g.117267049G>C	*WHRN*	missense	NM_015404.3:c.33C>G	p.Ser11Arg
Benign	Chr9:g.117267063C>G	*WHRN*	missense	NM_015404.3:c.19G>C	p.Gly7Arg
Benign	Chr11:g.46723022C>G	*ZNF408*	missense	NM_024741.2:c.126C>G	p.Asp42Glu
Benign	Chr11:g.46723141T>G	*ZNF408*	missense	NM_024741.2:c.245T>G	p.Leu82Arg
Benign	Chr11:g.46727333G>A	*ZNF408*	missense	NM_024741.2:c.2083G>A	p.Ala695Thr

The pathogenic variants include the variants classified as likely pathogenic or pathogenic regarding de ACMG criteria and its previously association in clinical database, whereas the benign variants comprise the variants classified as likely benign or benign. The subgroup of splicing variants is composed of the variants tagged as splicing in the list. The human reference genome GRCh37 was employed.

Supplementary Table 2. Predictor combination groups tested during the combinatorial analysis.

A) Combinations of non-splicing predictors from Alamut® Batch v1.11 & CADD v1.6 n=63					
$C_{6,1}$=6	CADD v1.6	$C_{6,3}$=20	MAPP+Grantham+CADD v1.6	$C_{6,4}$=15	PhastCons+MAPP+CADD v1.6+Grantham
	Grantham		PhastCons+Grantham+CADD v1.6		PhastCons+SIFT+Grantham+CADD v1.6
	MAPP		PhastCons+MAPP+CADD v1.6		PhastCons+SIFT+MAPP+CADD v1.6
	PhastCons		PhastCons+MAPP+Grantham		PhastCons+SIFT+MAPP+Grantham
	PhyloP		PhastCons+SIFT+CADD v1.6		PhyloP+MAPP+Grantham+CADD v1.6
	SIFT		PhastCons+SIFT+Grantham		PhyloP+PhastCons+Grantham+CADD v1.6
$C_{6,2}$=15	Grantham+CADD v1.6		PhastCons+SIFT+MAPP		PhyloP+PhastCons+MAPP+CADD v1.6
	MAPP+CADD v1.6		PhyloP+Grantham+CADD v1.6		PhyloP+PhastCons+MAPP+Grantham
	MAPP+Grantham		PhyloP+MAPP+CADD v1.6		PhyloP+PhastCons+SIFT+CADD v1.6
	PhastCons+CADD v1.6		PhyloP+MAPP+Grantham		PhyloP+PhastCons+SIFT+Grantham
	PhastCons+Grantham		PhyloP+PhastCons+CADD v1.6		PhyloP+PhastCons+SIFT+MAPP
	PhastCons+MAPP		PhyloP+PhastCons+Grantham		PhyloP+SIFT+Grantham+CADD v1.6
	PhastCons+SIFT		PhyloP+PhastCons+MAPP		PhyloP+SIFT+MAPP+CADD v1.6
	PhyloP+CADD v1.6		PhyloP+PhastCons+SIFT		PhyloP+SIFT+MAPP+Grantham
	PhyloP+Grantham		PhyloP+SIFT+CADD v1.6		SIFT+MAPP+Grantham+CADD v1.6
	PhyloP+MAPP		PhyloP+SIFT+Grantham	$C_{6,5}$=6	PhastCons+Grantham+SIFT+MAPP+CADD v1.6
	PhyloP+PhastCons		PhyloP+SIFT+MAPP		PhyloP+Grantham+SIFT+MAPP+CADD v1.6
	PhyloP+SIFT		SIFT+Grantham+CADD v1.6		PhyloP+PhastCons+MAPP+Grantham+CADD v1.6
	SIFT+CADD v1.6		SIFT+MAPP+CADD v1.6		PhyloP+PhastCons+SIFT+Grantham+CADD v1.6
	SIFT+Grantham				PhyloP+PhastCons+SIFT+MAPP+CADD v1.6
	SIFT+MAPP		SIFT+MAPP+Grantham		PhyloP+PhastCons+SIFT+MAPP+Grantham
				$C_{6,5}$=1	PhyloP+PhastCons+SIFT+MAPP+Grantham+CADD v1.6

B) Combinations of non-splicing predictors from Bystro Genomics & CADD v1.6 n=15					
$C_{4,1}$=4	CADDv1.3	$C_{4,2}$=6	CADDv1.6+CADDv1.3	$C_{4,3}$=4	CADDv1.3+PhyloP-100way+PhastCons-100way
	CADDv1.6		PhyloP-100way+PhastCons-100wa		CADDv1.6+CADDv1.3+PhastCons-100way
	PhyloP-100way		CADDv1.3+PhastCons-100way		CADDv1.6+CADDv1.3+PhyloP-100way
			CADDv1.3+PhyloP-100way		CADDv1.6+PhyloP-100way+PhastCons-100way
	PhastCons-100way		CADDv1.6+PhasCons	$C_{4,4}$=1	CADDv1.6+CADDv1.3+PhastCons-100way+PhyloP-100way
			CADDv1.6+PhyloP-100way		

C) Combinations of splicing predictors from Alamut® Batch v1.11 & SpliceAI n=31					
$C_{5,1}$=5	SPiCE	$C_{5,3}$=10	SPiCE+SSF+NNS	$C_{5,5}$=1	SPiCE+SSF+NNS+MaxEnt+SpliceAI
	SSF		SPiCE+MaxEnt+NNS		
	MaxEnt		SPiCE+SSF+MaxEnt		
	NNS		SSF+MaxEnt+NNS		
	SpliceAI		SPiCE+SSF+SpliceAI		
$C_{5,2}$=10	SPiCE+SSF		SPiCE+MaxEnt+SpliceAI		
	SPiCE+MaxEnt		SPiCE+NNS+SpliceAI		
	SPiCE+NNS		SSF+MaxEnt+SpliceAI		
	SSF+MaxEnt		NNS+MaxEnt+SpliceAI		
	SSF+NNS		SSF+NNS+SpliceAI		
	NNS+MaxEnt	$C_{5,4}$=5	SPiCE+SSF+NNS+MaxEnt		
	SPiCE+SpliceAI		SPiCE+SpliceAI+NNS+MaxEnt		
	MaxEnt+SpliceAI		SPiCE+SSF+SpliceAI+MaxEnt		
	SSF+SpliceAI		SPiCE+SSF+NNS+SpliceAI		
	NNS+SpliceAI		SSF+NNS+MaxEnt+SpliceAI		

In total, 109 combinations of different tools, distributed in three groups (A-C), were evaluated: (a) Models resulting from combinations of the five non-splicing tools from Alamut® Batch and CADDv1.6 (n=63). (b) Models resulting from the combinations of the three prediction tools from Bystro and CADDv1.6 (n=15). (c) Models resulting from the combinations of the four splicing prediction tools from Alamut® Batch and SpliceAI (n=31).

Supplementary Table 3. Genes prioritized with an associated phenotype according to OMIM database identified in the discovery cohort.

Gene MIM number	Gene	Phenotype MIM number	OMIM Phenotype	Inh.
607462	*ATN1*	618494	Congenital hypotonia, epilepsy, developmental delay, and digital anomalies	AD
		125370	Dentatorubral-pallidoluysian atrophy	AD
108730	*ATP2A1*	601003	Brody myopathy	AR
601090	*FOXC1*	601631	Anterior segment dysgenesis 3, multiple subtypes	AD
		602482	Axenfeld-Rieger syndrome, type 3	AD
600502	*IGHMBP2*	616155	Charcot-Marie-Tooth disease, axonal, type 2S	AR
		604320	Neuronopathy, distal hereditary motor, type VI	AR
615618	*POGLUT1*	617232	Muscular dystrophy, limb-girdle 21	AR
		615696	Dowling-Degos disease 4	AD
600857	*SDHA*	613642	Cardiomyopathy, dilated, 1GG	AR
		252011	Mitochondrial complex II deficiency, nuclear type 1	AR
		619259	Neurodegeneration with ataxia and late-onset optic atrophy	AD
		614165	Paragangliomas 5	AD
604217	*SLC34A2*	265100	Pulmonary alveolar microlithiasis	AR
601460	*SLCO2A1*	614441	Hypertrophic osteoarthropathy, primary, autosomal recessive 2	AR
614427	*TSHZ1*	607842	Aural atresia, congenital	AD
605232	*WNK1*	201300	Neuropathy, hereditary sensory and autonomic, type II	AR
		614492	Pseudohypoaldosteronism, type IIC	AD
613208	*XPC*	278720	Xeroderma pigmentosum, group C	AR

OMIM database accessed in November 2021. Abbreviations: AD, autosomal dominant; AR, autosomal recessive; Inh, Inheritance.

Supplementary Table 4. Comparative of the different variant annotation tools and the *in-silico* predictors used in this study.

Group	Variant annotator	Predictor	Type of prediction	Type of variants with an output	Score used	Literat cutoff	Reference
SPLICING TOOLS	Alamut® Batch v1.11	**SPiCE**	Splicing predictions	Subs, del, dup and ins	Score	≥0.218	Leman R et al.[1]
		**SSF**	Splicing predictions	Subs, del, dup and ins	$\frac{Score_{mut} - Score_{WT}}{Score_{WT}} \cdot 100$	≥5	Moles-Fernández A et al.[2]
		**MaxEnt**	Splicing predictions	Subs, del, dup and ins	$\frac{Score_{mut} - Score_{WT}}{Score_{WT}} \cdot 100$	≥10	Tang R et al.[3]
		**NNS**	Splicing predictions	Subs, del, dup and ins	$\frac{Score_{mut} - Score_{WT}}{Score_{WT}} \cdot 100$	≥5	Tang R et al.[3]
	Ensembl Variant Effect Predictor	**SpliceAI**	Splicing predictions	Subs, del, dup and ins	Maximum score	≥0.5	Jaganathan K et al.[4]
NON-SPLICING TOOLS	Alamut® Batch v1.11	**PhastCons**	Sequence evolutionary conservation	Subs	Score	≥0.4	Nalpathamkalam T et al.[5]
		**PhyloP**	Sequence evolutionary conservation	Subs	Score	≥1.5	Nalpathamkalam T et al.[5]
		**SIFT**	Sequence evolutionary conservation	Missense	1 – Score (Weight)	≤0.05	Ng PC et al.[6]
		**MAPP**	Sequence evolutionary conservation	Missense	1 – Score (p-Value)	≤0.01	Stone EA et al.[7]
		**Grantham**	Biophysical characteristics of amino acid	Missense	Score	≥100	Tavtigian SV et al.[8]
	Bystro genomics	**PhastCons - 100way**	Sequence evolutionary conservation	Subs	Score	≥0.4	Nalpathamkalam T et al.[5]
		**PhyloP-100way**	Sequence evolutionary conservation	Subs	Score	≥1.5	Nalpathamkalam T et al.[5]
		**CADDv1.3**	Integrative tool	Subs	Score (PHRED)	≥15	Mather CA et al.[9]
	Ensembl Variant Effect Predictor	**CADDv1.6**	Integrative tool	Subs, del, dup and ins	Score (PHRED)	≥15	Mather CA et al.[9]

Among the different cutoffs described in the literature we selected the most widely accepted. Del, dup and ins included intronic and exonic variants such as frameshift and in-frame variants. Subs included intronic and exonic variants such as missense variants, nonsense variants, synonymous variants. Abbreviations: Del, deletions; Dup, duplications; Ins, insertions; Subs, substitutions; Literat: Literature.

Supplementary Table 5. Different CFAP20 in vivo and in vitro knock-out and knock-down models showing the type of inactivation and its phenotypic effect.

Model organism	Reference name	*CFAP20* effect	Observed phenotype	Reference
Chlamydomonas reinhardtii	2-20	Null (Del6ins AphVIII gene)	Flagellar beating defects. Loss of ciliary waveforms. Failed to respond to illumination.	Meng D et al.[10]
	RL-11	Null<br>(splice site variant +1G>A)	Slightly longer flagella. Motility defects. Loss of the planar asymmetrical waveform. Trembling	Yanagisawa HA et al.[11]
	dmj1-1 (fap20null)	Null<br>(TOC1 insertion)	Motility defects. Loss of planar control. Trembling	
	dmj1-2	Null (frameshift)	Motility defects. Loss of the planar asymmetrical waveform. Trembling	
	dmj1-3<br>(fap20ΔC)	Reduced protein level. ΔC 20 amino acids (HygR ins)	Mixed. Intermediate beating defects.	
Danio rerio	gtl3-knockdown	Morpholino knockdown gtl3 /FAP20	Developmental defects and shorter and fewer cilia in Kupffer's vesicle. Phenotype consistent with ciliary dysfunction (curved body axis, short somite length, and defective heart-looping orientation).	
Paramecium tetraurelia	RNAi BUG22 a-d.	Depletion of four Bug22p paralogs (a,b,c and d): GSPATG00007012001, GSPATG00016658001, GSPATG00012810001, GSPATG00013199001 using RNAi.	Decrease in swimming speed, inefficient ciliary beating<br>and apparent loss of ciliary rigidity. Cell death after a few days.	Laligné C et al.[12]
Drosophila melanogaster	Bug22<br>$P[Bug22^{KO}]$	Null allele<br>by homologous recombination (Deletion of the CG5343 gene).	Defects in locomotion, climbing activity and gravitaxis. Uncoordination, feed impediments. Defects in wing inflation. Short lifespan. Immotile sperm. Defects in sperm individualization. Abnormal tubulin polyglycylation.	Mendes Maia T et al.[13]
**hTERT RPE-1 cells (Human)**	hBug22 siRNA	*Silencing human Bug22 gene*	Elongated primary cilium, decrease in polyglutamylation. Changes in cilia morphology and size.	

Supplementary Table 6. Primers used during the family segregation, mutational screening, and expression studies of *CFAP20* gene.

Technique	Primer Name	Sequence
Sanger sequencing	CFAP20_Ex4F	CAAGGTTGTGTGCTTCTTCC
	CFAP20_Ex4R	GAGGGTCTCGATGTAATTGGT
Custom rhAmpSeq™ Panel	CFAP20_Ex1F	/rhSeq-f/GAC ACA ACC ATT CCC CrGT CCC /GT3/
	CFAP20_Ex1R	/rhSeq-r/CCT GTA TCT ACT CCG GGrG CCT A/GT2/
	CFAP20_Ex2F	/rhSeq-f/CTA GTA TAT GAT GTC TCT GGG AGA rACA GA/GT2/
	CFAP20_Ex2R	/rhSeq-r/TCA GCC TTG TCA GAT ATT TCA GTA rCTA AT/GT3/
	CFAP20_Ex3F	/rhSeq-f/AAG AGT GGA CAC ATT CAT GTA rCAC AT/GT4/
	CFAP20_Ex3R	/rhSeq-r/AGA GTC CAG GAG TGT GTT TAT TArG TAT T/GT1/
	CFAP20_Ex4aF	/rhSeq-f/CTA TCC CCA ACC CAG GGrC TCT A/GT1/
	CFAP20_Ex4aR	/rhSeq-r/CTT CAT CTG CAC CAT GCC rCAT GC/GT2/
	CFAP20_Ex4bF	/rhSeq-f/TGT GCT TCT TCC AAG ACA GArG CTA G/GT1/
	CFAP20_Ex4bR	/rhSeq-r/AAT CTG GTT CCA GCC GTC rATC CA/GT2/
	CFAP20_Ex5F	/rhSeq-f/ACT CAC TGG TGG ACC TTrC CTC T/GT2/
	CFAP20_Ex5R	/rhSeq-r/CGC CTC TCA AAA GAA GCA TTrG CCT T/GT2/
	CFAP20_Ex6F	/rhSeq-f/AGC AAC ACC AAG TAT AAA TAA CAG AArC TAC A/GT2/
	CFAP20_Ex6R	/rhSeq-r/TGT GAC ACT GAC CAT CTA TTT TCT ArGT AAC /GT1/
RT-PCR	CFAP20_ RT_Ex4F	CCGACGGGTTTACTTCTCAG
	CFAP20_RT_Ex6R	TTTGCATCGTCCTCCACATA

The rhAmpSeq Design Tool (IDT) was used for the primer design of the custom rhAmpSeq™ Panel.

Supplementary references

1 Leman, R. *et al.* Novel diagnostic tool for prediction of variant spliceogenicity derived from a set of 395 combined in silico/in vitro studies: an international collaborative effort. *Nucleic acids research* **46**, 7913-7923, doi:10.1093/nar/gky372 (2018).

2 Moles-Fernández, A. *et al.* Computational Tools for Splicing Defect Prediction in Breast/Ovarian Cancer Genes: How Efficient Are They at Predicting RNA Alterations? *Frontiers in genetics* **9**, 366, doi:10.3389/fgene.2018.00366 (2018).

3 Tang, R., Prosser, D. O. & Love, D. R. Evaluation of Bioinformatic Programmes for the Analysis of Variants within Splice Site Consensus Regions. *Advances in bioinformatics* **2016**, 5614058, doi:10.1155/2016/5614058 (2016).

4 Jaganathan, K. *et al.* Predicting Splicing from Primary Sequence with Deep Learning. *Cell* **176**, 535-548 e524, doi:10.1016/j.cell.2018.12.015 (2019).

5 Nalpathamkalam, T., Derkach, A., Paterson, A. D. & Merico, D. Genetic Analysis Workshop 18 single-nucleotide variant prioritization based on protein impact, sequence conservation, and gene annotation. *BMC proceedings* **8**, S11, doi:10.1186/1753-6561-8-s1-s11 (2014).

6 Ng, P. C. & Henikoff, S. SIFT: Predicting amino acid changes that affect protein function. *Nucleic acids research* **31**, 3812-3814, doi:10.1093/nar/gkg509 (2003).

7 Stone, E. A. & Sidow, A. Physicochemical constraint violation by missense substitutions mediates impairment of protein function and disease severity. *Genome research* **15**, 978-986, doi:10.1101/gr.3804205 (2005).

8 Tavtigian, S. V., Greenblatt, M. S., Lesueur, F. & Byrnes, G. B. In silico analysis of missense substitutions using sequence-alignment based methods. *Human mutation* **29**, 1327-1336, doi:10.1002/humu.20892 (2008).

9 Mather, C. A. *et al.* CADD score has limited clinical validity for the identification of pathogenic variants in noncoding regions in a hereditary cancer panel. *Genetics in medicine : official journal of the American College of Medical Genetics* **18**, 1269-1275, doi:10.1038/gim.2016.44 (2016).

10 Meng, D., Cao, M., Oda, T. & Pan, J. The conserved ciliary protein Bug22 controls planar beating of Chlamydomonas flagella. *Journal of cell science* **127**, 281-287, doi:10.1242/jcs.140723 (2014).

11 Yanagisawa, H. A. *et al.* FAP20 is an inner junction protein of doublet microtubules essential for both the planar asymmetrical waveform and stability of flagella in Chlamydomonas. *Mol Biol Cell* **25**, 1472-1483, doi:10.1091/mbc.E13-08-0464 (2014).

12 Laligné, C. *et al.* Bug22p, a conserved centrosomal/ciliary protein also present in higher plants, is required for an effective ciliary stroke in Paramecium. *Eukaryotic cell* **9**, 645-655, doi:10.1128/ec.00368-09 (2010).

13 Mendes Maia, T., Gogendeau, D., Pennetier, C., Janke, C. & Basto, R. Bug22 influences cilium morphology and the post-translational modification of ciliary microtubules. *Biology open* **3**, 138-151, doi:10.1242/bio.20146577 (2014).
