# Supplementary material for: A comprehensive WGS-based pipeline for the identification of new candidate genes in inherited retinal dystrophies
Source: NPJ Genom Med. 2022 Mar 4;7:17. doi: 10.1038/s41525-022-00286-0 (PMC8897414; doi:10.1038/s41525-022-00286-0)
Supplement: Supplementary file 2 — Reporting summary [file 41525_2022_286_MOESM2_ESM.pdf]

nature research

Corresponding author(s): Guillermo Antiñolo

Last updated by author(s): Jan 14, 2022

Reporting Summary

Nature Research wishes to improve the reproducibility of the work that we publish. This form provides structure for consistency and transparency in reporting. For further information on Nature Research policies, see our Editorial Policies and the Editorial Policy Checklist.

Statistics

For all statistical analyses, confirm that the following items are present in the figure legend, table legend, main text, or Methods section.

n/a	Confirmed	
☐	☒	The exact sample size (*n*) for each experimental group/condition, given as a discrete number and unit of measurement
☐	☒	A statement on whether measurements were taken from distinct samples or whether the same sample was measured repeatedly
☐	☒	The statistical test(s) used AND whether they are one- or two-sided<br>*Only common tests should be described solely by name; describe more complex techniques in the Methods section.*
☒	☐	A description of all covariates tested
☐	☒	A description of any assumptions or corrections, such as tests of normality and adjustment for multiple comparisons
☒	☐	A full description of the statistical parameters including central tendency (e.g. means) or other basic estimates (e.g. regression coefficient) AND variation (e.g. standard deviation) or associated estimates of uncertainty (e.g. confidence intervals)
☒	☐	For null hypothesis testing, the test statistic (e.g. *F*, *t*, *r*) with confidence intervals, effect sizes, degrees of freedom and *P* value noted<br>*Give P values as exact values whenever suitable.*
☒	☐	For Bayesian analysis, information on the choice of priors and Markov chain Monte Carlo settings
☒	☐	For hierarchical and complex designs, identification of the appropriate level for tests and full reporting of outcomes
☒	☐	Estimates of effect sizes (e.g. Cohen's *d*, Pearson's *r*), indicating how they were calculated

Our web collection on statistics for biologists contains articles on many of the points above.

Software and code

Policy information about availability of computer code

Data collection	Different Illumina plataforms (MySeq, NextSeq500, HiSeqX)
Data analysis	Alamut Batch v1.11, SigmaPlot v14, Bystro Genomics, Combined Annotation Dependent Depletion (CADD) v1.6, Estimation by Read Depth with Single-nucleotide variants (ERDS) v1.1, Annotation and Ranking of Human Structural Variations (AnnotSV), VCF sort tool (VCFtools), VCF combine tool and VCF-BED intersect tool (vcflib v1.0.0_rc3),VarSome v10.1, Jalview v2.11.1.0, PyMOL Molecular Graphics System v1.8, Cytoscape v3.8.0, cellSens Dimension software, MiSeq Reporter software v2.6, DNASTAR Lasergene Software v8.1.5.

For manuscripts utilizing custom algorithms or software that are central to the research but not yet described in published literature, software must be made available to editors and reviewers. We strongly encourage code deposition in a community repository (e.g. GitHub). See the Nature Research guidelines for submitting code & software for further information.

Data

Policy information about availability of data

All manuscripts must include a data availability statement. This statement should provide the following information, where applicable:

- Accession codes, unique identifiers, or web links for publicly available datasets
- A list of figures that have associated raw data
- A description of any restrictions on data availability

The authors confirm that the data supporting the findings of this study are available within the article and its supplementary materials. The Whole-genome sequencing data are not publicly available due to families enrolled in this study did not provide additional consent to share raw dataset in a public repository. Additional specific variant information is available on request from the corresponding authors (G.A and S.B.).

Field-specific reporting

Please select the one below that is the best fit for your research. If you are not sure, read the appropriate sections before making your selection.

☒ Life sciences ☐ Behavioural & social sciences ☐ Ecological, evolutionary & environmental sciences

For a reference copy of the document with all sections, see nature.com/documents/nr-reporting-summary-flat.pdf

Life sciences study design

All studies must disclose on these points even when the disclosure is negative.

Sample size	This study involved 429 individuals grouped in three different cohorts: the training cohort with 209 solved IRD patients, the validation cohort composed by 50 IRD patients, 47 patients with neurological disorders and 109 hereditary cancer patients, and the discovery cohort involved 14 patients belonging to 7 unsolved IRD families. Additionally, 264 unsolved IRD individuals from our cohort were collected in order to conduct the mutational screening of the novel candidate genes.
Data exclusions	No data were excluded from the analyses.
Replication	All results are repeatable. First, we sequenced DNA of 415 patients through targeted genes sequencing, 14 patients trough whole genome sequencing and 264 patients underwent Custom Amplicon Sequencing. All candidate variants in this study were verified by Sanger sequencing.
Randomization	n/a
Blinding	The selection of the patients included in the validation cohort (n=206) was conducted following the blind method.

Reporting for specific materials, systems and methods

We require information from authors about some types of materials, experimental systems and methods used in many studies. Here, indicate whether each material, system or method listed is relevant to your study. If you are not sure if a list item applies to your research, read the appropriate section before selecting a response.

Materials & experimental systems

n/a	Involved in the study	
☐	☒	Antibodies
☒	☐	Eukaryotic cell lines
☒	☐	Palaeontology and archaeology
☒	☐	Animals and other organisms
☐	☒	Human research participants
☒	☐	Clinical data
☒	☐	Dual use research of concern

Methods

n/a	Involved in the study	
☒	☐	ChIP-seq
☒	☐	Flow cytometry
☒	☐	MRI-based neuroimaging

Antibodies

Antibodies used	Anti-GTL3 antibody: Rabbit polyclonal to GTL3 (Abcam, ab225952); Horseradish peroxidase polymer conjugated secondary antibodies (Visualization reagent, Agilent, CA, USA)
Validation	The validation of the primary antibody was performed by the Andalusian Public Health System Biobank and ISCIII-Red de Biobancos PT17/0015/004 using standard procedures. Sections were incubated with a 1:400 dilution of primary antibody (Abcam, ab225952) for 1 h at room temperature in a humid chamber.

Human research participants

Policy information about studies involving human research participants

Population characteristics	- The training cohort was composed by genetically diagnosed IRD patients with ACMG-classified mutations score 4 or 5.<br>- The validation cohort was composed by genetically diagnosed patients from IRD (n=33), hereditary cancer (n=109), and neurological diseases (n=47). In addition, the sub-cohort of IRD patients included 17 patients without a genetic diagnosis to conduct a blind trial.<br>-The discovery cohort was composed by 9 IRD affected individuals and 5 unaffected relatives belonging to 7 genetically unsolved families.<br>- The mutational screnning cohort was composed by 264 unsolved IRD patients.

Recruitment	All patients were recruited from the Department of Maternofetal Medicine, Genetics and Reproduction of the University Hospital Virgen del Rocio of Seville in the period from 1990 to 2020.
Ethics oversight	The research was conducted in accordance with the tenets of the Declaration of Helsinki, and all experimental protocols were approved by the Institutional Review Board of the University Hospitals Virgen del Rocio and Virgen Macarena (Spain).

Note that full information on the approval of the study protocol must also be provided in the manuscript.
